# Predicting Cross-Species Infection of Swine Influenza Virus with Representation Learning of Amino Acid Features

**DOI:** 10.1155/2021/6985008

**Published:** 2021-10-11

**Authors:** Zheng Kou, Junjie Li, Xinyue Fan, Saeed Kosari, Xiaoli Qiang

**Affiliations:** Institute of Computing Science and Technology, Guangzhou University, Guangzhou 510006, China

## Abstract

Swine influenza viruses (SIVs) can unforeseeably cross the species barriers and directly infect humans, which pose huge challenges for public health and trigger pandemic risk at irregular intervals. Computational tools are needed to predict infection phenotype and early pandemic risk of SIVs. For this purpose, we propose a feature representation algorithm to predict cross-species infection of SIVs. We built a high-quality dataset of 1902 viruses. A feature representation learning scheme was applied to learn feature representations from 64 well-trained random forest models with multiple feature descriptors of mutant amino acid in the viral proteins, including compositional information, position-specific information, and physicochemical properties. Class and probabilistic information were integrated into the feature representations, and redundant features were removed by feature space optimization. High performance was achieved using 20 informative features and 22 probabilistic information. The proposed method will facilitate SIV characterization of transmission phenotype.

## 1. Introduction

The influenza A virus (family: Orthomyxoviridae) genome contains eight segmental RNAs [1]. The hemagglutinin (HA) gene is located in the fourth segment, and the neuraminidase (NA) gene is in the sixth segment. According to the antigenic characteristics of HA and NA, the influenza A virus has 18 HA subtypes and 11 NA subtypes [2–4]. Besides the fast mutation rates of viral genes, segmental reassortments of viral genomes facilitate the occurrence of novel virus with the changes of host barriers [5, 6]. The 1957 pandemic was caused by an influenza A (H1N1) virus, which has a genome that contains segments 2, 4, and 5 from the avian influenza virus, whereas the 1968 pandemic was caused by an influenza A (H3N2) virus, which has a genome that contains segments 2 and 4 from the avian influenza virus [1].

Swine influenza virus (SIV) is an influenza A virus that causes acute respiratory infectious disease of swine [7]. There are three main SIV subtypes circulating worldwide (H1N1, H1N2, and H3N2), and these subtypes can be subdivided into different genotypes, including the classical H1N1, avian-like H1N1, human-like H3N2, reassorted H3N2, and H1N2 subgroups [8–10]. SIV genotypes are diverse, and its gene pool in nature is heterogeneous. SIV can cross the species barriers unforeseeably and directly infect humans. The 2009 H1N1 pandemic killed more than 18,000 people, and the viral pathogen during the pandemic was a novel SIV that was produced by genome reassortment between genome-reassorted swine viruses from America and Europe [11–13]. H3N2 variant viruses (H3N2v), which have segment 7 from the 2009 H1N1 pandemic virus in their genomes, were identified in swine in 2010 and first detected in people in 2011 [14]. Clinical cases of H1N1 variant viruses (H1N1v) and H1N2 variant viruses (H1N2v) were also been reported after 2011 [15]. SIVs are a huge challenge for human public health and may trigger pandemic risk.

Computational bioinformatics tools are needed to predict transmission phenotype and pandemic risk of SIVs. For this purpose, machine-learning methods may be ideal tools [16–18]. Machine learning techniques have great potential for virus screening because they can use viral protein sequences as input without the need for prior knowledge. In this paper, we propose a feature representation algorithm to predict cross-species infection of SIVs. The algorithm includes the sequence-based feature descriptors to build a comprehensive predictive model with sufficient information from different aspects. Sequence-based features with class information or probabilistic information are learnt from well-trained random forest (RF) classifiers that can learn a set of features. The dimension of the feature space was reduced using the minimum redundance maximum relevance (mRMR) method to obtain the most informative features and distinguish SIVs with different transmission phenotypes.

To identify SIVs capable of interspecies transmission, we constructed a predictor with two predictive models that were trained using 20 features based on class information or 22 features based on probabilistic information under the RF classifier. The predictor with the feature representation learning achieved a high prediction performance. This study provides an important tool in predicting cross-species infection of SIVs for public health.

## 2. Materials and Methods

### 2.1. Data

Viral sequences of influenza viruses isolated from swine and human were downloaded from the GISAID EpiFlu public database (http://platform.gisaid.org/epi3/frontend) [2, 3]. GISAID deposits high-quality genomic sequences along with their clinical information in the database. Since sequence redundancy was very high and genome coverage varied greatly, raw data were filtered using public bioinformatics tools and algorithms (Table S1).

We obtained 5860 SIVs and 44,623 human influenza viruses from the GISAID database on 21 March 2019. The dataset included all of the 11 influenza virus proteins (PB2, PB1, PB1-F2, PA, HA, NP, NA, M1, M2, NS1, and NEP) encoded in eight genome segments. Strains without any of the 11 protein sequences or without subtype information were excluded. Amino acid positions in the 11 proteins were determined using the multiple sequence alignment tool MUSCLE [19]. Strains with more than three amino acids missing at the terminal ends of the viral proteins were removed, and if there were only a few missing residues, they were added according to those in viral proteins with highest identity. We used the fast-clustering algorithm of the CD-Hit tool to reduce the redundancy in the dataset [20]. Ambiguous amino acid residues, such as X and B, were likely caused by sequencing error and were replaced by those in viral protein with highest identity. Strains with large numbers of ambiguous residues in viral protein were also removed.

The final dataset for predicting cross-species infection contained two categories of viruses: (1) 769 viruses isolated from human (positive sample; H1N1, H1N2, H2N2, and H3N2 subtypes); (2) 1133 influenza viruses isolated from swine (negative sample; H1N1, H1N2, and H3N2 subtypes). The positive samples were composed by seasonal human influenza virus, 2009 pandemic swine virus, and variant swine virus isolated from human. Since these viruses could be also isolated from swine [1], they were excluded from the negative samples according to the similarity of genome sequence. Information about the 1902 strains is summarized in Table S1.

### 2.2. Signature Amino Acid Positions Based on Entropy

Most of the amino acid residues in the viral proteins were conserved. To reduce the computing complexity, amino acid residues were filtered by the entropy measure at each position of the 11 viral proteins. For a given position *i*, the entropy value was computed using the formula [21]: *E*_*i*_ = −∑_*j*=1_^20^*P*_*i*,*j*_log(*P*_*i*,*j*_), where *P*_*i*,*j*_ is the observed probability of amino acid *j* at position *i*. High entropy values indicate high amino acid mutation rates at the corresponding position. We set the threshold of entropy difference as 1.5 and obtained 36 signature positions, and therefore, each strain was represented by a list of 36 amino acid residues in the screened positions.

### 2.3. Representation of Signature Amino Acid Set

Mutations in the viral proteins determine the pathogenicity or virulence of SIVs [1]. After obtaining the entropy ranking for each position, 36 significant amino acids were screened. Six encoding algorithms for compositional information, position-specific information, and physicochemical properties of amino acids were used to explore the key information required for high-quality predictions [16]. The encoding algorithms for the signature amino acid set to transform SIV into fix-length vectors are detailed below.

#### 2.3.1. Amino Acid Composition

The amino acid composition (AAC) is a 20-dimension vector as usual. Because the gaps (deletion or insertion) in viral proteins occurred frequently during the evolution of SIV, we defined the AAC as a 21-dimension vector to represent the frequency of the 20 amino acid residues and one gap in the 36 signature positions of the viral proteins. For example, if the amino acid type *i* occurs *n*_*i*_ times in the amino acid set of a specific virus, the frequency of *i* is denoted as *f*(*i*) = *n*_*i*_/36. A 21-dimensional feature vector that represents the frequencies of the 20 different amino acids and one gap was obtained for each strain.

#### 2.3.2. Parallel Correlation-Based Pseudo-Amino-Acid Composition

Parallel correlation-based pseudo-amino-acid composition (PC-PseAAC) method was used to compute the parallel correlation of any two amino acids in the 36 signature amino acid positions in the viral protein sequences [22]. For a virus D, the PC-PseAAC feature vector was defined by

(1) $$\begin{array}{c} \text{PC}-\text{PseAAC}={\left[p_{1},\cdots ,p_{21},p_{21+1},\cdots ,p_{21+\lambda }\right]}^{T}, \end{array}$$

where

(2) $$\begin{array}{c} p_{u}=\left\{\begin{array}{cc} \frac{f_{u}}{\sum_{i=1}^{21}f_{i}+0.05\sum_{j=1}^{\lambda }\theta_{j}}, & 1\leq u\leq 21,\\ \frac{0.05\theta_{u-21}}{\sum_{i=1}^{21}f_{i}+0.05\sum_{j=1}^{\lambda }\theta_{j}}, & 21+1\leq u\leq 21+\lambda , \end{array}\right. \end{array}$$

where *u* is an integer, *fi* (1 ≤ *i* ≤ 21) represents the normalized occurrence frequency of the 20 amino acids and one gap in virus *D*, *λ* is the highest tier of the correlation along *D*, and *θj* is the correlation function that measures the *j*-tier sequence-order correlation between all the *j*-th most contiguous residues along D. The *θj* function is given as

(3) $$\begin{array}{c} \theta_{j}=\frac{1}{36}\;\overset{L}{\underset{i=1}{\sum }}\frac{1}{5}\overset{5}{\underset{m=1}{\sum }}{\left[H_{m}\left(A_{i+j}\right)-H_{m}\left(A_{i}\right)\right]}^{2}\;, \end{array}$$

where *Hm* (*Ai*)(*m* = 1, 2, 3, 4, 5) represents the five amino acid factors that correspond to the *i*-th amino acid *Ai* in virus D, respectively [23]. If *i* + *j* is >36, then *i* + *j* equals *i* + *j* − 36. The five factors for gap (deletion or insertion) were simply set to zero.

#### 2.3.3. G-Gap Dipeptide Composition

The G-gap dipeptide composition (GGAP) is the dipeptide composition coupled with local order information of any two interval residues among the 36 amino acid residues of the 11 viral proteins for each virus. The GGAP is commonly used feature descriptor for sequence analysis and model construction. In this paper, GGAP is a 441-dimension vector that represents the frequency of dipeptide comprising 20 amino acid residues and one gap. It is defined as

(4) $$\begin{array}{c} \text{GGAP}\left(g\right)=\left(p_{1}^{g},p_{2}^{g},\cdots ,p_{441}^{g}\right), \end{array}$$

where *p*_*i*_^*g*^ is the occurrence frequency of the *i*-th (*i* = 1, 2, ⋯, 441) G-gap dipeptide, which is defined as

(5) $$\begin{array}{c} p_{i}^{g}=\frac{O_{i}^{g}}{\sum_{i=1}^{441}O_{i}^{g}}, \end{array}$$

where *O*_*i*_^*g*^ is the occurrence number of the *i*-th G-gap dipeptide in the 36 signature amino acid residues. The dimension of the GGAP feature vector is 21 × 21 = 441. Deletion or insertion is also computed.

#### 2.3.4. Twenty-Bit Features

Position-specific information and physicochemical properties were used to encode the 36 amino acid residues for each virus. Five physicochemical property descriptors of the standard amino acids were constructed, namely, polarity, secondary structure, molecular volume, codon diversity, and electrostatic charge [23]. For each descriptor, the standard amino acid alphabets were classified into three groups, and the deletion/insertion (indel) was regarded as the fourth group. Representation of 20 standard amino acids and one indel was according to the five physicochemical properties. Each residue was encoded as a 20-bit vector comprising 0/1 elements, where the position of the bit was set to 1 if the residue belongs to the corresponding group; otherwise, it was 0. Given the amino acid augment approach, the top *k* residues with the highest entropy values were selected. The dimension of the feature vector was 20 × *k*.

#### 2.3.5. Twenty-One-Bit Features

Twenty-one-bit feature was like a one-hot encoding. In this algorithm, each amino acid residue is transformed into a 21-bit 0/1 vector. (e.g., Ala by 1,0,0,0,0,0,0,0,0,0,0,0,0,0,0,0,0,0,0,0,0; indel by 0,0,0,0,0,0,0,0,0,0,0,0,0,0,0,0,0,0,0,0,1). Given the amino acid augment approach, each strain with the top *k* residues was represented by a 21 × *k* dimensional feature vector.

#### 2.3.6. Overlapping Property Features

This algorithm divided the 20 standard amino acids and one gap (indel) into 11 different groups according to physicochemical properties. The distribution of the 20 stranded amino acids in the 10 groups can overlap [24]. The 10 amino acid groups were aromatic = {F, Y, W, H}, negative = {D, E}, positive = {K, H, R}, polar = {N, Q, S, D, E, C, T, K, R, H, Y, W}, hydrophobic = {A, G, C, T, I, V, L, K, H, F, Y, W, M}, aliphatic = {I, V, L}, tiny = {A, S, G, C}, charged = {K, H, R, D, E}, small = {P, N, D, T, C, A, G, S, V}, and proline = {A, S, G, C}. Indels form the 11th group. Each amino acid residue was represented by an 11-dimensional 0/1 vector. The position of the vector was set to 1 if the residue belongs to the physicochemical property group; otherwise, it was 0. Given the amino acid augment approach, the top *k* residues with the highest entropy values were selected. The amino acid augment was encoded with an 11 × *k* feature vector.

### 2.4. Framework of Feature Representation Learning

**Figure 1:**
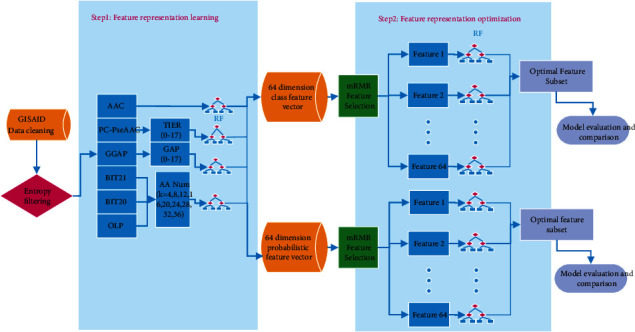
Flowchart of representation learning of amino acid features. After data cleaning, 36 signature amino acid positions based on entropy were screened. Six encoding algorithms with the change of parameter were used to explore the key information. All the 64 descriptors in the feature pool were used to train and predict with the RF models, and two types of predictions were achieved to be further optimized. Each swine virus was eventually represented by two optimized feature vectors, “class” and “prob.” Finally, the predictive models were built and compared.

The framework of the feature representation learning algorithm, which includes two main steps, feature representation learning and feature representation optimization, is shown in Figure 1. Firstly, feature representations from a set of feature descriptors are generated using the RF classifier systems. Secondly, the feature representations learnt from the first step are optimized to yield informative feature subsets. The two-step feature representation learning procedure was as follows [16].

#### 2.4.1. Feature Representation Learning

The six feature encoding algorithms were AAC, PC-PseAAC, GGAP, 20-bit features (BIT20), 21-bit features (BIT21), and overlapping property features (OLP), all of which are described above. A feature pool was built to generate as much information as possible in the predicting models with different parameters. For example, *k* is a common parameter for BIT20, BIT21, and OLP. Because the 36 significant amino acids were screened after the entropy ranking was obtained, we set *k* as 4-36 by step 4. The maximum *k* value was set as 36 because there were 36 signature positions, and therefore, a total of 27 feature descriptors are obtained for BIT20, BIT21, and OLP. A similar procedure was used for PC-PseAAC and GGAP. With the use of different parameters, a total of 64 feature descriptors were in the feature pool. Information about all the feature descriptors is provided in Table 1.

Before the optimization of feature representation, two types of predictions were used to fulfill the learning. All the 64 descriptors in the feature pool were used to train and predict with the RF models, and two types of predictions were achieved. The first prediction type was the class label (positive or negative): positive samples (swine viruses with the phenotype of cross-species infection) were marked as 1, and negative samples (swine viruses without the phenotype of cross-species infection) were marked as 0. The second prediction type was the pseudo probability that a sample belongs to a certain class (positive or negative). For each prediction type, all 64 outputs computed by the 64 RF models were concatenated as a new feature vector. Each swine virus was eventually represented by two 64-dimensional feature vectors, which were marked “class” and “prob,” respectively. Feature vector “class” comprised the class information learnt from the original feature pool, and feature vector “prob” comprised the probabilistic information. Fast speed for computation was expected for the first type models while high performance for prediction accuracy was expected for the second type models.

#### 2.4.2. Feature Representation Optimization

The two prediction types were further optimized to improve their feature representation ability. A well-known feature selected method, mRMR, was used to rank the features of the “class” and “prob” information [25]. The mRMR method uses the mutual information to maximize the mutual information between the joint distribution of the selected features and the class labels and minimizes the redundancy between the selected features. The mRMR method was used to optimize the feature representations and obtain the feature list ranked by their importance scores. The sequential forward search (SFS) strategy was used to increase the features from the ranked feature list one by one [16]. After training the RF classifier, the feature subset with the best performance was considered as the optimal subset. We obtained 20 optimal features for “class” and the 25 optimal features for “prob.”

### 2.5. RF Algorithm

An RF algorithm was used to obtain two types of feature vectors and construct models of prediction for cross-species infection of SIVs. RF machine-learning algorithms are robust and have been used widely to model biology data [4]. The RF behaves like an ensemble algorithm and proposes a set of decision trees by random feature selection. We used the RF algorithm in the R environment in this study [26]. All the experiments were done using version 3.5.0 of R with the default parameters (tree number = 500).

### 2.6. Evaluation Metrics

We used four commonly used metrics to evaluate the model performance, namely, sensitivity (SN), specificity (SP), accuracy (ACC), and Mathew's correlation coefficient (MCC) as follows:

(6) $$\begin{array}{c} \left\{\begin{array}{c} \begin{array}{c} \text{SN}=\frac{\text{TP}}{\text{TP}+\text{FN}}\times 100\%,\\ \text{SP}=\frac{\text{TN}}{\text{TN}+\text{FP}}\times 100\%, \end{array}\\ \text{ACC}=\frac{\text{TP}+\text{TN}}{\text{TP}+\text{TN}+\text{FP}+\text{FN}}\times 100\%,\\ \text{MCC}=\frac{\text{TP}\times \text{TN}+\text{FP}\times \text{FN}}{\sqrt{\left(\text{TP}+\text{FN}\right)\;\left(\text{TP}+\text{FP}\right)\;\left(\text{TN}+\text{FN}\right)\;\left(\text{TN}+\text{FP}\right)}}\times 100\%, \end{array}\right. \end{array}$$

where TP indicates true positive, the number of correctly predicted true strains with the phenotype of human infection; TN indicates true negative, the number of correctly predicted true strains without the phenotype of human infection; FP indicates false positive, the number of strains without the phenotype of human infection predicted to be strains with the phenotype of human infection; and FN is false negative, the number of strains with the phenotype of human infection predicted to be strains without the phenotype of human infection. The sensitivity and specificity metrics measure the predictive ability of a model in positive and negative cases, respectively. The other two measures, ACC and MCC, were used to evaluate the overall performance of the models. For these four metrics, high scores indicate high performance of the models.

The receiver operating characteristic (ROC) curve, which is used to evaluate the overall performance of a binary classifier system [27], was also used in this study. The ROC curve is generated by plotting the true positive rate against the false positive rate under different classification thresholds. We also calculated the area under ROC curve (AUC) to evaluate the predictive performance of the models. AUC values range from 0.5 to 1.

### 2.7. Tenfold Cross-Validation Method

The 10-fold cross-validation method was used to evaluate the predictive performance of the models. The models were trained on 692 positive samples and 1019 negative samples that were selected randomly from the cleaned dataset. The remaining 10% of samples (77 positive and 114 negative) were used as an independent test dataset to assess the performances of the classifiers. This process was repeated 10 times, and the 10 results are averaged to obtain the final evaluation of prediction performance.

## 3. Results and Discussion

### 3.1. Signature Position of SIV

After elimination of redundancy and other necessary cleaning of viral data from the GISAID database, the final dataset for the prediction of cross-species infection contained two categories of viruses: 769 viruses isolated from human and 1133 viruses isolated from swine. The 769 human viruses were considered as positive samples because they were verified to have the ability of infection among humans. The 1133 swine viruses were considered as negative samples. Information about these virus strains is summarized in Table S1.

To screen the signature position, the entropies in each position of the 11 viral proteins were calculated, respectively. As shown in Table 2, the HA protein contained the highest number of selected amino acid residues (25/36), which is consistent with the known role of HA mainly in receptor-binding and fusion activity for cross-species infection of SIVs. Positions HA102-HA290 are located in or close to the host receptor binding region [28, 29], and HA163 and HA189 are related to the specificity of receptor binding [30, 31]. The signature positions were verified to be related with the mechanism of interspecies transmission or high efficiency of transmission among humans, which would rationalize the model and benefit predicting accuracy.

### 3.2. Optimal Feature Representations

**Figure 2:**
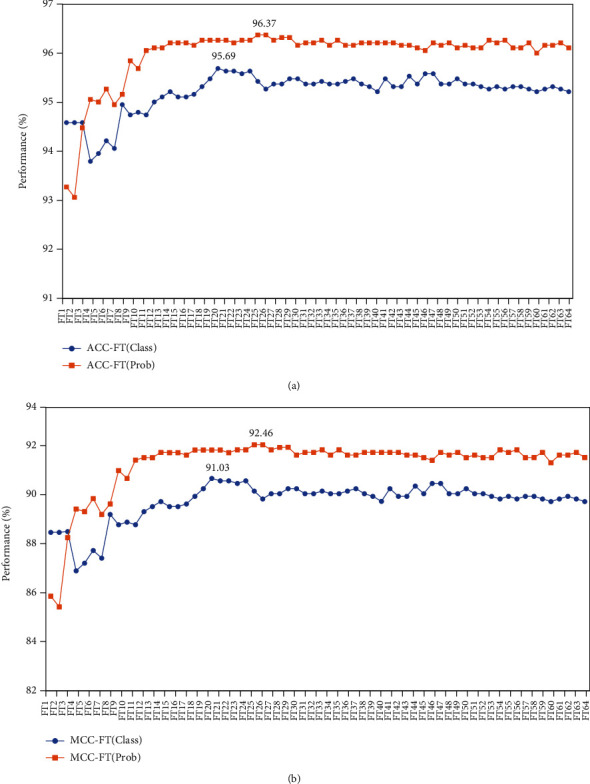
Feature representation optimization with the mRMR algorithm. (a) The SFS curves for ACC of “class” and “prob” feature. The feature number (1-64) and the accuracy were represented by the *x*- and *y*-axis. (b) The SFS curves for MCC of “class” and “prob” feature. The feature number (1-64) and the coefficient were represented by the *x*- and *y*-axis. The “class” and “prob” features were marked by the blue and yellow color.

The mRMR feature ranking algorithm was used to select the 64-dimensional feature vector, which comprised the predictions from the 64 RF models (Figure 1). A ranked feature list for the 64 features was generated after sorting their importance scores from the mRMR algorithm. The sequential forward search strategy was proposed to explore the optimal feature representations from the ranked list of 64 features. The features were increased one by one according to the sequence in the list, and the RF classifiers were trained. The influenza virus data were tested with the 10-fold cross-validation method. The sequential forward search curves for the ACC and MCC metrics were drawn to find the optimized feature (Figure 2). For the class features, the RF classifier performed best with maximum ACC and MCC of 95.69% and 91.03%, respectively, when the feature number 20 was selected (Figures 2(a) and 2(b)). This result indicates that the first 20 features from the ranked feature list had the optimal representation ability to distinguish swine viruses with the ability of cross-species infection. For the probabilistic features, the RF classifier performed best with the first 25 features (ACC of 96.37% and MCC of 92.46%; Figures 2(a) and 2(b)). The screened 20 class and 25 probabilistic features were used to build the predictive classifiers of cross-species infection.

### 3.3. Comparison of Optimal Feature Representations with Individual Descriptors

Using the class feature, optimal representation vectors with 20 dimensions were obtained from 20 individual feature descriptors. The predictive performance of the optimal feature was compared with the six top individual descriptors to evaluate the learning ability of the feature representation. The 10-fold cross-validation tests were fulfilled based on the dataset.

**Figure 3:**
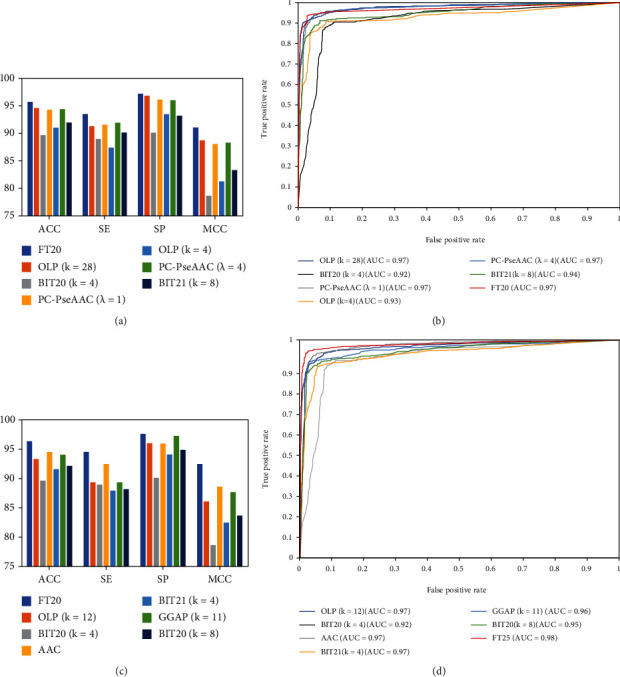
Performance of the optimal features: (a) performances of the optimal “class” features and the top 6 individual descriptors; (b) ROC curves of the optimal “class” features and the top 6 individual descriptors; (c) performances of the optimal “prob” features and the top 6 individual descriptors; (d) ROC curves of the optimal “prob” features and the top 6 individual descriptors.

The performances of the optimal class features and the compared individual features are illustrated in Figure 3(a), and the ROC curves were shown in Figure 3(b). The optimal features gave the best predictive performance with maximum ACC and MCC of 95.68% and 91.03%, respectively, which are higher than the values obtained with the second-best feature descriptor BIT20 (*k* = 4) (Figure 3(a)). The AUC (0.97) obtained using our feature descriptor was better than that of BIT20 (*k* = 4; *AUC* = 0.91). Notably, only 20 features were used for optimal feature, whereas BIT20 (*k* = 4) used 80 features. Moreover, the 25 optimal feature representations based on probabilistic information were compared with the individual feature descriptors. The performances of the optimal probabilistic features and the compared individual features are shown in Figure 3(c), and the ROC curves are shown in Figure 3(d). The results were consistent and indicated that the probabilistic feature representations outperformed the other six feature descriptors. The optimal features gave the best predictive performance with maximum ACC and MCC of 96.37% and 92.46%, respectively, which are higher than those of the six individual features.

### 3.4. Comparison of Class and Probabilistic Information

Influenza viruses were represented by class and probabilistic information, and their feature vectors comprised the predictions of the 64 RF models to encode signature positions of 36 amino acids. The performance of different information to predict cross-species infection of swine influenza virus was evaluated. As shown in Table 3, the feature vector using probabilistic information outperformed the feature vector using class information. The overall performance based on probabilistic information had ACC and MCC values of 95.95% and 91.59%, respectively, whereas the overall performance based on class information had ACC and MCC values of 95.22% and 90.05%, respectively. The performances of the two optimal feature vectors also are shown in Table 3. The overall 64-dimensional feature vectors encoded with class and probabilistic information were compared with the optimal features. After ranking by the mRMR algorithm, the performance based on the optimal probabilistic information increased from 95.95% to 96.37% for ACC and from 91.59% to 92.46% for MCC, and the performance based on class information increased from 95.22% to 95.69% for ACC and from 90.05% to 91.03% for MCC. These results confirmed that the probabilistic feature identified infection better than the class feature. However, both feature types had predictive power for cross-species infection and were used to construct predictive models.

### 3.5. Comparison of Feature Representation Learning with Ensemble Learning

Traditional ensemble learning methods combine predicting results from multiple models to make decisions and for classification. The feature representation learning used and optimized the predictions of the 64 RF models to obtain a predicting model that was similar to traditional ensemble learning models. Two types of feature representation learning (class information learning and probabilistic information) were compared with two classical ensemble learning methods (majority voting and probability averaging). Majority voting considers the majority predictions of the 64 RF models and makes predictions according to the majority rule. Probability averaging simply computes the probabilistic values of the 64 RF models and makes prediction based on the threshold. As shown in Table 4, both types of feature representation learning gave better performances than the two traditional ensemble learning methods. With the probabilistic information, the feature learning strategy had maximum ACC and MCC of 96.37% and 92.46%, respectively. The ACC and MCC obtained with our strategy were about 2% and 3% higher, respectively, than those obtained with the ensemble strategies. Based on class information, the feature learning strategy had the maximum ACC and MCC of 95.69% and 91.03%, respectively. The ACC and MMC obtained with our strategy were about 1% and 3% higher, respectively, than those obtained with the ensemble strategies. Notably, our feature learning strategy achieved a remarkable improvement, even though ensemble learning is considered an effective way to improve predictive performances.

### 3.6. Comparison of Our Predictor with Classical Classifiers

**Figure 4:**
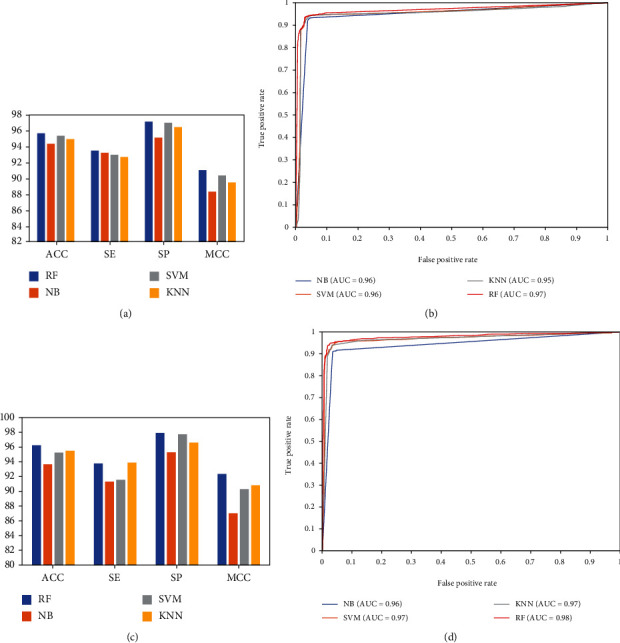
Comparison of traditional classifiers: (a) performances of the optimal “class” features with traditional classifiers; (b) ROC curves of the optimal “class” features with traditional classifiers; (c) performances of the optimal “prob” features with traditional classifiers; (d) ROC curves of the optimal “prob” features with traditional classifiers.

We used the RF algorithm and class or probabilistic information to construct predictor for SIVs. To evaluate the predictive performance of the RF method, we compared our predictor with the traditional classifiers, Support vector machine (SVM), Naïve Bayes (NB), and K-nearest neighbor (KNN), on our dataset with 10-fold cross-validation. The parameters for these classifiers were the same with those in the references [4] and [32]. The results showed that the RF method gave the best overall predictive performance based on the class information with maximum ACC and MCC of 95.69% and 91.03%, which were 1.32% and 2.71% higher, respectively, than those obtained with the NB method (Figures 4(a) and 4(b)). Our AUC (0.97) was better than that of KNN (*k* = 4; AUC = 0.95). We also compared our predictor with the traditional classifiers based on probabilistic information. The probabilistic feature representation outperformed the other three classifiers. The RF method gave the best overall predictive performance based on the probabilistic information with maximum ACC and MCC of 96.37% and 92.46%, which were 2.58% and 5.38% higher, respectively, than those with the NB method (Figures 4(c) and 4(d)). Our AUC (0.98) was better than that of NB (AUC = 0.96). Overall, the results show that the RF method produced better predictions of infection than the support vector machine, NB, and KNN methods.

## 4. Conclusions

A model for predicting cross-species infection of SIVs was described in the paper. The major contribution of this predictor was the set of informative features of viral proteins that were learned from a total of 64 feature descriptors, including compositional, position-specific, and physicochemical information. A feature representation learning scheme was proposed. We integrated class and probabilistic information into our feature representations and removed redundant and irrelevant features by feature space optimization to improve the feature representation ability. The ten-fold cross-validation results showed that a high predictive performance was achieved using 20 informative features and 22 probabilistic information. We compared the feature representation learning scheme with those of different learning strategies and confirmed that feature representation learning scheme gave better predictions. We anticipate that our method will be a powerful tool for large-scale identification of swine influenza viruses and will facilitate the characterization of their transmission phenotype and accelerate their applications in virology.

## Figures and Tables

**Table 1:** Summary of feature descriptors and their corresponding feature number.

Descriptors	Type	Number	Descriptors	Type	Number
1	AAC	20	33	GGAP (*g* = 13)	441
2	PseAAC (*λ* = 1)	21	34	GGAP (*g* = 14)	441
3	PseAAC (*λ* = 2)	22	35	GGAP (*g* = 15)	441
4	PseAAC (*λ* = 3)	23	36	GGAP (*g* = 16)	441
5	PseAAC (*λ* = 4)	24	37	GGAP (*g* = 17)	441
6	PseAAC (*λ* = 5)	25	38	BIT20 (*k* = 4)	80
7	PseAAC (*λ* = 6)	26	39	BIT20 (*k* = 8)	160
8	PseAAC (*λ* = 7)	27	40	BIT20 (*k* = 12)	240
9	PseAAC (*λ* = 8)	28	41	BIT20 (*k* = 16)	320
10	PseAAC (*λ* = 9)	29	42	BIT20 (*k* = 20)	400
11	PseAAC (*λ* = 10)	30	43	BIT20 (*k* = 24)	480
12	PseAAC (*λ* = 11)	31	44	BIT20 (*k* = 28)	560
13	PseAAC (*λ* = 12)	32	45	BIT20 (*k* = 32)	640
14	PseAAC (*λ* = 13)	33	46	BIT20 (*k* = 36)	720
15	PseAAC (*λ* = 14)	34	47	BIT21 (*k* = 4)	84
16	PseAAC (*λ* = 15)	35	48	BIT21 (*k* = 8)	168
17	PseAAC (*λ* = 16)	36	49	BIT21 (*k* = 12)	252
18	PseAAC (*λ* = 17)	37	50	BIT21 (*k* = 16)	336
19	PseAAC (*λ* = 18)	38	51	BIT21 (*k* = 20)	420
20	GGAP (*g* = 0)	441	52	BIT21 (*k* = 24)	504
21	GGAP (*g* = 1)	441	53	BIT21 (*k* = 28)	588
22	GGAP (*g* = 2)	441	54	BIT21 (*k* = 32)	672
23	GGAP (*g* = 3)	441	55	BIT21 (*k* = 36)	756
24	GGAP (*g* = 4)	441	56	OLP (*k* = 4)	44
25	GGAP (*g* = 5)	441	57	OLP (*k* = 8)	88
26	GGAP (*g* = 6)	441	58	OLP (*k* = 12)	132
27	GGAP (*g* = 7)	441	59	OLP (*k* = 16)	176
28	GGAP (*g* = 8)	441	60	OLP (*k* = 20)	220
29	GGAP (*g* = 9)	441	61	OLP (*k* = 24)	264
30	GGAP (*g* = 10)	441	62	OLP (*k* = 28)	308
31	GGAP (*g* = 11)	441	63	OLP (*k* = 32)	352
32	GGAP (*g* = 12)	441	64	OLP (*k* = 36)	396

**Table 2:** Amino acid set for predicting SIVs.

Num	Pro^1^	Pos^2^	Entropy	Num	Pro	Pos	Entropy	Num	Pro	Pos	Entropy
1	HA	9	1.57	13	HA	163	1.56	25	HA	401	1.51
2	HA	53	1.74	14	HA	169	1.65	26	NA	42	1.75
3	HA	78	1.56	15	HA	173	1.62	27	NA	43	1.78
4	HA	82	1.51	16	HA	189	2.17	28	NA	52	1.61
5	HA	131	1.59	17	HA	192	1.58	29	NA	93	1.77
6	HA	135	1.67	18	HA	193	1.63	30	NA	332	1.65
7	HA	137	1.57	19	HA	196	1.76	31	NA	344	1.55
8	HA	140	1.68	20	HA	199	1.62	32	NA	369	1.87
9	HA	142	1.90	21	HA	219	1.65	33	NA	385	1.74
10	Ss	144	2.15	22	HA	261	1.76	34	NA	400	1.72
11	HA	156	1.75	23	HA	269	1.54	35	NA	435	1.69
12	HA	159	1.65	24	HA	276	1.62	36	PB1-F2	21	1.52

^1^Viral protein. ^2^Position of amino acid residue as H3 subtype numbering.

**Table 3:** Results of feature representations using class information and probabilistic information.

Features	ACC	SE	SP	MCC	TP	TN	FP	FN
Class features	95.22	92.72	96.91	90.05	713	1098	35	56
Probabilistic features	95.95	93.24	97.79	91.59	717	1108	25	52
Optimal class features	95.69	93.50	97.18	91.03	719	1101	32	50
Optimal probabilistic features	96.37	94.54	97.62	92.46	727	1106	27	42

**Table 4:** Performance of feature representation learning and ensemble learning.

Learning strategies	ACC	SE	SP	MCC	TP	TN	FP	FN
Class information	95.69	93.50	97.18	91.03	719	1101	32	50
Probabilistic information	96.37	94.54	97.62	92.46	727	1106	27	42
Major voting	94.37	90.51	97.00	88.31	696	1099	34	73
Probability averaging	94.48	90.77	97.00	88.52	698	1099	34	71

## Data Availability

After the registration for any application (https://www.gisaid.org/registration/register/), the public sequences of influenza viruses used in this paper can be downloaded from the GISAID EpiFlu database (http://platform.gisaid.org/epi3/frontend) under the database access agreement (https://platform.epicov.org/epi3/frontend#5aa0ce) and with the acknowledgment GISAID data contributors (https://www.gisaid.org/help/publish-with-data-from-gisaid/). We used the Python programming language to create an easy-to-use tool that implements our predictor and handle massive data, which is freely accessible via https://github.com/kouzheng/SIVPred-FL.

## Supplementary Materials

Supplementary materials Information about the final 1902 strains is summarized in Table S1.
