# Supplementary material for: Predicting Cross-Species Infection of Swine Influenza Virus with Representation Learning of Amino Acid Features
Source: Comput Math Methods Med. 2021 Oct 11;2021:6985008. doi: 10.1155/2021/6985008 (PMC8523279; doi:10.1155/2021/6985008)
Supplement: Supplementary materials — Information about the final 1902 strains is summarized in Table S1. [file 6985008.f1.pdf]

ID	Nomenclature	Subtype	Phenotype
EPI_ISL_218602	A/swine/Dong_Nai/12-04-3/2015	H3N2	Negative
EPI_ISL_285477	A/swine/Nebraska/A02221499/2017	H3N2	Negative
EPI_ISL_195196	A/swine/Spain/16677/2011	H3N2	Negative
EPI_ISL_200850	A/swine/Kentucky/14TOSU23948/2014	H3N2	Negative
EPI_ISL_196018	A/swine/Mexico/9783350/2013	H3N2	Negative
EPI_ISL_150703	A/swine/Texas/SG1186/2004	H3N2	Negative
EPI_ISL_190476	A/swine/North_Carolina/A02077505/2015	H3N2	Negative
EPI_ISL_208064	A/swine/Manitoba/D0379/2015	H3N2	Negative
EPI_ISL_155800	A/swine/Nebraska/A01409647/2013	H3N2	Negative
EPI_ISL_243765	A/swine/Ohio/14TOSU0306/2014	H3N2	Negative
EPI_ISL_28431	A/swine/Ratchaburi/NIAH59/2004	H3N2	Negative
EPI_ISL_296391	A/swine/Iowa/A02135000/2017	H3N2	Negative
EPI_ISL_243776	A/swine/Kentucky/15TOSU18783/2015	H3N2	Negative
EPI_ISL_218603	A/swine/Dong_Nai/12-06-3/2015	H3N2	Negative
EPI_ISL_278827	A/swine/Italy/248147-8/2015	H3N2	Negative
EPI_ISL_127928	A/swine/Illinois/A00857318a/2012	H3N2	Negative
EPI_ISL_218287	A/swine/Dong_Nai/08-16-1/2015/pAEpC1	H3N2	Negative
EPI_ISL_116352	A/swine/Minnesota/A01076701/2010	H3N2	Negative
EPI_ISL_168295	A/swine/Indiana/13TOSU5869/2013	H3N2	Negative
EPI_ISL_218539	A/swine/Bac_Ninh/40-17-3/2015	H3N2	Negative
EPI_ISL_104803	A/swine/Minnesota/A01076196/2010	H3N2	Negative
EPI_ISL_311325	A/swine/Ohio/OH-18-7965/2018	H3N2	Negative
EPI_ISL_205322	A/swine/Missouri/A01795668/2015	H3N2	Negative
EPI_ISL_271066	A/swine/South_Dakota/MT_12_07_1554/2012	H3N2	Negative
EPI_ISL_218541	A/swine/Bac_Ninh/40-19-3/2015	H3N2	Negative
EPI_ISL_282519	A/swine/Manitoba/D0423/2016	H3N2	Negative
EPI_ISL_218294	A/swine/Dong_Nai/08-27-1/2015	H3N2	Negative
EPI_ISL_206672	A/swine/Indiana/A01260976/2015	H3N2	Negative
EPI_ISL_29807	A/swine/Minnesota/761/2007	H3N2	Negative
EPI_ISL_151160	A/swine/Manitoba/SG1438/2011	H3N2	Negative
EPI_ISL_282538	A/swine/British_Columbia/SD0132/2016	H3N2	Negative
EPI_ISL_218578	A/swine/Bac_Ninh/43-06-3/2015	H3N2	Negative
EPI_ISL_150397	A/swine/Indiana/A00968325/2010	H3N2	Negative
EPI_ISL_87551	A/swine/Pennsylvania/057108-1/2010	H3N2	Negative
EPI_ISL_26009	A/swine/Nordkirchen/IDT1993/2003	H3N2	Negative
EPI_ISL_24590	A/swine/Korea/CY10/2007	H3N2	Negative
EPI_ISL_218047	A/swine/Dong_Nai/09-06-1/2014	H3N2	Negative
EPI_ISL_104647	A/swine/North_Carolina/A01076178/2009	H3N2	Negative
EPI_ISL_162014	A/swine/Indiana/A00968344/2011	H3N2	Negative
EPI_ISL_271065	A/swine/Iowa/MT_12_07_4286/2012	H3N2	Negative
EPI_ISL_282737	A/swine/Manitoba/D0414/2015	H3N2	Negative
EPI_ISL_102558	A/swine/Indiana/A01049349/2010	H3N2	Negative
EPI_ISL_195989	A/swine/Mexico/7773583/2010	H3N2	Negative
EPI_ISL_207872	A/swine/Manitoba/D0320/2014	H3N2	Negative
EPI_ISL_127584	A/swine/Illinois/A01240575/2011	H3N2	Negative
EPI_ISL_278920	A/swine/Italy/319159-3/2010	H3N2	Negative
EPI_ISL_116349	A/swine/Iowa/A01076311/2010	H3N2	Negative
EPI_ISL_278804	A/swine/Quebec/1557833/2013	H3N2	Negative
EPI_ISL_195204	A/swine/England/502321/1994	H3N2	Negative
EPI_ISL_174307	A/swine/Arkansas/A01555090/2014	H3N2	Negative
EPI_ISL_207099	A/swine/Manitoba/D0260/2013	H3N2	Negative
EPI_ISL_207033	A/swine/Manitoba/D0091/2012	H3N2	Negative
EPI_ISL_256258	A/swine/Florida/A01104089/2015	H3N2	Negative
EPI_ISL_243787	A/swine/Iowa/A01781973/2016	H3N2	Negative
EPI_ISL_133383	A/swine/Germany/R83/2011	H3N2	Negative
EPI_ISL_195197	A/swine/Spain/18512/2011	H3N2	Negative
EPI_ISL_151261	A/swine/North_Carolina/SG1462/2004	H3N2	Negative

EPI_ISL_161643	A/swine/Bac_Ninh/159/2012	H3N2	Negative
EPI_ISL_24588	A/swine/Korea/CY07/2007	H3N2	Negative
EPI_ISL_161660	A/swine/Bac_Ninh/386/2012	H3N2	Negative
EPI_ISL_84913	A/swine/Italy/1850/1977	H3N2	Negative
EPI_ISL_138209	A/swine/Iowa/A01432395/2013	H3N2	Negative
EPI_ISL_266544	A/swine/Iowa/A01667087/2017	H3N2	Negative
EPI_ISL_167331	A/swine/Kansas/A01477353/2014	H3N2	Negative
EPI_ISL_256555	A/swine/Illinois/A01672426/2017	H3N2	Negative
EPI_ISL_218600	A/swine/Dong_Nai/12-02-3/2015	H3N2	Negative
EPI_ISL_252709	A/swine/Iowa/A01776537/2016	H3N2	Negative
EPI_ISL_256260	A/swine/Indiana/A01812059/2015	H3N2	Negative
EPI_ISL_195190	A/swine/Belgium/Izegem-538/2010	H3N2	Negative
EPI_ISL_24589	A/swine/Korea/CY09/2007	H3N2	Negative
EPI_ISL_116394	A/swine/Illinois/A01047014/2010	H3N2	Negative
EPI_ISL_142019	A/swine/Wyoming/A01379327/2013	H3N2	Negative
EPI_ISL_236810	A/swine/Indiana/A01812151/2016	H3N2	Negative
EPI_ISL_142643	A/Swine/Spain/80598LP1/2007	H3N2	Negative
EPI_ISL_29102	A/swine/Bissendorf/IDT1864/03	H3N2	Negative
EPI_ISL_104805	A/swine/North_Carolina/A01076204/2010	H3N2	Negative
EPI_ISL_156335	A/swine/Indiana/A00968359/2012	H3N2	Negative
EPI_ISL_116350	A/swine/Iowa/A01076630/2010	H3N2	Negative
EPI_ISL_161143	A/swine/Ohio/13TOSU2052/2013	H3N2	Negative
EPI_ISL_278821	A/swine/Korea/S2001/2015	H3N2	Negative
EPI_ISL_207050	A/swine/Manitoba/D0102/2012	H3N2	Negative
EPI_ISL_93359	A/swine/Minnesota/A01049346/2010	H3N2	Negative
EPI_ISL_207875	A/swine/Saskatchewan/SD0025/2014	H3N2	Negative
EPI_ISL_164477	A/swine/DongNai/10-12-1/2013	H3N2	Negative
EPI_ISL_261508	A/swine/Illinois/16TOSU7168/2016	H3N2	Negative
EPI_ISL_278437	A/swine/Oklahoma/A02218159/2017	H3N2	Negative
EPI_ISL_133356	A/swine/Germany/R2807/2009	H3N2	Negative
EPI_ISL_257035	A/swine/Indiana/A01672517/2017	H3N2	Negative
EPI_ISL_29806	A/swine/Minnesota/578/2007	H3N2	Negative
EPI_ISL_151252	A/swine/South_Dakota/SG1453/2003	H3N2	Negative
EPI_ISL_218289	A/swine/Dong_Nai/08-20-1/2015	H3N2	Negative
EPI_ISL_127707	A/swine/Indiana/A01203372/2012	H3N2	Negative
EPI_ISL_180673	A/swine/Missouri/A01840324/2015	H3N2	Negative
EPI_ISL_207170	A/swine/Saskatchewan/SD0012/2013	H3N2	Negative
EPI_ISL_124340	A/swine/Illinois/A01076724/2010	H3N2	Negative
EPI_ISL_282460	A/swine/Manitoba/D0471/2016	H3N2	Negative
EPI_ISL_195191	A/swine/Belgium/Valdegem-581/2010	H3N2	Negative
EPI_ISL_124402	A/swine/Iowa/A01076508/2010	H3N2	Negative
EPI_ISL_142215	A/swine/Korea/KSB/2012	H3N2	Negative
EPI_ISL_271043	A/swine/Iowa/MT_12_07_1931/2012	H3N2	Negative
EPI_ISL_278922	A/swine/Italy/171855/2011	H3N2	Negative
EPI_ISL_211812	A/swine/Illinois/A01728585/2015	H3N2	Negative
EPI_ISL_282692	A/swine/Alberta/SD0123/2015	H3N2	Negative
EPI_ISL_278844	A/swine/Italy/1103/2015	H3N2	Negative
EPI_ISL_133680	A/swine/Iowa/A01277915/2012	H3N2	Negative
EPI_ISL_282481	A/swine/Alberta/SD0186/2016	H3N2	Negative
EPI_ISL_272738	A/swine/Kansas/A01378025/2017	H3N2	Negative
EPI_ISL_222495	A/swine/Illinois/A01894510/2016	H3N2	Negative
EPI_ISL_282713	A/swine/Saskatchewan/SD0100/2015	H3N2	Negative
EPI_ISL_134279	A/swine/Michigan/A01259002/2012	H3N2	Negative
EPI_ISL_161427	A/swine/Bac_Ninh/118/2012	H3N2	Negative
EPI_ISL_235750	A/swine/North_Carolina/A01732564/2016	H3N2	Negative
EPI_ISL_297066	A/swine/Iowa/A02134994/2017	H3N2	Negative
EPI_ISL_13217	A/Swine/Shandong/3/2005/	H3N2	Negative
EPI_ISL_134301	A/swine/North_Carolina/A01442548/2012	H3N2	Negative

EPI_ISL_130016	A/swine/Illinois/A01327868/2012	H3N2	Negative
EPI_ISL_218291	A/swine/Dong_Nai/08-22-1/2015	H3N2	Negative
EPI_ISL_124404	A/swine/Minnesota/A01076531/2010	H3N2	Negative
EPI_ISL_281244	A/swine/Saskatchewan/SD0069/2015	H3N2	Negative
EPI_ISL_130053	A/swine/Illinois/A01241208/2012	H3N2	Negative
EPI_ISL_282405	A/swine/Iowa/A02219788/2017	H3N2	Negative
EPI_ISL_161429	A/swine/Bac_Ninh/144/2012	H3N2	Negative
EPI_ISL_90102	A/swine/Iowa/A01049034/2010	H3N2	Negative
EPI_ISL_136652	A/swine/Maryland/A01444460/2013	H3N2	Negative
EPI_ISL_296676	A/swine/North_Carolina/A02223299/2017	H3N2	Negative
EPI_ISL_161426	A/swine/Bac_Ninh/116/2012	H3N2	Negative
EPI_ISL_282459	A/swine/Alberta/SD0197/2016	H3N2	Negative
EPI_ISL_282479	A/swine/Manitoba/D0459/2016	H3N2	Negative
EPI_ISL_234530	A/swine/North_Carolina/A01777989/2016	H3N2	Negative
EPI_ISL_297150	A/swine/Ohio/16TOSU3157/2016	H3N2	Negative
EPI_ISL_213796	A/swine/Iowa/A01729543/2016	H3N2	Negative
EPI_ISL_282400	A/swine/North_Carolina/A02219753/2017	H3N2	Negative
EPI_ISL_96932	A/swine/Minnesota/001444/2007	H3N2	Negative
EPI_ISL_129533	A/swine/Spain/82108/2007	H3N2	Negative
EPI_ISL_13923	A/swine/Nakhon_pathom/NIAH586-1/2005	H3N2	Negative
EPI_ISL_206373	A/swine/Indiana/A01812022/2015	H3N2	Negative
EPI_ISL_195221	A/swine/Spain/31768/2012	H3N2	Negative
EPI_ISL_116356	A/swine/Nebraska/A01116980/2011	H3N2	Negative
EPI_ISL_160794	A/swine/DongNai/07-17-1/2012	H3N2	Negative
EPI_ISL_180672	A/swine/Illinois/A01840351/2015	H3N2	Negative
EPI_ISL_133354	A/swine/Germany/R325/2009	H3N2	Negative
EPI_ISL_195192	A/swine/Netherlands/Roosendaal-571/2011	H3N2	Negative
EPI_ISL_207045	A/swine/Manitoba/D0098/2012	H3N2	Negative
EPI_ISL_161152	A/swine/Indiana/13TOSU1207/2013	H3N2	Negative
EPI_ISL_124310	A/swine/Iowa/A01202657/2011	H3N2	Negative
EPI_ISL_262591	A/swine/Iowa/A01667095/2017	H3N2	Negative
EPI_ISL_271725	A/swine/Minnesota/MT_12_07_11_3_27/2011	H3N2	Negative
EPI_ISL_144533	A/swine/Illinois/A01271617/2012	H3N2	Negative
EPI_ISL_174302	A/swine/Arkansas/A01840027/2014	H3N2	Negative
EPI_ISL_130131	A/swine/Iowa/A01300194/2012	H3N2	Negative
EPI_ISL_195193	A/swine/Belgium/Moorslede-515/2011	H3N2	Negative
EPI_ISL_136563	A/swine/Michigan/A01432375/2013	H3N2	Negative
EPI_ISL_67909	A/swine/Guangdong/111/2002	H3N2	Negative
EPI_ISL_282729	A/swine/Saskatchewan/SD0097/2015	H3N2	Negative
EPI_ISL_222484	A/swine/North_Carolina/A01894277/2016	H3N2	Negative
EPI_ISL_195217	A/swine/Belgium/Belsele-66/2013	H3N2	Negative
EPI_ISL_157605	A/swine/Iowa/13G060/2013	H3N2	Negative
EPI_ISL_278856	A/swine/Italy/341031-3/2014	H3N2	Negative
EPI_ISL_151290	A/swine/North_Carolina/01216/2006	H3N2	Negative
EPI_ISL_280163	A/swine/Kansas/A01378028/2017	H3N2	Negative
EPI_ISL_151619	A/swine/Missouri/00355/2004	H3N2	Negative
EPI_ISL_234244	A/swine/Indiana/A01812202/2016	H3N2	Negative
EPI_ISL_24584	A/swine/Korea/CAS07/2005	H3N2	Negative
EPI_ISL_271662	A/swine/Iowa/MT_12_07_4307/2012	H3N2	Negative
EPI_ISL_195185	A/swine/Germany/Stadtloder-IDT11945/2010	H3N2	Negative
EPI_ISL_132356	A/swine/Iowa/A01203943/2012	H3N2	Negative
EPI_ISL_281252	A/swine/Saskatchewan/SD0026/2014	H3N2	Negative
EPI_ISL_161186	A/swine/Ohio/13TOSU2132/2013	H3N2	Negative
EPI_ISL_281954	A/swine/Minnesota/MT_13_01_S63/2013	H3N2	Negative
EPI_ISL_166548	A/swine/Nebraska/A01476165/2014	H3N2	Negative
EPI_ISL_252781	A/swine/Minnesota/A01668936/2016	H3N2	Negative
EPI_ISL_198367	A/swine/Nebraska/A02034991/2015	H3N2	Negative
EPI_ISL_102531	A/swine/Iowa/A01202659/2011	H3N2	Negative

EPI_ISL_211815	A/swine/Illinois/A01728293/2015	H3N2	Negative
EPI_ISL_163049	A/swine/DongNai/07-08-2/2013	H3N2	Negative
EPI_ISL_195184	A/swine/Germany/Ottendorf-IDT11910/2010	H3N2	Negative
EPI_ISL_151640	A/swine/Alabama/SG1224/2005	H3N2	Negative
EPI_ISL_245065	A/swine/North_Dakota/A01782435/2016	H3N2	Negative
EPI_ISL_284679	A/swine/Texas/A01785299/2017	H3N2	Negative
EPI_ISL_282707	A/swine/Alberta/SD0109/2015	H3N2	Negative
EPI_ISL_13899	A/swine/Guangdong/166/06	H3N2	Negative
EPI_ISL_243373	A/swine/North_Carolina/A01781199/2016	H3N2	Negative
EPI_ISL_260821	A/swine/Iowa/A01932578/2017	H3N2	Negative
EPI_ISL_195201	A/swine/Spain/29403/2012	H3N2	Negative
EPI_ISL_296261	A/swine/North_Carolina/A02135886/2017	H3N2	Negative
EPI_ISL_216893	A/swine/North_Carolina/A01730588/2016	H3N2	Negative
EPI_ISL_282731	A/swine/Saskatchewan/SD0067/2015	H3N2	Negative
EPI_ISL_150829	A/swine/Oklahoma/SG1216/2005	H3N2	Negative
EPI_ISL_208970	A/swine/North_Carolina/A01797711/2015	H3N2	Negative
EPI_ISL_218292	A/swine/Dong_Nai/08-25-1/2015	H3N2	Negative
EPI_ISL_235742	A/swine/North_Carolina/A01732322/2016	H3N2	Negative
EPI_ISL_161151	A/swine/Indiana/13TOSU1217/2013	H3N2	Negative
EPI_ISL_282454	A/swine/Alberta/SD0208/2016	H3N2	Negative
EPI_ISL_237042	A/swine/Nebraska/A01781225/2016	H3N2	Negative
EPI_ISL_234572	A/swine/Iowa/A01732489/2016	H3N2	Negative
EPI_ISL_243756	A/swine/Ohio/14TOSU0294/2014	H3N2	Negative
EPI_ISL_129504	A/swine/England/87842/1990	H3N2	Negative
EPI_ISL_130130	A/swine/Iowa/A01300451/2012	H3N2	Negative
EPI_ISL_278810	A/swine/Quebec/1708732/2015	H3N2	Negative
EPI_ISL_205064	A/swine/Illinois/A01944946/2015	H3N2	Negative
EPI_ISL_116359	A/swine/Pennsylvania/A01201666/2011	H3N2	Negative
EPI_ISL_195219	A/swine/Spain/33936/2012	H3N2	Negative
EPI_ISL_207966	A/swine/Saskatchewan/SD0041/2014	H3N2	Negative
EPI_ISL_282691	A/swine/British_Columbia/SD0122/2015	H3N2	Negative
EPI_ISL_161668	A/swine/DongNai/07-29-1/2012	H3N2	Negative
EPI_ISL_203176	A/swine/Iowa/A01944136/2015	H3N2	Negative
EPI_ISL_63633	A/swine/Oklahoma/011506/2007	H3N2	Negative
EPI_ISL_234956	A/swine/North_Carolina/A01777729/2016	H3N2	Negative
EPI_ISL_195215	A/swine/Germany/Coesfeld-Harle-IDT14741-1/2012	H3N2	Negative
EPI_ISL_24586	A/swine/Korea/CY04/2007	H3N2	Negative
EPI_ISL_200878	A/swine/Kentucky/14TOSU23816/2014	H3N2	Negative
EPI_ISL_282464	A/swine/Manitoba/D0475/2016	H3N2	Negative
EPI_ISL_168340	A/swine/Indiana/13TOSU5095/2013	H3N2	Negative
EPI_ISL_29805	A/swine/Minnesota/66960/2006	H3N2	Negative
EPI_ISL_209376	A/swine/Iowa/A01727249/2015	H3N2	Negative
EPI_ISL_225508	A/swine/North_Carolina/A01895828/2016	H3N2	Negative
EPI_ISL_129523	A/swine/Italy/15360-3/2001	H3N2	Negative
EPI_ISL_282473	A/swine/Manitoba/D0467/2016	H3N2	Negative
EPI_ISL_5141	A/swine/Spain/33601/2001	H3N2	Negative
EPI_ISL_124343	A/swine/Minnesota/A01201424/2010	H3N2	Negative
EPI_ISL_207967	A/swine/Manitoba/D0354/2014	H3N2	Negative
EPI_ISL_168507	A/swine/Kansas/A01377371/2014	H3N2	Negative
EPI_ISL_155696	A/swine/Nebraska/A01409533/2013	H3N2	Negative
EPI_ISL_230187	A/swine/Missouri/A01775853/2016	H3N2	Negative
EPI_ISL_130179	A/swine/South_Dakota/A01300602/2012	H3N2	Negative
EPI_ISL_134289	A/swine/Illinois/A01244402/2012	H3N2	Negative
EPI_ISL_282724	A/swine/Manitoba/SD0085/2015	H3N2	Negative
EPI_ISL_138597	A/swine/HuNan/01/2008	H3N2	Negative
EPI_ISL_203185	A/swine/Ohio/A01944458/2015	H3N2	Negative
EPI_ISL_271646	A/swine/Iowa/MT_12_07_1912/2012	H3N2	Negative
EPI_ISL_143878	A/swine/Wyoming/A01381213/2013	H3N2	Negative

EPI_ISL_128163	A/swine/Mexico/Mex51/2010	H3N2	Negative
EPI_ISL_218611	A/swine/Dong_Nai/12-14-3/2015	H3N2	Negative
EPI_ISL_129520	A/swine/Gent/96/2007	H3N2	Negative
EPI_ISL_128940	A/Swine/Spain/SF32071/2007	H3N2	Negative
EPI_ISL_282470	A/swine/Manitoba/D0464/2016	H3N2	Negative
EPI_ISL_278799	A/swine/Quebec/1698182/2014	H3N2	Negative
EPI_ISL_151212	A/swine/Mexico/SG1444/2011	H3N2	Negative
EPI_ISL_278848	A/swine/Italy/23398/2015	H3N2	Negative
EPI_ISL_218052	A/swine/Dong_Nai/09-15-1/2014	H3N2	Negative
EPI_ISL_208655	A/Swine/Cambodia/TangKrang/060301/2011/H3N2	H3N2	Negative
EPI_ISL_188905	A/swine/South_Dakota/A01847612/2015	H3N2	Negative
EPI_ISL_329654	A/swine/Ohio/OH-18-6906/2018	H3N2	Negative
EPI_ISL_195182	A/swine/Belgium/Gent-131/2000	H3N2	Negative
EPI_ISL_271738	A/swine/Iowa/MT_12_07_3836/2012	H3N2	Negative
EPI_ISL_218569	A/swine/Bac_Ninh/43-20-3/2015	H3N2	Negative
EPI_ISL_151350	A/swine/Texas/SG1501/2010	H3N2	Negative
EPI_ISL_282723	A/swine/Manitoba/SD0084/2015	H3N2	Negative
EPI_ISL_124437	A/swine/Illinois/A01116190/2011	H3N2	Negative
EPI_ISL_134284	A/swine/Iowa/A01432207/2012	H3N2	Negative
EPI_ISL_207035	A/swine/Manitoba/D0092/2012	H3N2	Negative
EPI_ISL_207984	A/swine/Saskatchewan/SD0065/2014	H3N2	Negative
EPI_ISL_148312	A/swine/Virginia/A01290329/2013	H3N2	Negative
EPI_ISL_130117	A/swine/Nebraska/A01300022/2012	H3N2	Negative
EPI_ISL_207038	A/swine/Manitoba/D0095/2012	H3N2	Negative
EPI_ISL_216894	A/swine/Indiana/A01730580/2016	H3N2	Negative
EPI_ISL_151253	A/swine/Nebraska/00188/2003	H3N2	Negative
EPI_ISL_129506	A/swine/England/90591/1997	H3N2	Negative
EPI_ISL_207049	A/swine/Manitoba/D0100/2012	H3N2	Negative
EPI_ISL_278869	A/swine/Italy/134414/2014	H3N2	Negative
EPI_ISL_218606	A/swine/Dong_Nai/12-09-3/2015	H3N2	Negative
EPI_ISL_207057	A/swine/Manitoba/D0106/2012	H3N2	Negative
EPI_ISL_280197	A/swine/Indiana/16TOSU4637/2016	H3N2	Negative
EPI_ISL_109922	A/swine/Iowa/A01202088/2011	H3N2	Negative
EPI_ISL_207964	A/swine/Alberta/SD0038/2014	H3N2	Negative
EPI_ISL_151209	A/swine/Saskatchewan/SD0001/2011	H3N2	Negative
EPI_ISL_127727	A/swine/Iowa/A01243311/2012	H3N2	Negative
EPI_ISL_280242	A/swine/Kentucky/16TOSU5910/2016	H3N2	Negative
EPI_ISL_129582	A/swine/Wisconsin/H02AS8/2002	H3N2	Negative
EPI_ISL_207059	A/swine/Manitoba/D0177/2012	H3N2	Negative
EPI_ISL_218607	A/swine/Dong_Nai/12-10-3/2015	H3N2	Negative
EPI_ISL_188904	A/swine/Missouri/A01847626/2015	H3N2	Negative
EPI_ISL_151273	A/swine/North_Carolina/01052/2006	H3N2	Negative
EPI_ISL_151636	A/swine/North_Carolina/00116/2003	H3N2	Negative
EPI_ISL_282715	A/swine/Alberta/SD0101/2015	H3N2	Negative
EPI_ISL_127730	A/swine/Iowa/A01049119/2010	H3N2	Negative
EPI_ISL_329655	A/swine/Ohio/OH-18-6910/2018	H3N2	Negative
EPI_ISL_151287	A/swine/Texas/00816/2005	H3N2	Negative
EPI_ISL_84915	A/swine/Italy/6/1981	H3N2	Negative
EPI_ISL_271644	A/swine/Minnesota/MT_12_07_540/2012	H3N2	Negative
EPI_ISL_222527	A/swine/Illinois/A01895671/2016	H3N2	Negative
EPI_ISL_151272	A/swine/North_Carolina/01506/2006	H3N2	Negative
EPI_ISL_148108	A/swine/Guangdong/L23/2010	H3N2	Negative
EPI_ISL_102544	A/swine/Iowa/A01202709/2011	H3N2	Negative
EPI_ISL_179622	A/swine/Missouri/A01554378/2015	H3N2	Negative
EPI_ISL_6908	A/swine/Spain/42386/2002	H3N2	Negative
EPI_ISL_150841	A/swine/North_Carolina/01312/2006	H3N2	Negative
EPI_ISL_245076	A/swine/Iowa/A01782576/2016	H3N2	Negative
EPI_ISL_207920	A/swine/Manitoba/D0340/2014	H3N2	Negative

EPI_ISL_200086	A/swine/Oklahoma/A01770191/2015	H3N2	Negative
EPI_ISL_207143	A/swine/Manitoba/D0281/2013	H3N2	Negative
EPI_ISL_271639	A/swine/Minnesota/MT_12_07_11_3_4/2011	H3N2	Negative
EPI_ISL_282720	A/swine/Manitoba/D0430/2015	H3N2	Negative
EPI_ISL_117563	A/swine/Illinois/A00857304b/2012	H3N2	Negative
EPI_ISL_237047	A/swine/Minnesota/A01781044/2016	H3N2	Negative
EPI_ISL_278797	A/swine/Quebec/1561816/2014	H3N2	Negative
EPI_ISL_167970	A/swine/Nebraska/A01565757/2014	H3N2	Negative
EPI_ISL_218609	A/swine/Dong_Nai/12-12-3/2015	H3N2	Negative
EPI_ISL_218050	A/swine/Dong_Nai/09-13-1/2014	H3N2	Negative
EPI_ISL_271736	A/swine/Minnesota/MT_12_07_1654/2012	H3N2	Negative
EPI_ISL_161641	A/swine/Bac_Ninh/155/2012	H3N2	Negative
EPI_ISL_124046	A/swine/Michigan/A01203498/2012	H3N2	Negative
EPI_ISL_97111	A/swine/Nebraska/A01049937/2011	H3N2	Negative
EPI_ISL_282545	A/swine/Saskatchewan/SD0149/2016	H3N2	Negative
EPI_ISL_195994	A/swine/Mexico/8630459/2011	H3N2	Negative
EPI_ISL_205067	A/swine/Iowa/A01795793/2015	H3N2	Negative
EPI_ISL_195198	A/swine/Spain/28778/2012	H3N2	Negative
EPI_ISL_90366	A/swine/Iowa/A01049160/2010	H3N2	Negative
EPI_ISL_208657	A/Swine/Cambodia/Chranieng/200503/2012/H3N2	H3N2	Negative
EPI_ISL_129481	A/swine/England/375017/1993	H3N2	Negative
EPI_ISL_84917	A/swine/Brabant/1984	H3N2	Negative
EPI_ISL_229216	A/swine/North_Carolina/A01776152/2016	H3N2	Negative
EPI_ISL_151604	A/swine/Texas/00226/2003	H3N2	Negative
EPI_ISL_278887	A/swine/Italy/352498/2013	H3N2	Negative
EPI_ISL_278824	A/swine/Italy/229049/2015	H3N2	Negative
EPI_ISL_271083	A/swine/South_Dakota/MT_12_07_1566/2012	H3N2	Negative
EPI_ISL_133006	A/swine/Minnesota/A01271731/2012	H3N2	Negative
EPI_ISL_278803	A/swine/Quebec/1626561/2014	H3N2	Negative
EPI_ISL_203105	A/swine/North_Carolina/A01945726/2015	H3N2	Negative
EPI_ISL_282491	A/swine/Alberta/SD0181/2016	H3N2	Negative
EPI_ISL_96934	A/swine/Minnesota/001332/2006	H3N2	Negative
EPI_ISL_280240	A/swine/Indiana/16TOSU7610/2016	H3N2	Negative
EPI_ISL_109876	A/swine/Minnesota/A01047396/2011	H3N2	Negative
EPI_ISL_207969	A/swine/Saskatchewan/SD0045/2014	H3N2	Negative
EPI_ISL_218605	A/swine/Dong_Nai/12-08-3/2015	H3N2	Negative
EPI_ISL_207145	A/swine/Manitoba/D0283/2013	H3N2	Negative
EPI_ISL_151608	A/swine/Oklahoma/00141/2003	H3N2	Negative
EPI_ISL_133484	A/swine/Germany/R655/2012	H3N2	Negative
EPI_ISL_124246	A/swine/Illinois/A01240578/2011	H3N2	Negative
EPI_ISL_10148	A/swine/Manitoba/12707/2005	H3N2	Negative
EPI_ISL_282520	A/swine/Manitoba/SD0151/2016	H3N2	Negative
EPI_ISL_246038	A/swine/North_Carolina/A01668240/2016	H3N2	Negative
EPI_ISL_218044	A/swine/Dong_Nai/09-02-1/2014	H3N2	Negative
EPI_ISL_191504	A/swine/Iowa/A02077646/2015	H3N2	Negative
EPI_ISL_88842	A/swine/Minnesota/239105/2009	H3N2	Negative
EPI_ISL_162562	A/swine/Nebraska/A01493915/2014	H3N2	Negative
EPI_ISL_207072	A/swine/Manitoba/D0083/2013	H3N2	Negative
EPI_ISL_161434	A/swine/Bac_Ninh/150/2012	H3N2	Negative
EPI_ISL_151613	A/swine/Nebraska/SG1178/2003	H3N2	Negative
EPI_ISL_13896	A/swine/Heilongjiang/1/05	H3N2	Negative
EPI_ISL_13925	A/swine/Ratchaburi/NIAH874/2005	H3N2	Negative
EPI_ISL_24582	A/swine/Korea/CAS05/2004	H3N2	Negative
EPI_ISL_130038	A/swine/Minnesota/A01327764/2012	H3N2	Negative
EPI_ISL_207116	A/swine/Manitoba/D0271/2013	H3N2	Negative
EPI_ISL_218241	A/swine/Bac_Ninh/251/2015	H3N2	Negative
EPI_ISL_133360	A/swine/Germany/R494/2010	H3N2	Negative
EPI_ISL_109911	A/swine/Iowa/A01049033/2010	H3N2	Negative

EPI_ISL_218286	A/swine/Dong_Nai/08-16-1/2015	H3N2	Negative
EPI_ISL_150942	A/swine/Texas/03042/2010	H3N2	Negative
EPI_ISL_271058	A/swine/South_Dakota/MT_12_07_2008/2012	H3N2	Negative
EPI_ISL_282735	A/swine/Alberta/SD0079/2015	H3N2	Negative
EPI_ISL_174304	A/swine/Arkansas/A01555074/2014	H3N2	Negative
EPI_ISL_208981	A/swine/Ohio/A01796509/2015	H3N2	Negative
EPI_ISL_133903	A/swine/Ohio/11SW170/2011	H3N2	Negative
EPI_ISL_195211	A/swine/Belgium/Glabbeek-284/2012	H3N2	Negative
EPI_ISL_278913	A/swine/Italy/337155/2012	H3N2	Negative
EPI_ISL_237045	A/swine/Minnesota/A01781222/2016	H3N2	Negative
EPI_ISL_200089	A/swine/North_Carolina/A01770073/2015	H3N2	Negative
EPI_ISL_282447	A/swine/Manitoba/D0476/2016	H3N2	Negative
EPI_ISL_207071	A/swine/Manitoba/D0195/2013	H3N2	Negative
EPI_ISL_271055	A/swine/Minnesota/MT_12_07_1647/2012	H3N2	Negative
EPI_ISL_282700	A/swine/Saskatchewan/SD0118/2015	H3N2	Negative
EPI_ISL_180661	A/swine/Nebraska/A01567868/2015	H3N2	Negative
EPI_ISL_28430	A/swine/Udon_Thani/NIAH464/2004	H3N2	Negative
EPI_ISL_195195	A/swine/Netherlands/Vragender-503/2011	H3N2	Negative
EPI_ISL_151020	A/swine/Saskatchewan/02903/2009	H3N2	Negative
EPI_ISL_296547	A/swine/Ohio/A02134225/2017	H3N2	Negative
EPI_ISL_225563	A/swine/North_Carolina/A01894904/2016	H3N2	Negative
EPI_ISL_226067	A/swine/Colorado/A01775092/2016	H3N2	Negative
EPI_ISL_282696	A/swine/Manitoba/SD0131/2015	H3N2	Negative
EPI_ISL_235739	A/swine/Minnesota/A01942325/2016	H3N2	Negative
EPI_ISL_328758	A/swine/Indiana/17TOSU1833/2017	H3N2	Negative
EPI_ISL_124472	A/swine/Nebraska/A01116984/2011	H3N2	Negative
EPI_ISL_278875	A/swine/Italy/91602/2014	H1N2	Negative
EPI_ISL_74387	A/swine/Hong_Kong/NS1889/2009	H1N2	Negative
EPI_ISL_133788	A/swine/Ohio/09SW1478M/2009	H1N2	Negative
EPI_ISL_207968	A/swine/Alberta/SD0042/2014	H1N2	Negative
EPI_ISL_203138	A/swine/Illinois/A01944807/2015	H1N2	Negative
EPI_ISL_119366	A/swine/Minnesota/A01201429/2011	H1N2	Negative
EPI_ISL_137561	A/swine/Missouri/A01444795/2013	H1N2	Negative
EPI_ISL_211799	A/swine/Oklahoma/A01728097/2015	H1N2	Negative
EPI_ISL_297193	A/swine/Indiana/A02136747/2017	H1N2	Negative
EPI_ISL_29767	A/swine/Hong_Kong/NS623/2002	H1N2	Negative
EPI_ISL_29770	A/swine/Hong_Kong/1110/2006	H1N2	Negative
EPI_ISL_208920	A/swine/North_Carolina/A01796370/2015	H1N2	Negative
EPI_ISL_88360	A/swine/Hong_Kong/1111/2004	H1N2	Negative
EPI_ISL_142741	A/swine/Korea/CY0423-33/2013	H1N2	Negative
EPI_ISL_280233	A/swine/Indiana/16TOSU8165/2016	H1N2	Negative
EPI_ISL_253710	A/swine/Iowa/A01671477/2016	H1N2	Negative
EPI_ISL_218582	A/swine/Dong_Nai/07-05-3/2015	H1N2	Negative
EPI_ISL_97075	A/swine/Iowa/A01049723/2011	H1N2	Negative
EPI_ISL_151193	A/swine/Oklahoma/01117/2006	H1N2	Negative
EPI_ISL_205043	A/swine/North_Carolina/A01945949/2015	H1N2	Negative
EPI_ISL_261488	A/swine/Iowa/A01932577/2017	H1N2	Negative
EPI_ISL_88840	A/swine/North_Carolina/226126/2010	H1N2	Negative
EPI_ISL_222687	A/swine/Indiana/A01894974/2016	H1N2	Negative
EPI_ISL_130144	A/swine/Illinois/A01328923/2012	H1N2	Negative
EPI_ISL_96982	A/swine/Guangdong/2676/2010	H1N2	Negative
EPI_ISL_157196	A/swine/Missouri/A01411322/2014	H1N2	Negative
EPI_ISL_119350	A/swine/Minnesota/A01134353/2011	H1N2	Negative
EPI_ISL_212193	A/swine/Nebraska/A01941775/2016	H1N2	Negative
EPI_ISL_151278	A/swine/Arkansas/02927/2009	H1N2	Negative
EPI_ISL_278895	A/swine/Italy/87434/2014	H1N2	Negative
EPI_ISL_150839	A/swine/Texas/SG1250/2006	H1N2	Negative
EPI_ISL_150985	A/swine/North_Carolina/SG1365/2010	H1N2	Negative

EPI_ISL_219062	A/swine/Dong_Nai/07-23-3/2015	H1N2	Negative
EPI_ISL_246032	A/swine/Iowa/A01668210/2016	H1N2	Negative
EPI_ISL_13920	A/swine/Saraburi/NIAH13021/2005	H1N2	Negative
EPI_ISL_282453	A/swine/Manitoba/SD0209/2016	H1N2	Negative
EPI_ISL_124488	A/swine/Illinois/A01134580/2011	H1N2	Negative
EPI_ISL_282523	A/swine/Saskatchewan/SD0155/2016	H1N2	Negative
EPI_ISL_218284	A/swine/Dong_Nai/07-27-1/2015	H1N2	Negative
EPI_ISL_211825	A/swine/North_Carolina/A01728820/2015	H1N2	Negative
EPI_ISL_194997	A/swine/Germany/Steinfeld-IDT12115/2010	H1N2	Negative
EPI_ISL_180662	A/swine/Minnesota/A01567956/2014	H1N2	Negative
EPI_ISL_195100	A/swine/Denmark/10345-1/2012	H1N2	Negative
EPI_ISL_258654	A/swine/South_Carolina/A01932046/2017	H1N2	Negative
EPI_ISL_119337	A/swine/Minnesota/A01076209/2010	H1N2	Negative
EPI_ISL_130123	A/swine/Nebraska/A01300150/2012	H1N2	Negative
EPI_ISL_88355	A/swine/Hong_Kong/NS30/2004	H1N2	Negative
EPI_ISL_200093	A/swine/North_Carolina/A02076945/2015	H1N2	Negative
EPI_ISL_9882	A/swine/Ontario/48235/04	H1N2	Negative
EPI_ISL_160437	A/swine/DongNai/03-03-2/2011	H1N2	Negative
EPI_ISL_278899	A/swine/Italy/327669/2013	H1N2	Negative
EPI_ISL_79311	A/swine/Germany-NI/R757/10	H1N2	Negative
EPI_ISL_217471	A/swine/Illinois/A01731417/2016	H1N2	Negative
EPI_ISL_207233	A/swine/Italy/9529-70/2013	H1N2	Negative
EPI_ISL_95878	A/swine/Italy/18/2000	H1N2	Negative
EPI_ISL_234287	A/swine/North_Carolina/A01777394/2016	H1N2	Negative
EPI_ISL_218592	A/swine/Dong_Nai/07-21-3/2015	H1N2	Negative
EPI_ISL_234240	A/swine/Indiana/A01812236/2016	H1N2	Negative
EPI_ISL_11923	A/swine/Guangxi/13/2006	H1N2	Negative
EPI_ISL_262594	A/swine/Pennsylvania/A01933903/2017	H1N2	Negative
EPI_ISL_100195	A/swine/Ohio/FAH10-1/2010	H1N2	Negative
EPI_ISL_151294	A/swine/Oklahoma/01382/2006	H1N2	Negative
EPI_ISL_195459	A/swine/Missouri/A02076915/2015	H1N2	Negative
EPI_ISL_95863	A/swine/Italy/4675/2003	H1N2	Negative
EPI_ISL_328824	A/swine/Chile/39/2014	H1N2	Negative
EPI_ISL_207232	A/swine/Italy/9529-1/2013	H1N2	Negative
EPI_ISL_124394	A/swine/North_Carolina/A01076202/2010	H1N2	Negative
EPI_ISL_218593	A/swine/Dong_Nai/07-22-3/2015	H1N2	Negative
EPI_ISL_245062	A/swine/South_Carolina/A01782474/2016	H1N2	Negative
EPI_ISL_256560	A/swine/Iowa/A01672419/2017	H1N2	Negative
EPI_ISL_253645	A/swine/Michigan/A01671231/2016	H1N2	Negative
EPI_ISL_4666	A/swine/Bakum/1832/2000	H1N2	Negative
EPI_ISL_74371	A/swine/Hong_Kong/1435/2009	H1N2	Negative
EPI_ISL_234579	A/swine/Indiana/A01778367/2016	H1N2	Negative
EPI_ISL_195069	A/swine/France/CotesdArmor-0074/2011	H1N2	Negative
EPI_ISL_222696	A/swine/Illinois/A01895418/2016	H1N2	Negative
EPI_ISL_29772	A/swine/Hong_Kong/915/2004	H1N2	Negative
EPI_ISL_195057	A/swine/Spain/24237/2011	H1N2	Negative
EPI_ISL_195114	A/swine/England/463180/1994	H1N2	Negative
EPI_ISL_218057	A/swine/Tien_Giang/08-12-1/2014	H1N2	Negative
EPI_ISL_243775	A/swine/Indiana/15TOSU4060/2015	H1N2	Negative
EPI_ISL_212200	A/swine/Iowa/A01729216/2016	H1N2	Negative
EPI_ISL_236808	A/swine/North_Carolina/A01732706/2016	H1N2	Negative
EPI_ISL_285337	A/swine/Missouri/A02221494/2017	H1N2	Negative
EPI_ISL_234951	A/swine/Iowa/A01777667/2016	H1N2	Negative
EPI_ISL_174440	A/swine/Minnesota/A01567012/2014	H1N2	Negative
EPI_ISL_282406	A/swine/Iowa/A02219785/2017	H1N2	Negative
EPI_ISL_243357	A/swine/Iowa/A01781147/2016	H1N2	Negative
EPI_ISL_136651	A/swine/Illinois/A01444513/2013	H1N2	Negative
EPI_ISL_129508	A/swine/England/P185/2008	H1N2	Negative

EPI_ISL_285479	A/swine/Oklahoma/A02221360/2017	H1N2	Negative
EPI_ISL_263483	A/swine/Indiana/16TOSU0646/2016	H1N2	Negative
EPI_ISL_218283	A/swine/Dong_Nai/07-26-1/2015	H1N2	Negative
EPI_ISL_129452	A/swine/England/002295/2002	H1N2	Negative
EPI_ISL_29768	A/swine/Hong_Kong/78/2003	H1N2	Negative
EPI_ISL_161119	A/swine/Texas/13TOSU6276/2013	H1N2	Negative
EPI_ISL_278858	A/swine/Italy/352331/2014	H1N2	Negative
EPI_ISL_129530	A/swine/Ploufragan/0214/2006	H1N2	Negative
EPI_ISL_281988	A/swine/Oklahoma/A02219505/2017	H1N2	Negative
EPI_ISL_129489	A/swine/England/448813/1994	H1N2	Negative
EPI_ISL_63211	A/swine/Texas/050593/2008	H1N2	Negative
EPI_ISL_278923	A/swine/Italy/229537/2011	H1N2	Negative
EPI_ISL_203097	A/swine/Iowa/A01795483/2015	H1N2	Negative
EPI_ISL_271726	A/swine/Minnesota/MT_12_07_2130/2012	H1N2	Negative
EPI_ISL_129513	A/swine/Cotes_d'Armor/1174/2009	H1N2	Negative
EPI_ISL_170574	A/swine/North_Carolina/A01476787/2014	H1N2	Negative
EPI_ISL_150990	A/swine/Arkansas/SG1377/2011	H1N2	Negative
EPI_ISL_235732	A/swine/Illinois/A01778711/2016	H1N2	Negative
EPI_ISL_222483	A/swine/North_Carolina/A01894401/2016	H1N2	Negative
EPI_ISL_164462	A/swine/DongNai/03-17-2/2011	H1N2	Negative
EPI_ISL_119368	A/swine/North_Carolina/A01076192/2010	H1N2	Negative
EPI_ISL_161136	A/swine/Ohio/13TOSU0558/2013	H1N2	Negative
EPI_ISL_129493	A/swine/England/60797/2000	H1N2	Negative
EPI_ISL_278854	A/swine/Italy/334694-1/2014	H1N2	Negative
EPI_ISL_328785	A/swine/Rancagua/VN1401-143/2014	H1N2	Negative
EPI_ISL_119343	A/swine/Minnesota/A01076998/2010	H1N2	Negative
EPI_ISL_129802	A/swine/Oklahoma/A01203865/2012	H1N2	Negative
EPI_ISL_218325	A/swine/Dong_Nai/08-22-2/2015/pAEpC1	H1N2	Negative
EPI_ISL_129488	A/swine/England/43142/2001	H1N2	Negative
EPI_ISL_151623	A/swine/North_Carolina/SG1232/2005	H1N2	Negative
EPI_ISL_150988	A/swine/Arkansas/SG1375/2011	H1N2	Negative
EPI_ISL_119348	A/swine/Minnesota/A01134236/2011	H1N2	Negative
EPI_ISL_136649	A/swine/Minnesota/A01444719/2013	H1N2	Negative
EPI_ISL_206452	A/swine/Wisconsin/A01104088/2015	H1N2	Negative
EPI_ISL_182281	A/swine/Oklahoma/A01566774/2014	H1N2	Negative
EPI_ISL_278839	A/swine/Italy/73436/2015	H1N2	Negative
EPI_ISL_216999	A/swine/North_Carolina/A01729907/2016	H1N2	Negative
EPI_ISL_150885	A/swine/Minnesota/01604/2007	H1N2	Negative
EPI_ISL_221738	A/swine/Illinois/A01733826/2016	H1N2	Negative
EPI_ISL_278880	A/swine/Italy/125010-1/2014	H1N2	Negative
EPI_ISL_131141	A/swine/Illinois/A01301796/2012	H1N2	Negative
EPI_ISL_142644	A/Swine/Spain/SF12091/2007	H1N2	Negative
EPI_ISL_79309	A/swine/Germany-NI/R299/09	H1N2	Negative
EPI_ISL_119332	A/swine/Iowa/A01076186/2010	H1N2	Negative
EPI_ISL_130052	A/swine/Illinois/A01240830/2011	H1N2	Negative
EPI_ISL_257156	A/swine/North_Carolina/A01672582/2017	H1N2	Negative
EPI_ISL_262600	A/swine/Iowa/A01667086/2017	H1N2	Negative
EPI_ISL_278838	A/swine/Italy/65210/2015	H1N2	Negative
EPI_ISL_245590	A/swine/North_Carolina/A01668056/2016	H1N2	Negative
EPI_ISL_207230	A/swine/Italy/50206-21/2014	H1N2	Negative
EPI_ISL_207956	A/swine/Manitoba/D0342/2014	H1N2	Negative
EPI_ISL_142645	A/Swine/Spain/80598LP4/2007	H1N2	Negative
EPI_ISL_161145	A/swine/Indiana/13TOSU1058/2013	H1N2	Negative
EPI_ISL_74377	A/swine/Hong_Kong/2314/2009	H1N2	Negative
EPI_ISL_153757	A/swine/North_Carolina/A01290777/2013	H1N2	Negative
EPI_ISL_97097	A/swine/Iowa/A01049645/2011	H1N2	Negative
EPI_ISL_218055	A/swine/Tien_Giang/08-10-1/2014	H1N2	Negative
EPI_ISL_195008	A/swine/France/Morbihan-0213/2011	H1N2	Negative

EPI_ISL_253709	A/swine/Pennsylvania/A01671487/2016	H1N2	Negative
EPI_ISL_124484	A/swine/Minnesota/A01134468/2011	H1N2	Negative
EPI_ISL_150997	A/swine/North_Carolina/SG1388/2011	H1N2	Negative
EPI_ISL_151180	A/swine/North_Carolina/SG1394/2011	H1N2	Negative
EPI_ISL_260805	A/swine/Indiana/A01932256/2017	H1N2	Negative
EPI_ISL_150932	A/swine/Kentucky/02564/2009	H1N2	Negative
EPI_ISL_129532	A/swine/Spain/40564/2002	H1N2	Negative
EPI_ISL_129450	A/swine/England/00003/2009	H1N2	Negative
EPI_ISL_129917	A/swine/North_Carolina/A01270393/2011	H1N2	Negative
EPI_ISL_218324	A/swine/Dong_Nai/08-22-2/2015	H1N2	Negative
EPI_ISL_243785	A/swine/North_Carolina/A01782003/2016	H1N2	Negative
EPI_ISL_195065	A/swine/Netherlands/Lievelde-511/2011	H1N2	Negative
EPI_ISL_207960	A/swine/Manitoba/D0348/2014	H1N2	Negative
EPI_ISL_243755	A/swine/Indiana/14TOSU6438/2014	H1N2	Negative
EPI_ISL_162584	A/swine/Kansas/A01377287/2014	H1N2	Negative
EPI_ISL_129491	A/swine/England/483/2006	H1N2	Negative
EPI_ISL_281248	A/swine/Manitoba/D0392/2015	H1N2	Negative
EPI_ISL_282514	A/swine/Alberta/SD0164/2016	H1N2	Negative
EPI_ISL_161654	A/swine/Bac_Ninh/347/2012	H1N2	Negative
EPI_ISL_151257	A/swine/Oklahoma/00259/2004	H1N2	Negative
EPI_ISL_127746	A/swine/Iowa/A01202083/2011	H1N2	Negative
EPI_ISL_235790	A/swine/North_Carolina/A01778770/2016	H1N2	Negative
EPI_ISL_256251	A/swine/Indiana/A01812062/2015	H1N2	Negative
EPI_ISL_281303	A/swine/North_Carolina/A02218894/2017	H1N2	Negative
EPI_ISL_198366	A/swine/South_Dakota/A02025413/2015	H1N2	Negative
EPI_ISL_151249	A/swine/Colorado/02875/2009	H1N2	Negative
EPI_ISL_153730	A/swine/Kansas/A01290646/2013	H1N2	Negative
EPI_ISL_278912	A/swine/Italy/337085/2012	H1N2	Negative
EPI_ISL_205518	A/swine/Pennsylvania/A02035484/2015	H1N2	Negative
EPI_ISL_195003	A/swine/Germany/Nikolausdorf-IDT12279/2010	H1N2	Negative
EPI_ISL_225506	A/swine/North_Carolina/A01894897/2016	H1N2	Negative
EPI_ISL_235793	A/swine/Iowa/A01778876/2016	H1N2	Negative
EPI_ISL_234673	A/swine/Iowa/A01777892/2016	H1N2	Negative
EPI_ISL_207229	A/swine/Italy/50064-3/2014	H1N2	Negative
EPI_ISL_153862	A/swine/Nebraska/A01104071/2013	H1N2	Negative
EPI_ISL_296421	A/swine/Iowa/A02221865/2017	H1N2	Negative
EPI_ISL_127813	A/swine/Iowa/A01202809/2011	H1N2	Negative
EPI_ISL_218585	A/swine/Dong_Nai/07-11-3/2015	H1N2	Negative
EPI_ISL_195117	A/swine/England/1437/2004	H1N2	Negative
EPI_ISL_203174	A/swine/North_Carolina/A01944409/2015	H1N2	Negative
EPI_ISL_218588	A/swine/Dong_Nai/07-14-3/2015	H1N2	Negative
EPI_ISL_254685	A/swine/Nebraska/A01672049/2017	H1N2	Negative
EPI_ISL_252699	A/swine/Colorado/A01668653/2016	H1N2	Negative
EPI_ISL_195012	A/swine/France/IlleetVilaine-0415/2011	H1N2	Negative
EPI_ISL_282912	A/swine/Illinois/A02219794/2017	H1N2	Negative
EPI_ISL_24578	A/swine/Korea/PZ4/2006	H1N2	Negative
EPI_ISL_140964	A/swine/Nebraska/A01443969/2013	H1N2	Negative
EPI_ISL_212187	A/swine/Nebraska/A01941636/2015	H1N2	Negative
EPI_ISL_173750	A/swine/Oklahoma/A01567187/2014	H1N2	Negative
EPI_ISL_163190	A/swine/TienGiang/01-30-1/2012	H1N2	Negative
EPI_ISL_218326	A/swine/Dong_Nai/08-29-2/2015	H1N2	Negative
EPI_ISL_207046	A/swine/Manitoba/D0364/2012	H1N2	Negative
EPI_ISL_150995	A/swine/Virginia/SG1386/2011	H1N2	Negative
EPI_ISL_127770	A/swine/Oklahoma/A01202498/2011	H1N2	Negative
EPI_ISL_24574	A/swine/Korea/JL01/2005	H1N2	Negative
EPI_ISL_203109	A/swine/North_Carolina/A01945748/2015	H1N2	Negative
EPI_ISL_163045	A/swine/DongNai/07-19-2/2013	H1N2	Negative
EPI_ISL_177503	A/swine/Minnesota/01082/2006	H1N2	Negative

EPI_ISL_222479	A/swine/Nebraska/A01894200/2016	H1N2	Negative
EPI_ISL_278832	A/swine/Italy/187946/2015	H1N2	Negative
EPI_ISL_119341	A/swine/Minnesota/A01076763/2010	H1N2	Negative
EPI_ISL_135961	A/swine/Michigan/A01259001/2012	H1N2	Negative
EPI_ISL_218321	A/swine/Dong_Nai/08-13-2/2015	H1N2	Negative
EPI_ISL_129484	A/swine/England/40280/1996	H1N2	Negative
EPI_ISL_129497	A/swine/England/645913/1996	H1N2	Negative
EPI_ISL_145690	A/swine/North_Carolina/A01432952/2013	H1N2	Negative
EPI_ISL_278865	A/swine/Italy/302073/2014	H1N2	Negative
EPI_ISL_95876	A/swine/Italy/233139/2005	H1N2	Negative
EPI_ISL_195081	A/swine/England/997/2008	H1N2	Negative
EPI_ISL_12996	A/swine/Cloppenburg/IDT4777/2005	H1N2	Negative
EPI_ISL_245071	A/swine/Kansas/A01782583/2016	H1N2	Negative
EPI_ISL_97138	A/swine/Iowa/A01049765/2011	H1N2	Negative
EPI_ISL_163048	A/swine/DongNai/07-10-2/2013	H1N2	Negative
EPI_ISL_88371	A/swine/Hong_Kong/69/2006	H1N2	Negative
EPI_ISL_9879	A/swine/Ontario/55383/04	H1N2	Negative
EPI_ISL_195002	A/swine/Germany/Barle-IDT13149/2011	H1N2	Negative
EPI_ISL_278873	A/swine/Italy/166277-2/2014	H1N2	Negative
EPI_ISL_195183	A/swine/Netherlands/Wanroij-CVI2117A/2012	H1N2	Negative
EPI_ISL_328812	A/swine/Chile/VN1401-274/2014	H1N2	Negative
EPI_ISL_258614	A/swine/Illinois/A01672615/2017	H1N2	Negative
EPI_ISL_218303	A/swine/Dong_Nai/09-30-1/2015/pAEpC1	H1N2	Negative
EPI_ISL_132825	A/swine/Korea/VDS2/2009	H1N2	Negative
EPI_ISL_195113	A/swine/Spain/29262/2012	H1N2	Negative
EPI_ISL_25229	A/swine/Shanghai/1/2007	H1N2	Negative
EPI_ISL_253720	A/swine/Iowa/A01671454/2016	H1N2	Negative
EPI_ISL_151990	A/swine/Iowa/13C096/2013	H1N2	Negative
EPI_ISL_195111	A/swine/Denmark/10-1391-3/2011	H1N2	Negative
EPI_ISL_278872	A/swine/Italy/165246/2014	H1N2	Negative
EPI_ISL_150873	A/swine/Oklahoma/01741/2007	H1N2	Negative
EPI_ISL_256249	A/swine/Indiana/A01812028/2015	H1N2	Negative
EPI_ISL_235726	A/swine/California/A01778665/2016	H1N2	Negative
EPI_ISL_195079	A/swine/France/CotesdArmor-0558/2011	H1N2	Negative
EPI_ISL_271063	A/swine/South_Dakota/MT_12_07_2081/2012	H1N2	Negative
EPI_ISL_150846	A/swine/Texas/01308/2006	H1N2	Negative
EPI_ISL_297208	A/swine/Iowa/A02137183/2017	H1N2	Negative
EPI_ISL_207039	A/swine/Manitoba/D0171/2012	H1N2	Negative
EPI_ISL_129558	A/swine/Gent/100/2007	H1N2	Negative
EPI_ISL_207921	A/swine/Manitoba/D0341/2014	H1N2	Negative
EPI_ISL_96921	A/swine/Indiana/00427/2004	H1N2	Negative
EPI_ISL_129498	A/swine/England/661263/1997	H1N2	Negative
EPI_ISL_129505	A/swine/England/88761/1997	H1N2	Negative
EPI_ISL_218299	A/swine/Dong_Nai/09-16-1/2015	H1N2	Negative
EPI_ISL_195017	A/swine/Italy/57680/2011	H1N2	Negative
EPI_ISL_150921	A/swine/North_Carolina/02249/2008	H1N2	Negative
EPI_ISL_262586	A/swine/North_Carolina/A01667104/2017	H1N2	Negative
EPI_ISL_281310	A/swine/North_Carolina/A02218906/2017	H1N2	Negative
EPI_ISL_151643	A/swine/Iowa/SG1290/2007	H1N2	Negative
EPI_ISL_200143	A/swine/Indiana/A01260943/2015	H1N2	Negative
EPI_ISL_195157	A/swine/France/22-120228/2012	H1N2	Negative
EPI_ISL_142690	A/swine/Korea/CY12-03/2011	H1N2	Negative
EPI_ISL_129495	A/swine/England/61605/1998	H1N2	Negative
EPI_ISL_95877	A/swine/Italy/198260/2008	H1N2	Negative
EPI_ISL_24576	A/swine/Korea/JL04/2005	H1N2	Negative
EPI_ISL_278853	A/swine/Italy/333140/2014	H1N2	Negative
EPI_ISL_195062	A/swine/France/CotesdArmor-0198/2011	H1N2	Negative
EPI_ISL_234291	A/swine/Indiana/A01812224/2016	H1N2	Negative

EPI_ISL_127790	A/swine/Iowa/A01202677/2011	H1N2	Negative
EPI_ISL_153853	A/swine/Iowa/13H072/2013	H1N2	Negative
EPI_ISL_195018	A/swine/Italy/275736/2010	H1N2	Negative
EPI_ISL_218323	A/swine/Dong_Nai/08-19-2/2015	H1N2	Negative
EPI_ISL_124037	A/swine/Zhejiang/01/2008	H1N2	Negative
EPI_ISL_150986	A/swine/Minnesota/SG1370/2010	H1N2	Negative
EPI_ISL_195158	A/swine/France/37-120345/2012	H1N2	Negative
EPI_ISL_218586	A/swine/Dong_Nai/07-12-3/2015	H1N2	Negative
EPI_ISL_151197	A/swine/North_Carolina/SG1335/2009	H1N2	Negative
EPI_ISL_132379	A/swine/Nebraska/A01270646/2012	H1N2	Negative
EPI_ISL_278852	A/swine/Italy/328356/2014	H1N2	Negative
EPI_ISL_243780	A/swine/Indiana/15TOSU0829/2015	H1N2	Negative
EPI_ISL_261459	A/swine/Iowa/A01932419/2017	H1N2	Negative
EPI_ISL_147152	A/swine/Virginia/A01432745/2013	H1N2	Negative
EPI_ISL_153861	A/swine/Nebraska/A01104068/2013	H1N2	Negative
EPI_ISL_200935	A/swine/Indiana/A01260933/2015	H1N2	Negative
EPI_ISL_195000	A/swine/Germany/Lohne-IDT12877/2011	H1N2	Negative
EPI_ISL_218581	A/swine/Dong_Nai/07-04-3/2015	H1N2	Negative
EPI_ISL_88369	A/swine/Hong_Kong/729/2005	H1N2	Negative
EPI_ISL_12995	A/swine/Doetlingen/IDT4735/2005	H1N2	Negative
EPI_ISL_129445	A/swine/Korea/1204/2009	H1N2	Negative
EPI_ISL_211807	A/swine/Ohio/A01728547/2015	H1N2	Negative
EPI_ISL_145746	A/swine/Missouri/A01432837/2013	H1N2	Negative
EPI_ISL_195083	A/swine/Spain/79976/2007	H1N2	Negative
EPI_ISL_88843	A/swine/Minnesota/239106/2010	H1N2	Negative
EPI_ISL_195026	A/swine/Netherlands/Haaksbergen-136/2011	H1N2	Negative
EPI_ISL_272837	A/swine/South_Carolina/A02214849/2017	H1N2	Negative
EPI_ISL_24581	A/swine/Korea/CY08/2007	H1N2	Negative
EPI_ISL_234526	A/swine/Iowa/A01778108/2016	H1N2	Negative
EPI_ISL_95808	A/swine/Minnesota/1192/2001	H1N2	Negative
EPI_ISL_143193	A/swine/Ohio/11SW162/2011	H1N2	Negative
EPI_ISL_203194	A/swine/Pennsylvania/A01944215/2015	H1N2	Negative
EPI_ISL_164464	A/swine/DongNai/03-29-2/2011	H1N2	Negative
EPI_ISL_154633	A/swine/Oklahoma/A01290898/2013	H1N2	Negative
EPI_ISL_129444	A/swine/Korea/1130/2009	H1N2	Negative
EPI_ISL_150840	A/swine/North_Carolina/01169/2006	H1N2	Negative
EPI_ISL_88351	A/swine/Hong_Kong/1304/2003	H1N2	Negative
EPI_ISL_296426	A/swine/Iowa/A02134236/2017	H1N2	Negative
EPI_ISL_208974	A/swine/Iowa/A01796529/2015	H1N2	Negative
EPI_ISL_119338	A/swine/Minnesota/A01076210/2010	H1N2	Negative
EPI_ISL_164465	A/swine/DongNai/03-30-2/2011	H1N2	Negative
EPI_ISL_235746	A/swine/North_Carolina/A01732695/2016	H1N2	Negative
EPI_ISL_226061	A/swine/Minnesota/A01943452/2016	H1N2	Negative
EPI_ISL_237046	A/swine/North_Carolina/A01781221/2016	H1N2	Negative
EPI_ISL_150099	A/swine/Iowa/13B092/2013	H1N2	Negative
EPI_ISL_218322	A/swine/Dong_Nai/08-18-2/2015	H1N2	Negative
EPI_ISL_271735	A/swine/Iowa/MT_12_07_11_2_3/2011	H1N2	Negative
EPI_ISL_95867	A/swine/Italy/62/1998	H1N2	Negative
EPI_ISL_119339	A/swine/Minnesota/A01076331/2010	H1N2	Negative
EPI_ISL_162678	A/swine/Bac_Ninh/354/2013	H1N2	Negative
EPI_ISL_150998	A/swine/North_Carolina/SG1389/2011	H1N2	Negative
EPI_ISL_140906	A/swine/Kentucky/12TOSU1053/2012	H1N2	Negative
EPI_ISL_218598	A/swine/Dong_Nai/07-29-3/2015	H1N2	Negative
EPI_ISL_205921	A/swine/France/22-130212/2013	H1N2	Negative
EPI_ISL_103850	A/swine/North_Carolina/A01048807/2011	H1N2	Negative
EPI_ISL_195179	A/swine/Netherlands/Lemele-CVI2974A/2013	H1N2	Negative
EPI_ISL_245789	A/swine/Indiana/15TOSU2743/2015	H1N2	Negative
EPI_ISL_95866	A/swine/Italy/3592/1999	H1N2	Negative

EPI_ISL_127771	A/swine/Nebraska/A01202501/2011	H1N2	Negative
EPI_ISL_95872	A/swine/Italy/81226/2009	H1N2	Negative
EPI_ISL_218281	A/swine/Dong_Nai/07-17-1/2015	H1N2	Negative
EPI_ISL_207160	A/swine/Manitoba/D0297/2013	H1N2	Negative
EPI_ISL_195120	A/swine/England/000304/2009	H1N2	Negative
EPI_ISL_195080	A/swine/France/22-110529/2011	H1N2	Negative
EPI_ISL_96980	A/swine/Hong_Kong/2237/2010	H1N2	Negative
EPI_ISL_258653	A/swine/Minnesota/A01932042/2017	H1N2	Negative
EPI_ISL_278893	A/swine/Italy/60011-3/2014	H1N2	Negative
EPI_ISL_254677	A/swine/North_Carolina/A01672048/2017	H1N2	Negative
EPI_ISL_139268	A/swine/Nebraska/A01445765/2013	H1N2	Negative
EPI_ISL_88353	A/swine/Hong_Kong/1578/2003	H1N2	Negative
EPI_ISL_218583	A/swine/Dong_Nai/07-07-3/2015	H1N2	Negative
EPI_ISL_163875	A/swine/Nebraska/A01104081/2014	H1N2	Negative
EPI_ISL_198161	A/swine/Indiana/A01260906/2015	H1N2	Negative
EPI_ISL_74372	A/swine/Hong_Kong/1479/2009	H1N2	Negative
EPI_ISL_139266	A/swine/Minnesota/A01445762/2013	H1N2	Negative
EPI_ISL_245321	A/swine/Iowa/A01782577/2016	H1N2	Negative
EPI_ISL_285408	A/swine/Iowa/A02221491/2017	H1N2	Negative
EPI_ISL_195037	A/swine/Belgium/Oostkamp-26/2012	H1N2	Negative
EPI_ISL_91640	A/swine/Hubei/05/2009	H1N2	Negative
EPI_ISL_195446	A/swine/South_Dakota/A01823237/2015	H1N2	Negative
EPI_ISL_278905	A/swine/Italy/198930-3/2013	H1N2	Negative
EPI_ISL_161135	A/swine/Indiana/13TOSU1160/2013	H1N2	Negative
EPI_ISL_97088	A/swine/Oklahoma/A01049501/2011	H1N2	Negative
EPI_ISL_95805	A/swine/Minnesota/5763/2003	H1N2	Negative
EPI_ISL_230188	A/swine/Iowa/A01775725/2016	H1N2	Negative
EPI_ISL_168302	A/swine/Texas/13TOSU0049/2013	H1N2	Negative
EPI_ISL_246096	A/swine/Minnesota/A01668523/2016	H1N1	Negative
EPI_ISL_195144	A/swine/Belgium/Glabbeek-287/2012	H1N1	Negative
EPI_ISL_151611	A/swine/North_Carolina/SG1172/2003	H1N1	Negative
EPI_ISL_68984	A/swine/Italy/671/1987	H1N1	Negative
EPI_ISL_196000	A/swine/Mexico/8935602/2012	H1N1	Negative
EPI_ISL_280483	A/swine/North_Carolina/A01785282/2017	H1N1	Negative
EPI_ISL_151609	A/swine/Kentucky/SG1167/2003	H1N1	Negative
EPI_ISL_207053	A/swine/Manitoba/D0175/2012	H1N1	Negative
EPI_ISL_88287	A/swine/Hong_Kong/90/1979	H1N1	Negative
EPI_ISL_129483	A/swine/England/39572/2001	H1N1	Negative
EPI_ISL_280477	A/swine/Ohio/17TOSU1386/2017	H1N1	Negative
EPI_ISL_157893	A/swine/Guangxi/G2/2013	H1N1	Negative
EPI_ISL_145707	A/swine/Illinois/A00857317F/2012	H1N1	Negative
EPI_ISL_151260	A/swine/Oklahoma/SG1457/2004	H1N1	Negative
EPI_ISL_222129	A/swine/Germany/R1738/2010	H1N1	Negative
EPI_ISL_130023	A/swine/Iowa/A01328941/2012	H1N1	Negative
EPI_ISL_200895	A/swine/Indiana/14TOSU0454/2014	H1N1	Negative
EPI_ISL_167433	A/swine/Ohio/A01476448/2014	H1N1	Negative
EPI_ISL_90106	A/swine/Nebraska/A01049048/2010	H1N1	Negative
EPI_ISL_216994	A/swine/North_Carolina/A01730376/2016	H1N1	Negative
EPI_ISL_151607	A/swine/Texas/00195/2003	H1N1	Negative
EPI_ISL_129482	A/swine/England/383/2005	H1N1	Negative
EPI_ISL_97093	A/swine/Illinois/A01049574/2011	H1N1	Negative
EPI_ISL_205083	A/swine/North_Carolina/A01795841/2015	H1N1	Negative
EPI_ISL_138994	A/swine/Iowa/A0143271/2013	H1N1	Negative
EPI_ISL_151606	A/swine/Texas/SG1162/2003	H1N1	Negative
EPI_ISL_156556	A/swine/Illinois/A01427360/2013	H1N1	Negative
EPI_ISL_308752	A/Swine/Belzig/02/2001	H1N1	Negative
EPI_ISL_88234	A/swine/Hong_Kong/51/1977	H1N1	Negative
EPI_ISL_199473	A/swine/Gent/74/95	H1N1	Negative

EPI_ISL_140962	A/swine/Nebraska/A01443544/2013	H1N1	Negative
EPI_ISL_285335	A/swine/Iowa/A02221508/2017	H1N1	Negative
EPI_ISL_151207	A/swine/Manitoba/01781/2007	H1N1	Negative
EPI_ISL_180682	A/swine/Iowa/A01489009/2015	H1N1	Negative
EPI_ISL_96917	A/swine/Illinois/02064/2008	H1N1	Negative
EPI_ISL_296675	A/swine/North_Carolina/A02223226/2017	H1N1	Negative
EPI_ISL_150834	A/swine/Minnesota/00938/2005	H1N1	Negative
EPI_ISL_206673	A/swine/Indiana/A01260972/2015	H1N1	Negative
EPI_ISL_195068	A/swine/France/IlleetVilaine-0266/2011	H1N1	Negative
EPI_ISL_282074	A/swine/Guangxi/BB2/2013	H1N1	Negative
EPI_ISL_124007	A/swine/Iowa/A01076187/2010	H1N1	Negative
EPI_ISL_153740	A/swine/Oklahoma/A01290693/2013	H1N1	Negative
EPI_ISL_195056	A/swine/Spain/22013/2011	H1N1	Negative
EPI_ISL_129515	A/swine/Ille_et_Vilaine/1455/1999	H1N1	Negative
EPI_ISL_151266	A/swine/Minnesota/SG1470/2004	H1N1	Negative
EPI_ISL_150835	A/swine/Arkansas/SG1241/2006	H1N1	Negative
EPI_ISL_235734	A/swine/Iowa/A01778712/2016	H1N1	Negative
EPI_ISL_129454	A/swine/England/010402/2003	H1N1	Negative
EPI_ISL_95804	A/swine/Italy/1369-7/1994	H1N1	Negative
EPI_ISL_282540	A/swine/Manitoba/D0438/2016	H1N1	Negative
EPI_ISL_201851	A/swine/Missouri/A01945149/2015	H1N1	Negative
EPI_ISL_237048	A/swine/Iowa/A01781046/2016	H1N1	Negative
EPI_ISL_278926	A/swine/Italy/122595/2012	H1N1	Negative
EPI_ISL_195035	A/swine/France/LoireAtlantique-0405/2011	H1N1	Negative
EPI_ISL_262595	A/swine/Iowa/A01667091/2017	H1N1	Negative
EPI_ISL_28425	A/swine/Ratchaburi/NIAH1481/2000	H1N1	Negative
EPI_ISL_127780	A/swine/Mississippi/A01202593/2011	H1N1	Negative
EPI_ISL_278874	A/swine/Italy/179794/2014	H1N1	Negative
EPI_ISL_278846	A/swine/Italy/10915/2015	H1N1	Negative
EPI_ISL_208983	A/swine/Missouri/A01796703/2015	H1N1	Negative
EPI_ISL_28428	A/swine/Ratchaburi/NIAH550/2003	H1N1	Negative
EPI_ISL_95807	A/swine/North_Carolina/47834/2000	H1N1	Negative
EPI_ISL_256255	A/swine/Indiana/A01812068/2015	H1N1	Negative
EPI_ISL_129455	A/swine/England/101692/1997	H1N1	Negative
EPI_ISL_195052	A/swine/Denmark/1085-1/2012	H1N1	Negative
EPI_ISL_218331	A/swine/Shandong/S269/2014	H1N1	Negative
EPI_ISL_195023	A/swine/Belgium/Gent-560/2010	H1N1	Negative
EPI_ISL_129492	A/swine/England/57610/1999	H1N1	Negative
EPI_ISL_254875	A/swine/Minnesota/A01672344/2017	H1N1	Negative
EPI_ISL_151610	A/swine/North_Carolina/SG1170/2003	H1N1	Negative
EPI_ISL_88401	A/swine/Hong_Kong/414/2009	H1N1	Negative
EPI_ISL_29412	A/swine/France/WVL13/1995	H1N1	Negative
EPI_ISL_216913	A/swine/Minnesota/A01942199/2016	H1N1	Negative
EPI_ISL_8009	A/swine/Iowa/2/1987	H1N1	Negative
EPI_ISL_311324	A/swine/Ohio/OH-18-7963/2018	H1N1	Negative
EPI_ISL_281247	A/swine/Alberta/SD0154/2016	H1N1	Negative
EPI_ISL_211811	A/swine/Illinois/A01728329/2015	H1N1	Negative
EPI_ISL_142642	A/Swine/Spain/SF11131/2007	H1N1	Negative
EPI_ISL_195160	A/swine/France/01-130054/2013	H1N1	Negative
EPI_ISL_88361	A/swine/Hong_Kong/NS856/2004	H1N1	Negative
EPI_ISL_278860	A/swine/Italy/244784/2014	H1N1	Negative
EPI_ISL_88359	A/swine/Hong_Kong/NS318/2004	H1N1	Negative
EPI_ISL_129526	A/swine/Italy/186678-2/2005	H1N1	Negative
EPI_ISL_282495	A/swine/Quebec/SD0175/2016	H1N1	Negative
EPI_ISL_181106	A/swine/North_Carolina/A01841602/2015	H1N1	Negative
EPI_ISL_173623	A/swine/Missouri/A01567015/2014	H1N1	Negative
EPI_ISL_281242	A/swine/Saskatchewan/SD0094/2015	H1N1	Negative
EPI_ISL_195162	A/swine/Poland/1744/2012	H1N1	Negative

EPI_ISL_151200	A/swine/Oklahoma/SG1349/2010	H1N1	Negative
EPI_ISL_151293	A/swine/North_Carolina/01514/2007	H1N1	Negative
EPI_ISL_91942	A/swine/Minnesota/00272/2004	H1N1	Negative
EPI_ISL_181107	A/swine/North_Carolina/A01841556/2015	H1N1	Negative
EPI_ISL_199471	A/swine/Gent/196/92	H1N1	Negative
EPI_ISL_116999	A/swine/Iowa/A01076215/2010	H1N1	Negative
EPI_ISL_93362	A/swine/Iowa/A01049379/2011	H1N1	Negative
EPI_ISL_143442	A/swine/Nebraska/A01380503/2013	H1N1	Negative
EPI_ISL_207114	A/swine/Manitoba/D0270/2013	H1N1	Negative
EPI_ISL_74384	A/swine/Hong_Kong/NS1065/2009	H1N1	Negative
EPI_ISL_129467	A/swine/England/193667/1998	H1N1	Negative
EPI_ISL_263482	A/swine/Indiana/16TOSU6511/2016	H1N1	Negative
EPI_ISL_129471	A/swine/England/252411/1992	H1N1	Negative
EPI_ISL_29116	A/swine/Greven/IDT2889/2004	H1N1	Negative
EPI_ISL_195124	A/swine/England/1251/2011	H1N1	Negative
EPI_ISL_252710	A/swine/Iowa/A01776531/2016	H1N1	Negative
EPI_ISL_207169	A/swine/Alberta/SD0014/2013	H1N1	Negative
EPI_ISL_278897	A/swine/Italy/311368/2013	H1N1	Negative
EPI_ISL_282075	A/swine/Guangxi/GG6/2013	H1N1	Negative
EPI_ISL_96915	A/swine/Minnesota/001200/2006	H1N1	Negative
EPI_ISL_195151	A/swine/Belgium/111/2013	H1N1	Negative
EPI_ISL_194996	A/swine/Germany/Lohne-IDT12137/2010	H1N1	Negative
EPI_ISL_150836	A/swine/Oklahoma/SG1243/2006	H1N1	Negative
EPI_ISL_88379	A/swine/Hong_Kong/72/2007	H1N1	Negative
EPI_ISL_129466	A/swine/England/17788/2000	H1N1	Negative
EPI_ISL_129472	A/swine/England/254/2002	H1N1	Negative
EPI_ISL_258623	A/swine/Missouri/A01672819/2017	H1N1	Negative
EPI_ISL_150930	A/swine/Oklahoma/SG1325/2009	H1N1	Negative
EPI_ISL_161195	A/swine/Texas/13TOSU0050/2013	H1N1	Negative
EPI_ISL_97070	A/swine/Iowa/A01049797/2011	H1N1	Negative
EPI_ISL_208942	A/swine/Illinois/A01796450/2015	H1N1	Negative
EPI_ISL_278898	A/swine/Italy/327666/2013	H1N1	Negative
EPI_ISL_95806	A/swine/Minnesota/6998/2003	H1N1	Negative
EPI_ISL_150837	A/swine/Oklahoma/SG1244/2006	H1N1	Negative
EPI_ISL_29424	A/swine/Denmark/WVL9/1993	H1N1	Negative
EPI_ISL_129453	A/swine/England/004772/2001	H1N1	Negative
EPI_ISL_195172	A/swine/Poland/006801/2013	H1N1	Negative
EPI_ISL_195033	A/swine/Netherlands/Putten-60/2012	H1N1	Negative
EPI_ISL_129469	A/swine/England/200276/1992	H1N1	Negative
EPI_ISL_151616	A/swine/Oklahoma/SG1188/2004	H1N1	Negative
EPI_ISL_261464	A/swine/Iowa/A01932420/2017	H1N1	Negative
EPI_ISL_29408	A/swine/Belgium/WVL1/1979	H1N1	Negative
EPI_ISL_246030	A/swine/Arkansas/A01668226/2016	H1N1	Negative
EPI_ISL_195163	A/swine/Poland/321/2012	H1N1	Negative
EPI_ISL_195130	A/swine/Scotland/034632/2012	H1N1	Negative
EPI_ISL_256261	A/swine/Indiana/A01812063/2015	H1N1	Negative
EPI_ISL_195173	A/swine/Spain/33903/2012	H1N1	Negative
EPI_ISL_29414	A/swine/England/WVL15/1997	H1N1	Negative
EPI_ISL_92314	A/swine/Missouri/00564/2005	H1N1	Negative
EPI_ISL_151267	A/swine/Oklahoma/00801/2005	H1N1	Negative
EPI_ISL_196019	A/swine/Mexico/9783445/2013	H1N1	Negative
EPI_ISL_151274	A/swine/North_Carolina/01550/2007	H1N1	Negative
EPI_ISL_195007	A/swine/France/IlleetVilaine-0187/2011	H1N1	Negative
EPI_ISL_5288	A/swine/Spain/53207/2004	H1N1	Negative
EPI_ISL_29409	A/swine/England/WVL10/1993	H1N1	Negative
EPI_ISL_243375	A/swine/North_Carolina/A01778828/2016	H1N1	Negative
EPI_ISL_195028	A/swine/Belgium/Poperinge-387/2011	H1N1	Negative
EPI_ISL_246088	A/swine/Iowa/A01668528/2016	H1N1	Negative

EPI_ISL_96929	A/swine/Illinois/SG1141/2003	H1N1	Negative
EPI_ISL_96926	A/swine/Minnesota/01358/2006	H1N1	Negative
EPI_ISL_151264	A/swine/Iowa/00250/2004	H1N1	Negative
EPI_ISL_285478	A/swine/Minnesota/A02221496/2017	H1N1	Negative
EPI_ISL_151202	A/swine/Texas/SG1380/2011	H1N1	Negative
EPI_ISL_129465	A/swine/England/17787/2000	H1N1	Negative
EPI_ISL_151989	A/swine/Iowa/13E088/2013	H1N1	Negative
EPI_ISL_199469	A/swine/Belgium/1/79	H1N1	Negative
EPI_ISL_197216	A/swine/Iowa/A02025218/2015	H1N1	Negative
EPI_ISL_195041	A/swine/Belgium/Neerpelt-37/2011	H1N1	Negative
EPI_ISL_151215	A/swine/North_Carolina/SG1281/2007	H1N1	Negative
EPI_ISL_96925	A/swine//Ohio/02026/2008	H1N1	Negative
EPI_ISL_88273	A/swine/Hong_Kong/66/1977	H1N1	Negative
EPI_ISL_207980	A/swine/Saskatchewan/SD0056/2014	H1N1	Negative
EPI_ISL_281967	A/swine/Minnesota/MT_13_01_S49/2013	H1N1	Negative
EPI_ISL_199476	A/swine/England/195852/92	H1N1	Negative
EPI_ISL_195153	A/swine/France/22-120146/2012	H1N1	Negative
EPI_ISL_195086	A/swine/Germany/Ahlen-Dolberg-IDT14631/2012	H1N1	Negative
EPI_ISL_88399	A/swine/Hong_Kong/NS184/2009	H1N1	Negative
EPI_ISL_222094	A/swine/Iowa/A01732729/2016	H1N1	Negative
EPI_ISL_129464	A/swine/England/167655/1997	H1N1	Negative
EPI_ISL_88264	A/swine/Hong_Kong/55/1977	H1N1	Negative
EPI_ISL_150830	A/swine/Oklahoma/SG1217/2005	H1N1	Negative
EPI_ISL_278862	A/swine/Italy/263752/2014	H1N1	Negative
EPI_ISL_260813	A/swine/North_Carolina/A01672751/2017	H1N1	Negative
EPI_ISL_150870	A/swine/Texas/SG1271/2007	H1N1	Negative
EPI_ISL_195266	A/swine/Poland/18275/2012	H1N1	Negative
EPI_ISL_167340	A/swine/Oklahoma/A01476227/2014	H1N1	Negative
EPI_ISL_124335	A/swine/Illinois/A01076767/2010	H1N1	Negative
EPI_ISL_134283	A/swine/Illinois/A01244401/2012	H1N1	Negative
EPI_ISL_195011	A/swine/France/Finistere-0011/2011	H1N1	Negative
EPI_ISL_127701	A/swine/Minnesota/A01203313/2012	H1N1	Negative
EPI_ISL_195032	A/swine/Netherlands/Meijel-75/2011	H1N1	Negative
EPI_ISL_151291	A/swine/North_Carolina/01591/2007	H1N1	Negative
EPI_ISL_129573	A/swine/Gent/132/2005	H1N1	Negative
EPI_ISL_195154	A/swine/France/50-120350/2012	H1N1	Negative
EPI_ISL_285329	A/swine/Iowa/A02221659/2017	H1N1	Negative
EPI_ISL_151621	A/swine/Arkansas/SG1214/2005	H1N1	Negative
EPI_ISL_12792	A/swine/Tianjin/01/04	H1N1	Negative
EPI_ISL_138215	A/swine/Minnesota/A01445265/2013	H1N1	Negative
EPI_ISL_150996	A/swine/North_Carolina/SG1387/2011	H1N1	Negative
EPI_ISL_304092	A/swine/Czech_Republic/25-18/2017	H1N1	Negative
EPI_ISL_281751	A/swine/Ohio/A01354304/2017	H1N1	Negative
EPI_ISL_88239	A/swine/Hong_Kong/28/1977	H1N1	Negative
EPI_ISL_150887	A/swine/Texas/01522/2007	H1N1	Negative
EPI_ISL_129451	A/swine/England/001195/2007	H1N1	Negative
EPI_ISL_195054	A/swine/Denmark/10146-1/2012	H1N1	Negative
EPI_ISL_140918	A/swine/Ohio/12TOSU45/2012	H1N1	Negative
EPI_ISL_151614	A/swine/Missouri/SG1182/2003	H1N1	Negative
EPI_ISL_278834	A/swine/Italy/203977/2015	H1N1	Negative
EPI_ISL_29416	A/swine/Scotland/WVL17/1999	H1N1	Negative
EPI_ISL_169560	A/swine/Guangdong/6/2013	H1N1	Negative
EPI_ISL_129496	A/swine/England/636804/1996	H1N1	Negative
EPI_ISL_145714	A/swine/Italy/73449/2013	H1N1	Negative
EPI_ISL_205071	A/swine/North_Carolina/A01795758/2015	H1N1	Negative
EPI_ISL_282706	A/swine/Saskatchewan/SD0108/2015	H1N1	Negative
EPI_ISL_151916	A/swine/Minnesota/A01394863/2013	H1N1	Negative
EPI_ISL_129510	A/wild_boar/Germany-HE/ws336/2009	H1N1	Negative

EPI_ISL_252802	A/swine/North_Carolina/A01668901/2016	H1N1	Negative
EPI_ISL_129525	A/swine/Italy/172336/2001	H1N1	Negative
EPI_ISL_150845	A/swine/Minnesota/00991/2006	H1N1	Negative
EPI_ISL_129807	A/swine/Iowa/A01203809/2012	H1N1	Negative
EPI_ISL_281985	A/swine/Ohio/A02219547/2017	H1N1	Negative
EPI_ISL_253925	A/swine/Oklahoma/A01671948/2016	H1N1	Negative
EPI_ISL_278801	A/swine/Quebec/1587352/2014	H1N1	Negative
EPI_ISL_195031	A/swine/Belgium/Faimes-628/2010	H1N1	Negative
EPI_ISL_132827	A/swine/Korea/VDS3/2009	H1N1	Negative
EPI_ISL_257154	A/swine/Oklahoma/A01672680/2017	H1N1	Negative
EPI_ISL_151265	A/swine/Minnesota/00352/2004	H1N1	Negative
EPI_ISL_271667	A/swine/Minnesota/MT_12_07_11_3_12/2011	H1N1	Negative
EPI_ISL_96924	A/swine/North_Carolina/00839/2005	H1N1	Negative
EPI_ISL_127901	A/swine/Illinois/A00857136c/2011	H1N1	Negative
EPI_ISL_195005	A/swine/Germany/Ostbevern-IDT13211/2011	H1N1	Negative
EPI_ISL_208916	A/swine/North_Carolina/A01797415/2015	H1N1	Negative
EPI_ISL_88365	A/swine/Hong_Kong/275/2005	H1N1	Negative
EPI_ISL_173677	A/swine/Arkansas/A01840184/2014	H1N1	Negative
EPI_ISL_129970	A/swine/Minnesota/A01301732/2012	H1N1	Negative
EPI_ISL_79273	A/swine/Germany-NI/R248/09	H1N1	Negative
EPI_ISL_150922	A/swine/North_Carolina/SG1310/2008	H1N1	Negative
EPI_ISL_90125	A/swine/Minnesota/A01049101/2010	H1N1	Negative
EPI_ISL_138858	A/swine/Iowa/A01445629/2013	H1N1	Negative
EPI_ISL_128170	A/swine/Mexico/Ver37/2010	H1N1	Negative
EPI_ISL_153768	A/swine/North_Carolina/A01290771/2013	H1N1	Negative
EPI_ISL_93366	A/swine/Minnesota/A01049462/2011	H1N1	Negative
EPI_ISL_195380	A/swine/Pennsylvania/A02024933/2015	H1N1	Negative
EPI_ISL_161167	A/swine/Indiana/13TOSU1988/2013	H1N1	Negative
EPI_ISL_74378	A/swine/Hong_Kong/2433/2009	H1N1	Negative
EPI_ISL_117003	A/swine/Minnesota/A01134527/2011	H1N1	Negative
EPI_ISL_199475	A/swine/Belgium/1/83	H1N1	Negative
EPI_ISL_200140	A/swine/California/A01459047/2015	H1N1	Negative
EPI_ISL_196006	A/swine/Mexico/9557133/2013	H1N1	Negative
EPI_ISL_129511	A/swine/Finnistere/800/2004	H1N1	Negative
EPI_ISL_169576	A/swine/Hunan/951/2013	H1N1	Negative
EPI_ISL_97115	A/swine/Minnesota/A01049893/2011	H1N1	Negative
EPI_ISL_63616	A/swine/Minnesota/07002083/2007	H1N1	Negative
EPI_ISL_88258	A/swine/Hong_Kong/48/1977	H1N1	Negative
EPI_ISL_151778	A/swine/Illinois/A01227426/2013	H1N1	Negative
EPI_ISL_150923	A/swine/North_Carolina/02403/2008	H1N1	Negative
EPI_ISL_281958	A/swine/Minnesota/MT_13_01_S43/2013	H1N1	Negative
EPI_ISL_129528	A/swine/Italy/289171/2003	H1N1	Negative
EPI_ISL_151213	A/swine/Nebraska/00178/2003	H1N1	Negative
EPI_ISL_195155	A/swine/France/22-120340/2012	H1N1	Negative
EPI_ISL_243807	A/swine/Indiana/A01812247/2016	H1N1	Negative
EPI_ISL_272824	A/swine/Minnesota/A02214666/2017	H1N1	Negative
EPI_ISL_225564	A/swine/Minnesota/A01774384/2016	H1N1	Negative
EPI_ISL_195015	A/swine/Italy/302593/2010	H1N1	Negative
EPI_ISL_170579	A/swine/Germany/2/1981	H1N1	Negative
EPI_ISL_260803	A/swine/Iowa/A01672342/2017	H1N1	Negative
EPI_ISL_282135	A/swine/Czech_Republic/13240/2017_(H1N1)	H1N1	Negative
EPI_ISL_95802	A/swine/North_Carolina/18161/2002	H1N1	Negative
EPI_ISL_127793	A/swine/Michigan/A01202708/2011	H1N1	Negative
EPI_ISL_243783	A/swine/North_Carolina/A01781336/2016	H1N1	Negative
EPI_ISL_195142	A/swine/Belgium/Minderhout-348/2012	H1N1	Negative
EPI_ISL_195156	A/swine/France/29-120326/2012	H1N1	Negative
EPI_ISL_29419	A/swine/France/WVL4/1985	H1N1	Negative
EPI_ISL_195058	A/swine/Spain/23998/2011	H1N1	Negative

EPI_ISL_278868	A/swine/Italy/133457-3/2014	H1N1	Negative
EPI_ISL_151289	A/swine/South_Carolina/01414/2006	H1N1	Negative
EPI_ISL_151284	A/swine/Texas/00867/2005	H1N1	Negative
EPI_ISL_29420	A/swine/Belgium/WVL5/1989	H1N1	Negative
EPI_ISL_151208	A/swine/Saskatchewan/01974/2008	H1N1	Negative
EPI_ISL_264208	A/swine/Ohio/A02214229/2017	H1N1	Negative
EPI_ISL_253727	A/swine/Indiana/A01671620/2016	H1N1	Negative
EPI_ISL_221727	A/swine/North_Carolina/A01733980/2016	H1N1	Negative
EPI_ISL_188901	A/swine/Ohio/A01847657/2015	H1N1	Negative
EPI_ISL_151285	A/swine/Oklahoma/00790/2005	H1N1	Negative
EPI_ISL_150872	A/swine/Texas/01657/2007	H1N1	Negative
EPI_ISL_132360	A/swine/Iowa/A01203904/2012	H1N1	Negative
EPI_ISL_166533	A/swine/Guangdong/SS1/2012	H1N1	Negative
EPI_ISL_195040	A/swine/Netherlands/Dalfsen-12/2012	H1N1	Negative
EPI_ISL_130041	A/swine/Nebraska/A01327160/2012	H1N1	Negative
EPI_ISL_88348	A/swine/Hong_Kong/841/2003	H1N1	Negative
EPI_ISL_150847	A/swine/Virginia/01359/2006	H1N1	Negative
EPI_ISL_151270	A/swine/Oklahoma/01139/2006	H1N1	Negative
EPI_ISL_203151	A/swine/Indiana/A01812000/2015	H1N1	Negative
EPI_ISL_195085	A/swine/Germany/Reinberg-IDT14457-1/2012	H1N1	Negative
EPI_ISL_225549	A/swine/North_Carolina/A01774921/2016	H1N1	Negative
EPI_ISL_71595	A/swine/Germany-BB/siv-leipz11308/09	H1N1	Negative
EPI_ISL_129559	A/swine/Gent/112/2007	H1N1	Negative
EPI_ISL_195024	A/swine/Netherlands/Kootwijkerbroek-51/2012	H1N1	Negative
EPI_ISL_195106	A/swine/Italy/28762-3/2013	H1N1	Negative
EPI_ISL_96922	A/swine/Minnesota/02905/2009	H1N1	Negative
EPI_ISL_130124	A/swine/Nebraska/A01301493/2012	H1N1	Negative
EPI_ISL_130006	A/swine/Minnesota/A01241397/2012	H1N1	Negative
EPI_ISL_256370	A/swine/Jiangsu/POS759/2013	H1N1	Negative
EPI_ISL_258617	A/swine/Iowa/A01672824/2017	H1N1	Negative
EPI_ISL_195112	A/swine/Poland/16315/2011	H1N1	Negative
EPI_ISL_106287	A/swine/North_Carolina/3793/2008	H1N1	Negative
EPI_ISL_132318	A/swine/Indiana/A01260029/2012	H1N1	Negative
EPI_ISL_71594	A/swine/Germany-SH/R1737/09	H1N1	Negative
EPI_ISL_299404	A/swine/Kansas/A01378038/2017	H1N1	Negative
EPI_ISL_151211	A/swine/North_Carolina/SG1307/2008	H1N1	Negative
EPI_ISL_237038	A/swine/Iowa/A01781047/2016	H1N1	Negative
EPI_ISL_195025	A/swine/Netherlands/Groenlo-37/2012	H1N1	Negative
EPI_ISL_96940	A/swine/Illinois/02385/2008	H1N1	Negative
EPI_ISL_88255	A/swine/Hong_Kong/45/1977	H1N1	Negative
EPI_ISL_207041	A/swine/Manitoba/D0172/2012	H1N1	Negative
EPI_ISL_29308	A/swine/Hungary/19774/2006	H1N1	Negative
EPI_ISL_129460	A/swine/England/117316/1986	H1N1	Negative
EPI_ISL_297123	A/swine/Nebraska/A02136881/2017	H1N1	Negative
EPI_ISL_129579	A/swine/Italy/29313/2006	H1N1	Negative
EPI_ISL_154632	A/swine/North_Carolina/A01290892/2013	H1N1	Negative
EPI_ISL_117012	A/swine/Oklahoma/A01134906/2011	H1N1	Negative
EPI_ISL_98867	A/swine/Mississippi/A01202059/2011	H1N1	Negative
EPI_ISL_129487	A/swine/England/425742/1994	H1N1	Negative
EPI_ISL_155700	A/swine/South_Dakota/SJ1319788/2013	H1N1	Negative
EPI_ISL_195181	A/swine/Netherlands/Mill-CVI2617A/2012	H1N1	Negative
EPI_ISL_150673	A/swine/Oklahoma/A01290605/2013	H1N1	Negative
EPI_ISL_278866	A/swine/Italy/302661/2014	H1N1	Negative
EPI_ISL_151196	A/swine/Illinois/02166/2008	H1N1	Negative
EPI_ISL_132964	A/swine/Nebraska/A01277241/2012	H1N1	Negative
EPI_ISL_29421	A/swine/Spain/WVL6/1991	H1N1	Negative
EPI_ISL_143152	A/swine/Illinois/A01158960/2013	H1N1	Negative
EPI_ISL_151254	A/swine/Texas/00244/2004	H1N1	Negative

EPI_ISL_150940	A/swine/Illinois/02757/2009	H1N1	Negative
EPI_ISL_138219	A/swine/Indiana/A01260209/2013	H1N1	Negative
EPI_ISL_137557	A/swine/North_Carolina/A01444883/2013	H1N1	Negative
EPI_ISL_143149	A/swine/Illinois/A01203923/2012	H1N1	Negative
EPI_ISL_278867	A/swine/Italy/304621/2014	H1N1	Negative
EPI_ISL_243772	A/swine/Indiana/15TOSU0860/2015	H1N1	Negative
EPI_ISL_124332	A/swine/Minnesota/A01076180/2009	H1N1	Negative
EPI_ISL_29422	A/swine/England/WVL7/1992	H1N1	Negative
EPI_ISL_222096	A/swine/Oklahoma/A01732565/2016	H1N1	Negative
EPI_ISL_168335	A/swine/Indiana/13TOSU4293/2013	H1N1	Negative
EPI_ISL_196021	A/swine/Mexico/9800323/2013	H1N1	Negative
EPI_ISL_195105	A/swine/France/56-110525/2010	H1N1	Negative
EPI_ISL_97090	A/swine/Missouri/A01049487/2011	H1N1	Negative
EPI_ISL_266543	A/swine/Illinois/A02214663/2017	H1N1	Negative
EPI_ISL_116993	A/swine/Illinois/A01134216/2011	H1N1	Negative
EPI_ISL_264228	A/swine/Iowa/A01667089/2017	H1N1	Negative
EPI_ISL_129521	A/swine/Italy/1513-1/1998	H1N1	Negative
EPI_ISL_129499	A/swine/England/68327/1998	H1N1	Negative
EPI_ISL_138860	A/swine/Illinois/A01445653/2013	H1N1	Negative
EPI_ISL_253702	A/swine/Illinois/A01671425/2016	H1N1	Negative
EPI_ISL_88249	A/swine/Hong_Kong/39/1977	H1N1	Negative
EPI_ISL_129458	A/swine/England/1131/2004	H1N1	Negative
EPI_ISL_98868	A/swine/Iowa/A01202056/2011	H1N1	Negative
EPI_ISL_79277	A/swine/Germany-NI/R3394/09	H1N1	Negative
EPI_ISL_237036	A/swine/Iowa/A01781050/2016	H1N1	Negative
EPI_ISL_88347	A/swine/Hong_Kong/638/2003	H1N1	Negative
EPI_ISL_252718	A/swine/North_Carolina/A01776362/2016	H1N1	Negative
EPI_ISL_129518	A/swine/OMS/2112/1995	H1N1	Negative
EPI_ISL_127784	A/swine/Indiana/A01202622/2011	H1N1	Negative
EPI_ISL_278870	A/swine/Italy/138746/2014	H1N1	Negative
EPI_ISL_92022	A/swine/Minnesota/01076/2006	H1N1	Negative
EPI_ISL_129479	A/swine/England/35320/1999	H1N1	Negative
EPI_ISL_88253	A/swine/Hong_Kong/43/1977	H1N1	Negative
EPI_ISL_258625	A/swine/Indiana/A01672825/2017	H1N1	Negative
EPI_ISL_278829	A/swine/Italy/154890-2/2015	H1N1	Negative
EPI_ISL_203136	A/swine/Indiana/A01945792/2015	H1N1	Negative
EPI_ISL_297126	A/swine/Oklahoma/A02135952/2017	H1N1	Negative
EPI_ISL_282403	A/swine/Illinois/A02219783/2017	H1N1	Negative
EPI_ISL_181274	A/swine/North_Carolina/A01476862/2014	H1N1	Negative
EPI_ISL_150705	A/swine/Tennessee/00264/2004	H1N1	Negative
EPI_ISL_13578	A/swine/Haseluenne/IDT2617/03	H1N1	Negative
EPI_ISL_151002	A/swine/Iowa/SG1401/2011	H1N1	Negative
EPI_ISL_195146	A/swine/Belgium/Heist-op-den-Berg-363/2012	H1N1	Negative
EPI_ISL_116143	A/swine/Illinois/A01203226/2012	H1N1	Negative
EPI_ISL_29423	A/swine/France/WVL8/1992	H1N1	Negative
EPI_ISL_161111	A/swine/Indiana/13TOSU1043/2013	H1N1	Negative
EPI_ISL_87552	A/swine/Iowa/A01057153/2010	H1N1	Negative
EPI_ISL_203117	A/swine/North_Carolina/A01795443/2015	H1N1	Negative
EPI_ISL_151615	A/swine/Colorado/SG1185/2004	H1N1	Negative
EPI_ISL_5287	A/swine/Spain/51915/2003	H1N1	Negative
EPI_ISL_253918	A/swine/Nebraska/A01671947/2016	H1N1	Negative
EPI_ISL_156554	A/swine/Illinois/A01427261/2013	H1N1	Negative
EPI_ISL_161154	A/swine/Indiana/13TOSU0832/2013	H1N1	Negative
EPI_ISL_295526	A/swine/Kansas/A01378037/2017	H1N1	Negative
EPI_ISL_151259	A/swine/North_Carolina/00371/2004	H1N1	Negative
EPI_ISL_128168	A/swine/Mexico/Ver29/2010	H1N1	Negative
EPI_ISL_278888	A/swine/Italy/359202/2013	H1N1	Negative
EPI_ISL_278908	A/swine/Italy/280201/2013	H1N1	Negative

EPI_ISL_296710	A/swine/Wisconsin/A01104100/2017	H1N1	Negative
EPI_ISL_88248	A/swine/Hong_Kong/38/1977	H1N1	Negative
EPI_ISL_246023	A/swine/Missouri/A01668216/2016	H1N1	Negative
EPI_ISL_296673	A/swine/Minnesota/A02223472/2017	H1N1	Negative
EPI_ISL_129522	A/swine/Italy/151672-3/2003	H1N1	Negative
EPI_ISL_116936	A/swine/Illinois/A01047984/2011	H1N1	Negative
EPI_ISL_88276	A/swine/Hong_Kong/70/1977	H1N1	Negative
EPI_ISL_260815	A/swine/Kansas/A01378019/2017	H1N1	Negative
EPI_ISL_93354	A/swine/Iowa/A01049244/2010	H1N1	Negative
EPI_ISL_199470	A/swine/Belgium/1/98	H1N1	Negative
EPI_ISL_129447	A/swine/Bieganow/1/2001	H1N1	Negative
EPI_ISL_195029	A/swine/Belgium/Meeuwen-231/2011	H1N1	Negative
EPI_ISL_195038	A/swine/Belgium/Wingene-455/2011	H1N1	Negative
EPI_ISL_13917	A/swine/Chachoengsao/NIAH587/2005	H1N1	Negative
EPI_ISL_7790	A/swine/Tennessee/23/1976	H1N1	Negative
EPI_ISL_5203	A/Swine/Spain/50047/2003	H1N1	Negative
EPI_ISL_97126	A/swine/Iowa/A01049949/2011	H1N1	Negative
EPI_ISL_4038	A/swine/Saskatchewan/18789/02	H1N1	Negative
EPI_ISL_100196	A/swine/Ohio/FAH2-1/2008	H1N1	Negative
EPI_ISL_9880	A/swine/Ontario/53518/03	H1N1	Negative
EPI_ISL_133715	A/swine/Minnesota/A01244318/2012	H1N1	Negative
EPI_ISL_150924	A/swine/North_Carolina/SG1313/2008	H1N1	Negative
EPI_ISL_124407	A/swine/Illinois/A01047982/2011	H1N1	Negative
EPI_ISL_195021	A/swine/Netherlands/Groelnlo-186/2011	H1N1	Negative
EPI_ISL_151282	A/swine/Minnesota/00401/2004	H1N1	Negative
EPI_ISL_278876	A/swine/Italy/96500/2014	H1N1	Negative
EPI_ISL_129457	A/swine/England/1093/2005	H1N1	Negative
EPI_ISL_129501	A/swine/England/745769/1995	H1N1	Negative
EPI_ISL_151263	A/swine/North_Carolina/SG1463/2004	H1N1	Negative
EPI_ISL_129502	A/swine/England/771270/1995	H1N1	Negative
EPI_ISL_243759	A/swine/Indiana/15TOSU1184/2015	H1N1	Negative
EPI_ISL_195145	A/swine/Netherlands/Hoogeloon-167C/2012	H1N1	Negative
EPI_ISL_176705	A/Utah/10/2015	H3N2	Positive
EPI_ISL_235233	A/Moscow/1514A00504942N/2012	H3N2	Positive
EPI_ISL_302484	A/Cardiff/7495/2017	H3N2	Positive
EPI_ISL_263254	A/Bage/LACENRS-2050/2013	H3N2	Positive
EPI_ISL_329971	A/Iowa/38/2017	H3N2	Positive
EPI_ISL_108091	A/Boston/DOA17/2011	H3N2	Positive
EPI_ISL_114014	A/Bilthoven/5029/1976	H3N2	Positive
EPI_ISL_114019	A/Rotterdam/5828/1977	H3N2	Positive
EPI_ISL_5525	A/New_York/707/1994	H3N2	Positive
EPI_ISL_129188	A/Michigan/20/2012	H3N2	Positive
EPI_ISL_129176	A/Maryland/25/2012	H3N2	Positive
EPI_ISL_322180	A/Caerphilly/1813/2018	H3N2	Positive
EPI_ISL_118112	A/HaNoi/N062/2007	H3N2	Positive
EPI_ISL_5486	A/New_York/657/1994	H3N2	Positive
EPI_ISL_6747	A/New_York/248/1999	H3N2	Positive
EPI_ISL_312866	A/Minnesota/38/2018	H3N2	Positive
EPI_ISL_129621	A/Michigan/23/2012	H3N2	Positive
EPI_ISL_120372	A/Malaysia/26682/2004	H3N2	Positive
EPI_ISL_127606	A/reassortant/X-145	H3N2	Positive
EPI_ISL_5555	A/New_York/779/1993	H3N2	Positive
EPI_ISL_5381	A/New_York/702/1995	H3N2	Positive
EPI_ISL_5297	A/New_York/611/1995	H3N2	Positive
EPI_ISL_99793	A/Iowa/09/2011	H3N2	Positive
EPI_ISL_344895	A/Bretagne/1296/2019	H3N2	Positive
EPI_ISL_62513	A/New_York/3280/2009	H3N2	Positive
EPI_ISL_114200	A/Bilthoven/10684/1982	H3N2	Positive

EPI_ISL_76123	A/California/VRDL144/2009	H3N2	Positive
EPI_ISL_5307	A/New_York/637/1996	H3N2	Positive
EPI_ISL_294265	A/Maryland/50/2017	H3N2	Positive
EPI_ISL_6481	A/Queensland/47/2004	H3N2	Positive
EPI_ISL_294273	A/Maryland/60/2017	H3N2	Positive
EPI_ISL_246097	A/Hong_Kong/01/1968	H3N2	Positive
EPI_ISL_66559	A/Victoria/208/2009	H3N2	Positive
EPI_ISL_9030	A/Memphis/2/78	H3N2	Positive
EPI_ISL_8676	A/Hong_Kong/6/1972	H3N2	Positive
EPI_ISL_124928	A/Leningrad/360/1986	H3N2	Positive
EPI_ISL_8191	A/New_York/382/2005	H3N2	Positive
EPI_ISL_8638	A/New_York/431/2000	H3N2	Positive
EPI_ISL_8696	A/Hong_Kong/14/1992	H3N2	Positive
EPI_ISL_115460	A/Finland/339/1995	H3N2	Positive
EPI_ISL_7153	A/Auckland/616/2005	H3N2	Positive
EPI_ISL_5380	A/New_York/698/1995	H3N2	Positive
EPI_ISL_9104	A/Whanganui/129/2004	H3N2	Positive
EPI_ISL_327396	A/Cowbridge/8371/2018	H3N2	Positive
EPI_ISL_114610	A/Beijing/32/1992	H3N2	Positive
EPI_ISL_9252	A/Memphis/3/1980	H3N2	Positive
EPI_ISL_6862	A/South_Australia/80/2000	H3N2	Positive
EPI_ISL_5663	A/New_York/772/1993	H3N2	Positive
EPI_ISL_309467	A/Penarth/8264/2018	H3N2	Positive
EPI_ISL_235308	A/Puno/IPE00792/2011	H3N2	Positive
EPI_ISL_235305	A/Masaya/INI00224/2011	H3N2	Positive
EPI_ISL_9216	A/Nanjing/28/1984	H3N2	Positive
EPI_ISL_90550	A/California/NHRC0009/2005	H3N2	Positive
EPI_ISL_62790	A/Boston/40/2008	H3N2	Positive
EPI_ISL_114655	A/Houston/56829/1992	H3N2	Positive
EPI_ISL_327722	A/Abertillery/1971/2018	H3N2	Positive
EPI_ISL_120410	A/Malaysia/1959864/2008	H3N2	Positive
EPI_ISL_33841	A/Siena/3/1991	H3N2	Positive
EPI_ISL_8639	A/New_York/433/2000	H3N2	Positive
EPI_ISL_6671	A/New_York/18/2003	H3N2	Positive
EPI_ISL_8972	A/Nanjing/13/1980	H3N2	Positive
EPI_ISL_167928	A/Hawaii/35/2014	H3N2	Positive
EPI_ISL_152841	A/Peru/PER300/2010	H3N2	Positive
EPI_ISL_119710	A/California/22/2012	H3N2	Positive
EPI_ISL_189019	A/Nicaragua/AGA2-30/2011	H3N2	Positive
EPI_ISL_5481	A/New_York/764/1993	H3N2	Positive
EPI_ISL_5664	A/New_York/773/1993	H3N2	Positive
EPI_ISL_232044	A/Ohio/27/2016	H3N2	Positive
EPI_ISL_5306	A/New_York/634/1995	H3N2	Positive
EPI_ISL_94612	A/Heilongjiang-Xiangyang/1134/2011	H3N2	Positive
EPI_ISL_342242	A/Kansas/07/2019	H3N2	Positive
EPI_ISL_9029	A/Hong_Kong/46/1971	H3N2	Positive
EPI_ISL_9208	A/Memphis/2/1977	H3N2	Positive
EPI_ISL_127589	A/Johannesburg/33/1994	H3N2	Positive
EPI_ISL_294278	A/Maryland/65/2017	H3N2	Positive
EPI_ISL_6978	A/Albany/2/1970	H3N2	Positive
EPI_ISL_77612	A/California/VRDL328/2009	H3N2	Positive
EPI_ISL_89636	A/New_York/3750/2009	H3N2	Positive
EPI_ISL_114656	A/Houston/56941/1992	H3N2	Positive
EPI_ISL_189225	A/Houston/JMM_98/2012	H3N2	Positive
EPI_ISL_120417	A/Malaysia/12106/1997	H3N2	Positive
EPI_ISL_263140	A/Canoas/LACENRS-1660/2014	H3N2	Positive
EPI_ISL_321574	A/Abertillery/5073/2018	H3N2	Positive
EPI_ISL_5028	A/New_York/92/2002	H3N2	Positive

EPI_ISL_5438	A/New_York/685/1995	H3N2	Positive
EPI_ISL_5437	A/New_York/684/1995	H3N2	Positive
EPI_ISL_5305	A/New_York/631/1996	H3N2	Positive
EPI_ISL_160265	A/Boston/YGA_00015/2012	H3N2	Positive
EPI_ISL_6157	A/Dunedin/1/2002	H3N2	Positive
EPI_ISL_5406	A/New_York/693/1995	H3N2	Positive
EPI_ISL_342951	A/Bourgogne/1094/2019	H3N2	Positive
EPI_ISL_173050	A/Myanmar/13M118/2013	H3N2	Positive
EPI_ISL_7841	A/Oregon/UR06-0450/2007	H3N2	Positive
EPI_ISL_5524	A/New_York/705/1994	H3N2	Positive
EPI_ISL_120364	A/Malaysia/23606/2002	H3N2	Positive
EPI_ISL_93668	A/Mexico_City/WRAIR3568N/2010	H3N2	Positive
EPI_ISL_127601	A/California/32/1999	H3N2	Positive
EPI_ISL_234481	A/Ohio/35/2016	H3N2	Positive
EPI_ISL_115041	A/Enschede/5458/1993	H3N2	Positive
EPI_ISL_170377	A/Wisconsin/24/2014	H3N2	Positive
EPI_ISL_152597	A/Peru/PER230/2010	H3N2	Positive
EPI_ISL_5377	A/New_York/695/1995	H3N2	Positive
EPI_ISL_263256	A/Santa_Maria/LACENRS-231/2011	H3N2	Positive
EPI_ISL_5378	A/New_York/696/1994	H3N2	Positive
EPI_ISL_6979	A/Albany/20/1974	H3N2	Positive
EPI_ISL_5436	A/New_York/652/1996	H3N2	Positive
EPI_ISL_5006	A/New_York/110/2002	H3N2	Positive
EPI_ISL_5164	A/Canterbury/43/2001	H3N2	Positive
EPI_ISL_114503	A/Geneva/5366/1991	H3N2	Positive
EPI_ISL_173307	A/Vietnam/13V_H3-9/2012	H3N2	Positive
EPI_ISL_5478	A/New_York/761/1993	H3N2	Positive
EPI_ISL_77852	A/California/VRDL393/2009	H3N2	Positive
EPI_ISL_27630	A/Ohio/UR07-0089/2008	H3N2	Positive
EPI_ISL_29471	A/Siena/3/1995	H3N2	Positive
EPI_ISL_115303	A/Madrid/G116/1993	H3N2	Positive
EPI_ISL_29655	A/Hong_Kong/HKU14/2004	H3N2	Positive
EPI_ISL_5570	A/New_York/673/1995	H3N2	Positive
EPI_ISL_114018	A/Bilthoven/3895/1977	H3N2	Positive
EPI_ISL_6982	A/Albany/6/1970	H3N2	Positive
EPI_ISL_152265	A/Peru/PER071/2010	H3N2	Positive
EPI_ISL_188811	A/England/575/2014	H3N2	Positive
EPI_ISL_329981	A/North_Dakota/19/2017	H3N2	Positive
EPI_ISL_120353	A/Malaysia/12990/1997	H3N2	Positive
EPI_ISL_107487	A/England/484/2003	H3N2	Positive
EPI_ISL_9032	A/Memphis/18/1978	H3N2	Positive
EPI_ISL_115510	A/Hong_Kong/49/1995	H3N2	Positive
EPI_ISL_5176	A/New_York/574/1996	H3N2	Positive
EPI_ISL_307665	A/Newport/8612/2018	H3N2	Positive
EPI_ISL_83701	A/Minnesota/11/2010	H3N2	Positive
EPI_ISL_9040	A/New_York/535/1998	H3N2	Positive
EPI_ISL_22616	A/Hong_Kong/1-9-MA21-3/1968	H3N2	Positive
EPI_ISL_9287	A/Canterbury/56/2000	H3N2	Positive
EPI_ISL_8200	A/Memphis/59/1999	H3N2	Positive
EPI_ISL_127613	A/Wisconsin/03/2007	H3N2	Positive
EPI_ISL_8176	A/New_York/336/1999	H3N2	Positive
EPI_ISL_321587	A/Aberdare/1604/2018	H3N2	Positive
EPI_ISL_9209	A/Memphis/4/1977	H3N2	Positive
EPI_ISL_113920	A/Bilthoven/9459/1974	H3N2	Positive
EPI_ISL_129408	A/Michigan/19/2012	H3N2	Positive
EPI_ISL_6191	A/Western_Australia/43/2003	H3N2	Positive
EPI_ISL_8954	A/Hong_Kong/1/1982	H3N2	Positive
EPI_ISL_142987	A/Singapore/H2010.797/2010	H3N2	Positive

EPI_ISL_114657	A/Madrid/G58/1992	H3N2	Positive
EPI_ISL_327399	A/Cardiff/8857/2018	H3N2	Positive
EPI_ISL_5009	A/New_York/26/2003	H3N2	Positive
EPI_ISL_115463	A/Hong_Kong/3/1995	H3N2	Positive
EPI_ISL_8587	A/Memphis/109/1972	H3N2	Positive
EPI_ISL_90497	A/Texas/NHRC0001/2005	H3N2	Positive
EPI_ISL_234473	A/Michigan/90/2016	H3N2	Positive
EPI_ISL_8993	A/New_York/467/2004	H3N2	Positive
EPI_ISL_6858	A/Wellington/21/2000	H3N2	Positive
EPI_ISL_195625	A/Quebec/26-281106/2006	H3N2	Positive
EPI_ISL_117091	A/HaNoi/BM766/2003	H3N2	Positive
EPI_ISL_115301	A/Madrid/G101/1993	H3N2	Positive
EPI_ISL_7268	A/Albany/1/1970	H3N2	Positive
EPI_ISL_114813	A/Paris/597/1991	H3N2	Positive
EPI_ISL_152819	A/Peru/PER357/2010	H3N2	Positive
EPI_ISL_148000	A/Boston/DOA2-155/2012	H3N2	Positive
EPI_ISL_95264	A/Indiana/08/2011	H3N2	Positive
EPI_ISL_8193	A/New_York/387/2004	H3N2	Positive
EPI_ISL_25042	A/Siena/10/1990	H3N2	Positive
EPI_ISL_267075	A/Texas/105/2017	H3N2	Positive
EPI_ISL_115462	A/Geneva/A9509/1995	H3N2	Positive
EPI_ISL_5444	A/New_York/715/1994	H3N2	Positive
EPI_ISL_114457	A/Wellington/3/1990	H3N2	Positive
EPI_ISL_5049	A/New_York/149/1999	H3N2	Positive
EPI_ISL_99417	A/Indiana/10/2011	H3N2	Positive
EPI_ISL_115770	A/United_Kingdom/261/1991	H3N2	Positive
EPI_ISL_7269	A/Albany/3/1970	H3N2	Positive
EPI_ISL_114202	A/Phillipines/2/1982	H3N2	Positive
EPI_ISL_115565	A/Hong_Kong/434/1996	H3N2	Positive
EPI_ISL_202678	A/Macau/611810/2015	H3N2	Positive
EPI_ISL_5514	A/New_York/655/1994	H3N2	Positive
EPI_ISL_113969	A/Bilthoven/628/1976	H3N2	Positive
EPI_ISL_117209	A/HaNoi/BM769/2004	H3N2	Positive
EPI_ISL_114348	A/Netherlands/650/1989	H3N2	Positive
EPI_ISL_321565	A/Anglesey/5245/2018	H3N2	Positive
EPI_ISL_139924	A/Alaska/02/2013	H3N2	Positive
EPI_ISL_327397	A/Cardiff/8462/2018	H3N2	Positive
EPI_ISL_94617	A/Liaoning-Haizhou/1118/2011	H3N2	Positive
EPI_ISL_114250	A/Wellington/4/1985	H3N2	Positive
EPI_ISL_321571	A/Wrexham/9243/2018	H3N2	Positive
EPI_ISL_117952	A/TayNguyen/TN380/2005	H3N2	Positive
EPI_ISL_294287	A/Maryland/75/2017	H3N2	Positive
EPI_ISL_90470	A/Missouri/NHRC0001/2000	H3N2	Positive
EPI_ISL_257203	A/Saudi_Arabia/20/2015	H3N2	Positive
EPI_ISL_321570	A/Wrexham/9057/2018	H3N2	Positive
EPI_ISL_274392	A/London/16U828652-10_S10_L001/2016	H3N2	Positive
EPI_ISL_5157	A/New_York/579/1997	H3N2	Positive
EPI_ISL_114397	A/Singapore/35/1989	H3N2	Positive
EPI_ISL_5372	A/Memphis/5/1986	H3N2	Positive
EPI_ISL_6216	A/Western_Australia/73/2005	H3N2	Positive
EPI_ISL_151	A/Hong_Kong/1/68	H3N2	Positive
EPI_ISL_69818	A/Victoria/1968	H3N2	Positive
EPI_ISL_9039	A/Nanjing/49/1977	H3N2	Positive
EPI_ISL_9034	A/Memphis/137/1976	H3N2	Positive
EPI_ISL_189083	A/Nicaragua/AGA2-64/2011	H3N2	Positive
EPI_ISL_114759	A/Paris/424/1992	H3N2	Positive
EPI_ISL_8692	A/New_York/485/2003	H3N2	Positive
EPI_ISL_127587	A/Shanghai/11/1987	H3N2	Positive

EPI_ISL_107473	A/England/525/2003	H3N2	Positive
EPI_ISL_118214	A/HaNoi/Q148/2007	H3N2	Positive
EPI_ISL_5289	A/New_York/572/1996	H3N2	Positive
EPI_ISL_115040	A/Akita/4/1993	H3N2	Positive
EPI_ISL_6665	A/New_York/189/1999	H3N2	Positive
EPI_ISL_279385	A/Nicaragua/6585_04/2014	H3N2	Positive
EPI_ISL_114400	A/Wellington/5/1989	H3N2	Positive
EPI_ISL_5395	A/Memphis/12/1985	H3N2	Positive
EPI_ISL_107296	A/England/754/2003	H3N2	Positive
EPI_ISL_5205	A/New_York/593/1996	H3N2	Positive
EPI_ISL_83687	A/Jiangxi-Donghu/1682/2010	H3N2	Positive
EPI_ISL_8104	A/Udorn/307/1972	H3N2	Positive
EPI_ISL_6763	A/Queensland/53/2005	H3N2	Positive
EPI_ISL_158671	A/Tennessee/F2078/2011	H3N2	Positive
EPI_ISL_9038	A/Nanjing/2/1982	H3N2	Positive
EPI_ISL_9285	A/Memphis/15/1988	H3N2	Positive
EPI_ISL_145695	A/Singapore/78L/2007	H3N2	Positive
EPI_ISL_114816	A/South_Austalia/27/1992	H3N2	Positive
EPI_ISL_93640	A/District_of_Columbia/WRAIR0301/2010	H3N2	Positive
EPI_ISL_115408	A/Scotland/142/1993	H3N2	Positive
EPI_ISL_5573	A/New_York/690/1995	H3N2	Positive
EPI_ISL_97361	A/Maine/06/2011	H3N2	Positive
EPI_ISL_90491	A/Missouri/NHRC0001/2006	H3N2	Positive
EPI_ISL_6705	A/New_York/66/2003	H3N2	Positive
EPI_ISL_9063	A/Canterbury/390/2003	H3N2	Positive
EPI_ISL_177688	A/Karlstad/3/2015	H3N2	Positive
EPI_ISL_5670	A/New_York/788/1993	H3N2	Positive
EPI_ISL_115771	A/Brisbane/8/1996	H3N2	Positive
EPI_ISL_152438	A/Peru/PER162/2010	H3N2	Positive
EPI_ISL_5624	A/New_York/688/1994	H3N2	Positive
EPI_ISL_205392	A/Thailand/CU-CB166/2014	H3N2	Positive
EPI_ISL_6166	A/Wellington/6/2005	H3N2	Positive
EPI_ISL_327732	A/Cardiff/1973/2018	H3N2	Positive
EPI_ISL_120334	A/Malaysia/07145/1995	H3N2	Positive
EPI_ISL_5451	A/New_York/728/1994	H3N2	Positive
EPI_ISL_152684	A/Peru/PER281/2011	H3N2	Positive
EPI_ISL_114455	A/Suita/1/1990	H3N2	Positive
EPI_ISL_309457	A/Neath/9065/2018	H3N2	Positive
EPI_ISL_113919	A/Bilthoven/7398/1974	H3N2	Positive
EPI_ISL_5401	A/New_York/669/1995	H3N2	Positive
EPI_ISL_5309	A/New_York/648/1995	H3N2	Positive
EPI_ISL_294276	A/Maryland/63/2017	H3N2	Positive
EPI_ISL_107470	A/England/788/2003	H3N2	Positive
EPI_ISL_321568	A/Caernarfon/8500/2018	H3N2	Positive
EPI_ISL_5390	A/New_York/624/1996	H3N2	Positive
EPI_ISL_5711	A/Wellington/1/2005	H3N2	Positive
EPI_ISL_117522	A/HaNoi/BM544/2004	H3N2	Positive
EPI_ISL_5076	A/New_York/171/1999	H3N2	Positive
EPI_ISL_125872	A/Philippines/2/1982	H3N2	Positive
EPI_ISL_114246	A/Guildford/V728/1985	H3N2	Positive
EPI_ISL_29662	A/Hong_Kong/HKU33/2004	H3N2	Positive
EPI_ISL_152272	A/Peru/PER078/2012	H3N2	Positive
EPI_ISL_114249	A/Stockholm/10/1985	H3N2	Positive
EPI_ISL_5216	A/New_York/562/1996	H3N2	Positive
EPI_ISL_302456	A/Carmarthen/5313/2017	H3N2	Positive
EPI_ISL_176862	A/Okinawa/14T006/2015	H3N2	Positive
EPI_ISL_115566	A/Lyon/1781/1996	H3N2	Positive
EPI_ISL_94642	A/Heilongjiang-Taoshan/147/2011	H3N2	Positive

EPI_ISL_5551	A/New_York/771/1993	H3N2	Positive
EPI_ISL_6659	A/New_York/59/2003	H3N2	Positive
EPI_ISL_20992	A/New_York/928/2006	H3N2	Positive
EPI_ISL_5300	A/New_York/618/1995	H3N2	Positive
EPI_ISL_29483	A/Hong_Kong/HKU16/2004	H3N2	Positive
EPI_ISL_110692	A/Netherlands/217/2003	H3N2	Positive
EPI_ISL_5482	A/New_York/765/1993	H3N2	Positive
EPI_ISL_321569	A/Anglesey/8900/2018	H3N2	Positive
EPI_ISL_114293	A/Colorado/2/1986	H3N2	Positive
EPI_ISL_195626	A/Quebec/26-191206/2006	H3N2	Positive
EPI_ISL_9283	A/Memphis/11/1986	H3N2	Positive
EPI_ISL_9095	A/Canterbury/437/2003	H3N2	Positive
EPI_ISL_8198	A/Memphis/66/1986	H3N2	Positive
EPI_ISL_118368	A/HaNoi/TX072/2007	H3N2	Positive
EPI_ISL_143054	A/Singapore/H2010.797/2010	H3N2	Positive
EPI_ISL_118320	A/HaNoi/TX022/2007	H3N2	Positive
EPI_ISL_274831	A/London/16U928199-70_S22_L001/2016	H3N2	Positive
EPI_ISL_8664	A/New_York/439/2000	H3N2	Positive
EPI_ISL_6849	A/Albany/19/1968	H3N2	Positive
EPI_ISL_114196	A/Rotterdam/8179/1977	H3N2	Positive
EPI_ISL_108088	A/Boston/DOA06/2011	H3N2	Positive
EPI_ISL_5448	A/New_York/721/1994	H3N2	Positive
EPI_ISL_149718	A/Stockholm/26/2011	H3N2	Positive
EPI_ISL_195622	A/Quebec/26-310106/2006	H3N2	Positive
EPI_ISL_195645	A/Michigan/39/2015	H3N2	Positive
EPI_ISL_9145	A/Memphis/19/1978	H3N2	Positive
EPI_ISL_8670	A/Hong_Kong/6/1985	H3N2	Positive
EPI_ISL_9098	A/Canterbury/440/2003	H3N2	Positive
EPI_ISL_98696	A/Hong_Kong/HKU119/2004	H3N2	Positive
EPI_ISL_124885	A/Bilthoven/16190/1968	H3N2	Positive
EPI_ISL_120345	A/Malaysia/11473/1996	H3N2	Positive
EPI_ISL_239893	A/Ontario/RV3236/2016	H3N2	Positive
EPI_ISL_80059	A/California/VRDL386/2009	H3N2	Positive
EPI_ISL_115699	A/Netherlands/233/1982	H3N2	Positive
EPI_ISL_8974	A/Memphis/2/1971	H3N2	Positive
EPI_ISL_132408	A/Mexico/InDRE2112/2005	H3N2	Positive
EPI_ISL_118107	A/HungYen/HY6/2007	H3N2	Positive
EPI_ISL_29497	A/Hong_Kong/HKU68/2005	H3N2	Positive
EPI_ISL_147986	A/Boston/DOA2-138/2012	H3N2	Positive
EPI_ISL_90512	A/Singapore/NHRC0008/2003	H3N2	Positive
EPI_ISL_114452	A/Memphis/2/1990	H3N2	Positive
EPI_ISL_114869	A/Stockholm/13/1992	H3N2	Positive
EPI_ISL_115354	A/Netherlands/165/1993	H3N2	Positive
EPI_ISL_8669	A/Hong_Kong/14/1974	H3N2	Positive
EPI_ISL_8672	A/Hong_Kong/24/1985	H3N2	Positive
EPI_ISL_235299	A/Puerto_Maldonado/FPM00365/2011	H3N2	Positive
EPI_ISL_5391	A/New_York/632/1996	H3N2	Positive
EPI_ISL_309455	A/Swansea/7754/2018	H3N2	Positive
EPI_ISL_234469	A/Michigan/87/2016	H3N2	Positive
EPI_ISL_9165	A/Canterbury/80/2002	H3N2	Positive
EPI_ISL_20828	A/Hong_Kong/1-2-MA21-2/1968	H3N2	Positive
EPI_ISL_309430	A/Bridgend/4379/2018	H3N2	Positive
EPI_ISL_937	A/Leningrad/360/1986	H3N2	Positive
EPI_ISL_120399	A/Malaysia/1681079/2006	H3N2	Positive
EPI_ISL_8987	A/Hong_Kong/4/1984	H3N2	Positive
EPI_ISL_107930	A/England/484/2003	H3N2	Positive
EPI_ISL_114245	A/Oslo/13676/1983	H3N2	Positive
EPI_ISL_327918	A/Mold/7024/2017	H3N2	Positive

EPI_ISL_309445	A/Swansea/5913/2018	H3N2	Positive
EPI_ISL_6482	A/Queensland/48/2004	H3N2	Positive
EPI_ISL_147171	A/Hong_Kong/JY2/1968	H3N2	Positive
EPI_ISL_90513	A/Singapore/NHRC0007/2003	H3N2	Positive
EPI_ISL_114868	A/Stockholm/12/1992	H3N2	Positive
EPI_ISL_8668	A/Hong_Kong/46/1980	H3N2	Positive
EPI_ISL_5490	A/New_York/682/1995	H3N2	Positive
EPI_ISL_5403	A/New_York/672/1995	H3N2	Positive
EPI_ISL_105790	A/Kunming/1-Va10/2005	H3N2	Positive
EPI_ISL_5392	A/New_York/635/1996	H3N2	Positive
EPI_ISL_9254	A/Canterbury/38/2000	H3N2	Positive
EPI_ISL_84455	A/Netherlands/213/2003	H3N2	Positive
EPI_ISL_4278	A/Memphis/2/1990	H3N2	Positive
EPI_ISL_159598	A/Iowa/04/2013	H3N2	Positive
EPI_ISL_7542	A/Auckland/598/2000	H3N2	Positive
EPI_ISL_294269	A/Maryland/55/2017	H3N2	Positive
EPI_ISL_113966	A/Bilthoven/2600/1975	H3N2	Positive
EPI_ISL_115410	A/Shangdong/9/1993	H3N2	Positive
EPI_ISL_10115	A/TW/229/03	H3N2	Positive
EPI_ISL_6708	A/New_York/187/2000	H3N2	Positive
EPI_ISL_114453	A/Memphis/5/1990	H3N2	Positive
EPI_ISL_5178	A/New_York/580/1996	H3N2	Positive
EPI_ISL_152	A/Memphis/1/71	H3N2	Positive
EPI_ISL_9045	A/Memphis/3/1973	H3N2	Positive
EPI_ISL_10116	A/TW/3286/03	H3N2	Positive
EPI_ISL_313954	A/Neath/7855/2017	H3N2	Positive
EPI_ISL_6666	A/New_York/198/2003	H3N2	Positive
EPI_ISL_6446	A/New_York/701/1995	H3N2	Positive
EPI_ISL_129022	A/Wisconsin/23/2012	H3N2	Positive
EPI_ISL_173280	A/Sydney/2/1993	H3N2	Positive
EPI_ISL_115515	A/Hong_Kong/42/1996	H3N2	Positive
EPI_ISL_78573	A/Peru/WRAIR1508P/2007	H3N2	Positive
EPI_ISL_152184	A/Peru/PER041/2011	H3N2	Positive
EPI_ISL_8588	A/Memphis/1/1986	H3N2	Positive
EPI_ISL_176325	A/Gunma/14G005/2015	H3N2	Positive
EPI_ISL_115698	A/Bangkok/1/1979	H3N2	Positive
EPI_ISL_5476	A/New_York/759/1993	H3N2	Positive
EPI_ISL_114559	A/Lyon/24222/1991	H3N2	Positive
EPI_ISL_114456	A/Victoria/2/1990	H3N2	Positive
EPI_ISL_6863	A/Queensland/44/2004	H3N2	Positive
EPI_ISL_5518	A/New_York/666/1995	H3N2	Positive
EPI_ISL_102662	A/Hong_Kong/H090-692-V10/2009	H3N2	Positive
EPI_ISL_5057	A/New_York/29/2003	H3N2	Positive
EPI_ISL_154511	A/Wisconsin/67/2005	H3N2	Positive
EPI_ISL_6713	A/New_York/263/1999	H3N2	Positive
EPI_ISL_5604	A/New_York/754/1993	H3N2	Positive
EPI_ISL_114295	A/Atlanta/3572/1988	H3N2	Positive
EPI_ISL_158679	A/Tennessee/F2084c83/2011	H3N2	Positive
EPI_ISL_329972	A/Delaware/33/2017	H3N2	Positive
EPI_ISL_6205	A/Western_Australia/58/2004	H3N2	Positive
EPI_ISL_115355	A/Netherlands/357/1993	H3N2	Positive
EPI_ISL_5489	A/New_York/677/1995	H3N2	Positive
EPI_ISL_98708	A/Hong_Kong/HKU104/2005	H3N2	Positive
EPI_ISL_327405	A/Cardiff/9548/2018	H3N2	Positive
EPI_ISL_77624	A/California/VRDL357/2009	H3N2	Positive
EPI_ISL_98694	A/Hong_Kong/HKU121/2004	H3N2	Positive
EPI_ISL_20814	A/Hong_Kong/1-1-MA-20E/1968	H3N2	Positive
EPI_ISL_173301	A/Vietnam/13V_H3-4/2013	H3N2	Positive

EPI_ISL_115110	A/Guangdong/25/1993	H3N2	Positive
EPI_ISL_5041	A/New_York/87/2002	H3N2	Positive
EPI_ISL_108258	A/Boston/DOA85/2011	H3N2	Positive
EPI_ISL_8699	A/Hong_Kong/5/83	H3N2	Positive
EPI_ISL_107710	A/England/4024313/2003	H3N2	Positive
EPI_ISL_9205	A/Canterbury/206/2005	H3N2	Positive
EPI_ISL_288167	A/Victoria/5064/2014	H3N2	Positive
EPI_ISL_5519	A/New_York/667/1995	H3N2	Positive
EPI_ISL_115459	A/Finland/338/1995	H3N2	Positive
EPI_ISL_173048	A/Myanmar/13M105/2013	H3N2	Positive
EPI_ISL_263262	A/Rio_Grande/LACENRS-3171/2013	H3N2	Positive
EPI_ISL_6453	A/Western_Australia/24/2002	H3N2	Positive
EPI_ISL_27622	A/Shangdong/9/1993	H3N2	Positive
EPI_ISL_288370	A/Victoria/187/2012	H3N2	Positive
EPI_ISL_8616	A/Memphis/1/90	H3N2	Positive
EPI_ISL_8225	A/New_York/153/1999	H3N2	Positive
EPI_ISL_9146	A/Memphis/4/1980	H3N2	Positive
EPI_ISL_20831	A/Hong_Kong/1-5-MA21-2/1968	H3N2	Positive
EPI_ISL_102700	A/Hong_Kong/H090-710-V10/2009	H3N2	Positive
EPI_ISL_115111	A/Lyon/672/1993	H3N2	Positive
EPI_ISL_5393	A/New_York/636/1996	H3N2	Positive
EPI_ISL_6631	A/New_York/177/1999	H3N2	Positive
EPI_ISL_235206	A/Jakarta/FLUEJKSV0024/2012	H3N2	Positive
EPI_ISL_94554	A/Beijing-Xicheng/1293/2011	H3N2	Positive
EPI_ISL_5303	A/New_York/625/1996	H3N2	Positive
EPI_ISL_5001	A/New_York/47/2003	H3N2	Positive
EPI_ISL_327694	A/Cardiff/9945/2018	H3N2	Positive
EPI_ISL_309447	A/Pwllheli/5246/2018	H3N2	Positive
EPI_ISL_120337	A/Malaysia/10111/1996	H3N2	Positive
EPI_ISL_195621	A/Quebec/26-031205/2005	H3N2	Positive
EPI_ISL_152450	A/Peru/PER174/2010	H3N2	Positive
EPI_ISL_113069	A/Netherlands/312/2003	H3N2	Positive
EPI_ISL_5394	A/New_York/641/1996	H3N2	Positive
EPI_ISL_114556	A/Lyon/1594/1991	H3N2	Positive
EPI_ISL_107404	A/England/539/2003	H3N2	Positive
EPI_ISL_9051	A/New_York/214/2003	H3N2	Positive
EPI_ISL_172633	A/Myanmar/13M056/2013	H3N2	Positive
EPI_ISL_115768	A/Oklahoma/5/1988	H3N2	Positive
EPI_ISL_76486	A/Philippines/2-MA/1982	H3N2	Positive
EPI_ISL_118273	A/HaNoi/Q769/2007	H3N2	Positive
EPI_ISL_129616	A/Indiana/138/2012	H3N2	Positive
EPI_ISL_309459	A/Swansea/9391/2018	H3N2	Positive
EPI_ISL_115412	A/Yamagata/56/1993	H3N2	Positive
EPI_ISL_5299	A/New_York/613/1996	H3N2	Positive
EPI_ISL_145068	A/Indiana/11/2013	H3N2	Positive
EPI_ISL_107618	A/England/909/2003	H3N2	Positive
EPI_ISL_8173	A/New_York/329/1999	H3N2	Positive
EPI_ISL_8985	A/Hong_Kong/50/1972	H3N2	Positive
EPI_ISL_132417	A/Mexico/InDRE835/2003	H3N2	Positive
EPI_ISL_152259	A/Peru/PER065/2010	H3N2	Positive
EPI_ISL_114345	A/Atlanta/211/1989	H3N2	Positive
EPI_ISL_66096	A/England/72	H3N2	Positive
EPI_ISL_5409	A/Waikato/1/2000	H3N2	Positive
EPI_ISL_5485	A/New_York/639/1995	H3N2	Positive
EPI_ISL_129196	A/Boston/DOA94/2012	H3N2	Positive
EPI_ISL_129418	A/Indiana/147/2012	H3N2	Positive
EPI_ISL_232048	A/Michigan/83/2016	H3N2	Positive
EPI_ISL_3767	A/Wyoming/03/2003	H3N2	Positive

EPI_ISL_115414	A/Hong_Kong/56/1994	H3N2	Positive
EPI_ISL_9050	A/Nanjing/36/1983	H3N2	Positive
EPI_ISL_235232	A/Moscow/1514A00504307T_CP2/2011	H3N2	Positive
EPI_ISL_6527	A/Queensland/42/2004	H3N2	Positive
EPI_ISL_5037	A/New_York/22/2003	H3N2	Positive
EPI_ISL_5398	A/New_York/659/1994	H3N2	Positive
EPI_ISL_125877	A/Sichuan/2/1987	H3N2	Positive
EPI_ISL_250205	A/Nevada/20/2014	H3N2	Positive
EPI_ISL_111020	A/Madrid/G102/1993	H3N2	Positive
EPI_ISL_114297	A/Netherlands/450/1988	H3N2	Positive
EPI_ISL_7634	A/New_York/UR06-0510/2007	H3N2	Positive
EPI_ISL_5520	A/New_York/668/1995	H3N2	Positive
EPI_ISL_114873	A/Victoria/68/1992	H3N2	Positive
EPI_ISL_6156	A/New_York/737/1994	H3N2	Positive
EPI_ISL_102623	A/Hong_Kong/H090-671-V10/2009	H3N2	Positive
EPI_ISL_120377	A/Malaysia/29197/2004	H3N2	Positive
EPI_ISL_5290	A/New_York/578/1996	H3N2	Positive
EPI_ISL_113037	A/Bilthoven/21801/1971	H3N2	Positive
EPI_ISL_5337	A/New_York/733/1994	H3N2	Positive
EPI_ISL_120356	A/Malaysia/13246/1997	H3N2	Positive
EPI_ISL_118540	A/Boston/DOA16/2011	H3N2	Positive
EPI_ISL_90539	A/California/NHRC0006/2003	H3N2	Positive
EPI_ISL_90541	A/Singapore/NHRC0004/2003	H3N2	Positive
EPI_ISL_5002	A/New_York/48/2003	H3N2	Positive
EPI_ISL_114298	A/Stockholm/12/1988	H3N2	Positive
EPI_ISL_29651	A/Hong_Kong/HKU6/2004	H3N2	Positive
EPI_ISL_107697	A/England/602/2003	H3N2	Positive
EPI_ISL_152751	A/Peru/PER317/2010	H3N2	Positive
EPI_ISL_263302	A/Porto_Alegre/LACENRS-921/2015	H3N2	Positive
EPI_ISL_6641	A/New_York/141/1999	H3N2	Positive
EPI_ISL_5683	A/New_York/3/2006	H3N2	Positive
EPI_ISL_25041	A/Siena/4/1990	H3N2	Positive
EPI_ISL_8169	A/New_York/320/1999	H3N2	Positive
EPI_ISL_66170	A/Thailand/CU-B657/2009	H3N2	Positive
EPI_ISL_5152	A/New_York/565/1996	H3N2	Positive
EPI_ISL_114017	A/Amsterdam/1609/1977	H3N2	Positive
EPI_ISL_10119	A/TW/875/04	H3N2	Positive
EPI_ISL_5522	A/New_York/691/1995	H3N2	Positive
EPI_ISL_129428	A/Ohio/57/2012	H3N2	Positive
EPI_ISL_129552	A/Indiana/115/2012	H3N2	Positive
EPI_ISL_113967	A/Bilthoven/2813/1975	H3N2	Positive
EPI_ISL_152496	A/Peru/PER399/2012	H3N2	Positive
EPI_ISL_5396	A/New_York/654/1994	H3N2	Positive
EPI_ISL_114201	A/Netherlands/241/1982	H3N2	Positive
EPI_ISL_114399	A/Victoria/1/1989	H3N2	Positive
EPI_ISL_5580	A/New_York/736/1994	H3N2	Positive
EPI_ISL_114197	A/Texas/1/1977	H3N2	Positive
EPI_ISL_8674	A/Hong_Kong/7/1985	H3N2	Positive
EPI_ISL_113916	A/Port_Chalmers/1/1973	H3N2	Positive
EPI_ISL_309469	A/Cardiff/8360/2018	H3N2	Positive
EPI_ISL_94618	A/Liaoning-Tiedong/1153/2011	H3N2	Positive
EPI_ISL_327400	A/Penarth/8862/2018	H3N2	Positive
EPI_ISL_189629	A/Houston/JMM_166/2013	H3N2	Positive
EPI_ISL_94555	A/Beijing-Xicheng/1409/2011	H3N2	Positive
EPI_ISL_117162	A/HaNoi/662/2004	H3N2	Positive
EPI_ISL_10112	A/TW/872/02	H3N2	Positive
EPI_ISL_176850	A/Lebanon/14L78/2014	H3N2	Positive
EPI_ISL_117093	A/HaNoi/BM767/2003	H3N2	Positive

EPI_ISL_143165	A/Victoria/JY2/1968	H3N2	Positive
EPI_ISL_5622	A/New_York/660/1995	H3N2	Positive
EPI_ISL_114398	A/Singapore/40/1989	H3N2	Positive
EPI_ISL_132418	A/Mexico/InDRE940/2003	H3N2	Positive
EPI_ISL_173279	A/Victoria/186/1982	H3N2	Positive
EPI_ISL_114504	A/Lyon/1149/1991	H3N2	Positive
EPI_ISL_9171	A/Canterbury/409/2003	H3N2	Positive
EPI_ISL_7037	A/Memphis/3/1971	H3N2	Positive
EPI_ISL_6202	A/Western_Australia/55/2004	H3N2	Positive
EPI_ISL_189227	A/Houston/JMM_101/2013	H3N2	Positive
EPI_ISL_5440	A/New_York/689/1995	H3N2	Positive
EPI_ISL_2466	A/Hong_Kong/1774/99	H3N2	Positive
EPI_ISL_67253	A/New_York/2/2003	H3N2	Positive
EPI_ISL_8984	A/Hong_Kong/3/1969	H3N2	Positive
EPI_ISL_8599	A/Christchurch/15/2004	H3N2	Positive
EPI_ISL_9246	A/Canterbury/238/2005	H3N2	Positive
EPI_ISL_98699	A/Hong_Kong/HKU116/2004	H3N2	Positive
EPI_ISL_277241	A/Ohio/22/2017	H3N2	Positive
EPI_ISL_309465	A/Newport/8717/2018	H3N2	Positive
EPI_ISL_263156	A/Frederico_Westphalen/LACENRS-2130/2012	H3N2	Positive
EPI_ISL_5441	A/New_York/709/1994	H3N2	Positive
EPI_ISL_128152	A/Indiana/59/2012	H3N2	Positive
EPI_ISL_5294	A/New_York/608/1996	H3N2	Positive
EPI_ISL_6896	A/Albany/3/1967	H2N2	Positive
EPI_ISL_170560	A/Shanghai/202/1957	H2N2	Positive
EPI_ISL_22830	A/Singapore/1-MA12D/1957	H2N2	Positive
EPI_ISL_19666	A/Cottbus/1/1964	H2N2	Positive
EPI_ISL_170557	A/Georgia/1/1963	H2N2	Positive
EPI_ISL_235	A/Berkeley/1/1968	H2N2	Positive
EPI_ISL_130363	A/Cornell/1001/1967	H2N2	Positive
EPI_ISL_169836	A/Leningrad/134/17/57	H2N2	Positive
EPI_ISL_130408	A/ITS/1/1957	H2N2	Positive
EPI_ISL_6895	A/Albany/7/1967	H2N2	Positive
EPI_ISL_19664	A/Berlin/3/1964	H2N2	Positive
EPI_ISL_170556	A/Beijing/12/1964	H2N2	Positive
EPI_ISL_7062	A/Albany/1/1958	H2N2	Positive
EPI_ISL_19668	A/Tashkent/1046/1967	H2N2	Positive
EPI_ISL_130402	A/Montevideo/2208/1967	H2N2	Positive
EPI_ISL_7061	A/Albany/22/1957	H2N2	Positive
EPI_ISL_170559	A/Leningrad/29/1963	H2N2	Positive
EPI_ISL_7076	A/Albany/1/1960	H2N2	Positive
EPI_ISL_84895	A/Netherlands/056H1/1960	H2N2	Positive
EPI_ISL_27595	A/Korea/426/1968	H2N2	Positive
EPI_ISL_6894	A/Albany/6/1967	H2N2	Positive
EPI_ISL_8314	A/Canada/720/05	H2N2	Positive
EPI_ISL_7082	A/Albany/4/1967	H2N2	Positive
EPI_ISL_130406	A/England/1/1961	H2N2	Positive
EPI_ISL_3294	A/Korea/426/68	H2N2	Positive
EPI_ISL_130405	A/Korea/426/1968	H2N2	Positive
EPI_ISL_6893	A/Albany/26/1957	H2N2	Positive
EPI_ISL_25025	A/Netherlands/56/1963	H2N2	Positive
EPI_ISL_19669	A/Johannesburg/617/1967	H2N2	Positive
EPI_ISL_6913	A/Albany/8/1967	H2N2	Positive
EPI_ISL_6912	A/Albany/1/1959	H2N2	Positive
EPI_ISL_130404	A/Ann_Arbor/7/1967	H2N2	Positive
EPI_ISL_182214	A/Victoria/5/1968	H2N2	Positive
EPI_ISL_6971	A/Albany/1/1968	H2N2	Positive
EPI_ISL_166563	A/Netherlands/B1/1968	H2N2	Positive

EPI_ISL_19665	A/Potsdam/2/1965	H2N2	Positive
EPI_ISL_25007	A/Czech_Republic/1/1966	H2N2	Positive
EPI_ISL_19667	A/Guiyang/1/1957	H2N2	Positive
EPI_ISL_6897	A/Albany/2/1968	H2N2	Positive
EPI_ISL_69298	A/North_Carolina/1/1963	H2N2	Positive
EPI_ISL_6910	A/Albany/9/1967	H2N2	Positive
EPI_ISL_221825	A/Minnesota/45/2016	H1N2	Positive
EPI_ISL_330080	A/Colorado/16/2017	H1N2	Positive
EPI_ISL_320688	A/Ohio/24/2018	H1N2	Positive
EPI_ISL_320690	A/Michigan/383/2018	H1N2	Positive
EPI_ISL_320689	A/California/58/2018	H1N2	Positive
EPI_ISL_99810	A/Minnesota/19/2011	H1N2	Positive
EPI_ISL_329967	A/Ohio/25/2018	H1N2	Positive
EPI_ISL_8702	A/New_York/400/2003	H1N2	Positive
EPI_ISL_329970	A/Ohio/28/2018	H1N2	Positive
EPI_ISL_8694	A/New_York/489/2003	H1N2	Positive
EPI_ISL_329968	A/California/62/2018	H1N2	Positive
EPI_ISL_329969	A/California/63/2018	H1N2	Positive
EPI_ISL_230638	A/Minnesota/70/2016	H1N2	Positive
EPI_ISL_30055	A/Michigan/09/2007	H1N2	Positive
EPI_ISL_238918	A/Iowa/32/2016	H1N2	Positive
EPI_ISL_230639	A/Wisconsin/71/2016	H1N2	Positive
EPI_ISL_6423	A/New_York/C1/2003	H1N2	Positive
EPI_ISL_126260	A/Minnesota/19/2011	H1N2	Positive
EPI_ISL_129684	A/Minnesota/14/2012	H1N2	Positive
EPI_ISL_277244	A/Ohio/35/2017	H1N2	Positive
EPI_ISL_320686	A/Michigan/382/2018	H1N2	Positive
EPI_ISL_320687	A/Michigan/384/2018	H1N2	Positive
EPI_ISL_277243	A/Ohio/24/2017	H1N2	Positive
EPI_ISL_304183	A/Netherlands/10407/2018	H1N2	Positive
EPI_ISL_126261	A/Michigan/09/2007	H1N2	Positive
EPI_ISL_6746	A/New_York/78/2002	H1N2	Positive
EPI_ISL_7889	A/California/UR06-0585/2007	H1N1	Positive
EPI_ISL_69297	A/New_Jersey/1976	H1N1	Positive
EPI_ISL_10251	A/Taiwan/01/1986	H1N1	Positive
EPI_ISL_179403	A/Ohio/09/2015	H1N1	Positive
EPI_ISL_27598	A/Siena/14/1995	H1N1	Positive
EPI_ISL_7819	A/Kentucky/UR06-0027/2007	H1N1	Positive
EPI_ISL_33556	A/Yangon/M285/2008	H1N1	Positive
EPI_ISL_69244	A/AA/Huston/1945	H1N1	Positive
EPI_ISL_70158	A/Gunma/07G006/2008	H1N1	Positive
EPI_ISL_167572	A/Helsinki/743M/2014	H1N1	Positive
EPI_ISL_7881	A/North_Carolina/UR06-0011/2006	H1N1	Positive
EPI_ISL_143168	A/Bellamy/JY2/1942	H1N1	Positive
EPI_ISL_123187	A/Christchurch/4/2001	H1N1	Positive
EPI_ISL_151784	A/Singapore/30L/2007	H1N1	Positive
EPI_ISL_6509	A/South_Australia/30/2000	H1N1	Positive
EPI_ISL_129179	A/Missouri/12/2012	H1N1	Positive
EPI_ISL_70176	A/Niigata/08F093/2009	H1N1	Positive
EPI_ISL_93599	A/Taiwan/6723/2008	H1N1	Positive
EPI_ISL_69245	A/AA/Marton/1943	H1N1	Positive
EPI_ISL_69300	A/Phila/1935	H1N1	Positive
EPI_ISL_6367	A/South_Australia/45/2000	H1N1	Positive
EPI_ISL_87220	A/Boston/58/2009	H1N1	Positive
EPI_ISL_5319	A/New_York/653/1996	H1N1	Positive
EPI_ISL_145699	A/Singapore/49O/2007	H1N1	Positive
EPI_ISL_63397	A/South_Korea/AF10/2008	H1N1	Positive
EPI_ISL_69296	A/New_Jersey/11/1976	H1N1	Positive

EPI_ISL_8264	A/New_York/233/2000	H1N1	Positive
EPI_ISL_7983	A/Oklahoma/UR06-0241/2007	H1N1	Positive
EPI_ISL_7789	A/Albany/8/1979	H1N1	Positive
EPI_ISL_5278	A/New_York/607/1995	H1N1	Positive
EPI_ISL_71056	A/Saskatchewan/5131/2009	H1N1	Positive
EPI_ISL_123173	A/Florida/2/1993	H1N1	Positive
EPI_ISL_7823	A/Vermont/UR06-0035/2007	H1N1	Positive
EPI_ISL_87190	A/Boston/18/2009	H1N1	Positive
EPI_ISL_132867	A/New_Jersey/8/1976	H1N1	Positive
EPI_ISL_6836	A/Memphis/54/1983	H1N1	Positive
EPI_ISL_176752	A/Ulaanbaatar/1735/2009	H1N1	Positive
EPI_ISL_6905	A/Singapore/6/1986	H1N1	Positive
EPI_ISL_80810	A/California/VRDL270/2009	H1N1	Positive
EPI_ISL_7944	A/Virginia/UR06-0562/2007	H1N1	Positive
EPI_ISL_84910	A/Liverpool/1951	H1N1	Positive
EPI_ISL_87215	A/Boston/45/2009	H1N1	Positive
EPI_ISL_27596	A/Siena/10/1989	H1N1	Positive
EPI_ISL_7918	A/Ohio/UR06-0121/2007	H1N1	Positive
EPI_ISL_69268	A/Hickox/1940	H1N1	Positive
EPI_ISL_7800	A/Texas/UR06-0468/2007	H1N1	Positive
EPI_ISL_120421	A/Malaysia/14817/1997	H1N1	Positive
EPI_ISL_146761	A/Arkansas/14/2013	H1N1	Positive
EPI_ISL_6410	A/Nanchang/14/1996	H1N1	Positive
EPI_ISL_120422	A/Malaysia/15042/1998	H1N1	Positive
EPI_ISL_70160	A/Hokkaido/07H007/2007	H1N1	Positive
EPI_ISL_5169	A/FortMonmouth/1/1947	H1N1	Positive
EPI_ISL_7808	A/Texas/UR06-0397/2007	H1N1	Positive
EPI_ISL_149433	A/Iowa/02/2009	H1N1	Positive
EPI_ISL_6436	A/Memphis/27/1983	H1N1	Positive
EPI_ISL_5277	A/New_York/605/1995	H1N1	Positive
EPI_ISL_7788	A/Texas/UR06-0270/2007	H1N1	Positive
EPI_ISL_7982	A/Oregon/UR06-0185/2007	H1N1	Positive
EPI_ISL_8030	A/Illinois/UR06-0096/2007	H1N1	Positive
EPI_ISL_156327	A/New_Zealand/1212d/2009	H1N1	Positive
EPI_ISL_5261	A/Baylor/11515/1982	H1N1	Positive
EPI_ISL_149430	A/Arkansas/15/2013	H1N1	Positive
EPI_ISL_5323	A/Memphis/11/1978	H1N1	Positive
EPI_ISL_6837	A/Memphis/3/1987	H1N1	Positive
EPI_ISL_6974	A/Malaya/302/1954	H1N1	Positive
EPI_ISL_5276	A/New_York/604/1995	H1N1	Positive
EPI_ISL_7947	A/Kentucky/UR06-0449/2007	H1N1	Positive
EPI_ISL_181326	A/Singapore/44T/2007	H1N1	Positive
EPI_ISL_329966	A/Iowa/33/2017	H1N1	Positive
EPI_ISL_147188	A/Fort_Monmouth/1-JY2/1947	H1N1	Positive
EPI_ISL_120445	A/Malaysia/1823766/2007	H1N1	Positive
EPI_ISL_181327	A/Singapore/86D/2007	H1N1	Positive
EPI_ISL_85201	A/Switzerland/5165/2010	H1N1	Positive
EPI_ISL_5566	A/New_York/642/1995	H1N1	Positive
EPI_ISL_5569	A/Memphis/2/1983	H1N1	Positive
EPI_ISL_120419	A/Malaysia/14075/1997	H1N1	Positive
EPI_ISL_5565	A/New_York/630/1995	H1N1	Positive
EPI_ISL_6456	A/South_Australia/26/2000	H1N1	Positive
EPI_ISL_5568	A/Memphis/1/1983	H1N1	Positive
EPI_ISL_143167	A/Malaysia/JY2/1954	H1N1	Positive
EPI_ISL_5433	A/New_York/626/1996	H1N1	Positive
EPI_ISL_100054	A/Wisconsin/28/2011	H1N1	Positive
EPI_ISL_106561	A/HaNoi/BM959/2003	H1N1	Positive
EPI_ISL_138290	A/Hebei-Yuhua/SWL1250/2012_(H1N1v)_	H1N1	Positive

EPI_ISL_202952	A/Minnesota/46/2015	H1N1	Positive
EPI_ISL_124309	A/England/192/2000	H1N1	Positive
EPI_ISL_99209	A/Philippines/WRAIR1736P/2006	H1N1	Positive
EPI_ISL_99217	A/Maracay/WRAIR1729P/2008	H1N1	Positive
EPI_ISL_143164	A/Denver/JY2/1957	H1N1	Positive
EPI_ISL_5279	A/New_York/615/1995	H1N1	Positive
EPI_ISL_6880	A/Memphis/1/2001	H1N1	Positive
EPI_ISL_83267	A/Illinois/09/2007	H1N1	Positive
EPI_ISL_154493	A/Brisbane/59/2007	H1N1	Positive
EPI_ISL_6775	A/Memphis/2/1996	H1N1	Positive
EPI_ISL_7591	A/Kentucky/UR06-0042/2007	H1N1	Positive
EPI_ISL_106498	A/HaNoi/ARI36/2003	H1N1	Positive
EPI_ISL_85649	A/Jiangsu/1/2011	H1N1	Positive
EPI_ISL_106712	A/DaNang/DN365/2008	H1N1	Positive
EPI_ISL_106578	A/HaNoi/HN603/2003	H1N1	Positive
EPI_ISL_5170	A/Baylor/11735/1982	H1N1	Positive
EPI_ISL_123154	A/Nanchang/16A/1999	H1N1	Positive
EPI_ISL_134468	A/SOUTH_AUSTRALIA/162/2012	H1N1	Positive
EPI_ISL_7804	A/California/UR06-0302/2007	H1N1	Positive
EPI_ISL_123180	A/New_Caledonia/20/99	H1N1	Positive
EPI_ISL_7600	A/Illinois/UR06-0248/2007	H1N1	Positive
EPI_ISL_7063	A/Albany/12/1951	H1N1	Positive
EPI_ISL_87225	A/Boston/65/2009	H1N1	Positive
EPI_ISL_6461	A/Memphis/21/1983	H1N1	Positive
EPI_ISL_130407	A/Kw/1/1957	H1N1	Positive
EPI_ISL_66092	A/Bel/1942	H1N1	Positive
EPI_ISL_179404	A/Ohio/09/2015	H1N1	Positive
EPI_ISL_147193	A/Texas/36-JY2/1991	H1N1	Positive
EPI_ISL_7780	A/Texas/UR06-0542/2007	H1N1	Positive
EPI_ISL_70168	A/Nagasaki/07N011/2008	H1N1	Positive
EPI_ISL_30054	A/Ohio/02/2007	H1N1	Positive
EPI_ISL_70157	A/Gunma/07G002/2008	H1N1	Positive
EPI_ISL_5282	A/New_York/621/1995	H1N1	Positive
EPI_ISL_7973	A/Texas/UR06-0503/2007	H1N1	Positive
EPI_ISL_5534	A/Wellington/16/2000	H1N1	Positive
EPI_ISL_71058	A/Saskatchewan/5351/2009	H1N1	Positive
EPI_ISL_5370	A/New_York/645/1995	H1N1	Positive
EPI_ISL_7073	A/USSR/46/1979	H1N1	Positive
EPI_ISL_7873	A/Oregon/UR06-0230/2007	H1N1	Positive
EPI_ISL_156325	A/New_Zealand/1212b/2009	H1N1	Positive
EPI_ISL_5692	A/New_York/616/1995	H1N1	Positive
EPI_ISL_5269	A/Canterbury/65/2001	H1N1	Positive
EPI_ISL_106752	A/Hue/H259/2008	H1N1	Positive
EPI_ISL_123155	A/Kawasaki/6/1986	H1N1	Positive
EPI_ISL_173276	A/Victoria/1/1991	H1N1	Positive
EPI_ISL_156330	A/New_Zealand/1212e/2009	H1N1	Positive
EPI_ISL_345606	A/Franche_Comte/1390/2019	H1N1	Positive
EPI_ISL_66094	A/Denver/57	H1N1	Positive
EPI_ISL_8078	A/California/45/1978	H1N1	Positive
EPI_ISL_6776	A/Memphis/3/1996	H1N1	Positive
EPI_ISL_7050	A/California/10/1978	H1N1	Positive
EPI_ISL_5619	A/New_York/646/1996	H1N1	Positive
EPI_ISL_147189	A/Cameron/JY2/1946	H1N1	Positive
EPI_ISL_7072	A/Albany/1618/1951	H1N1	Positive
EPI_ISL_106711	A/DaNang/DN364/2008	H1N1	Positive
EPI_ISL_83885	A/SYDNEY/217/2010	H1N1	Positive
EPI_ISL_147187	A/Fort_Warren/50-JY2/1950	H1N1	Positive
EPI_ISL_309431	A/Swansea/4496/2018	H1N1	Positive

EPI_ISL_279682	A/Bayern/7/1995	H1N1	Positive
EPI_ISL_5618	A/New_York/643/1995	H1N1	Positive
EPI_ISL_69267	A/Henry/1936	H1N1	Positive
EPI_ISL_8015	A/Texas/UR06-0420/2007	H1N1	Positive
EPI_ISL_7957	A/Virginia/UR06-0114/2007	H1N1	Positive
EPI_ISL_7084	A/Albany/14/1951	H1N1	Positive
EPI_ISL_147168	A/New_Jersey/Swiss/1976	H1N1	Positive
EPI_ISL_106730	A/DaNang/DN432/2008	H1N1	Positive
EPI_ISL_148286	A/Jiangsu/1/2011	H1N1	Positive
EPI_ISL_8014	A/Oregon/UR06-0219/2007	H1N1	Positive
EPI_ISL_147169	A/New_Jersey/Wistar/1976	H1N1	Positive
EPI_ISL_7093	A/Albany/13/1951	H1N1	Positive
EPI_ISL_71057	A/Saskatchewan/5350/2009	H1N1	Positive
EPI_ISL_6768	A/Memphis/41/1983	H1N1	Positive
EPI_ISL_33559	A/Naypyitaw/M783/2008	H1N1	Positive
EPI_ISL_294141	A/Paris/320/2018	H1N1	Positive
EPI_ISL_66099	A/Fort_Worth/50	H1N1	Positive
EPI_ISL_252683	A/Pavia/65/2016	H1N1	Positive
EPI_ISL_143169	A/Hickox/JY2/1940	H1N1	Positive
EPI_ISL_173282	A/Victoria/4/1986	H1N1	Positive
EPI_ISL_5621	A/New_York/656/1995	H1N1	Positive
EPI_ISL_7052	A/New_York/2924-1/1986	H1N1	Positive
EPI_ISL_6853	A/Albany/4835/1948	H1N1	Positive
EPI_ISL_6404	A/New_York/633/1995	H1N1	Positive
EPI_ISL_123156	A/Victoria/36/1988	H1N1	Positive
EPI_ISL_6914	A/Texas/2922-3/1986	H1N1	Positive
EPI_ISL_123176	A/Johannesburg/159/1997	H1N1	Positive
EPI_ISL_5317	A/New_York/638/1995	H1N1	Positive
EPI_ISL_153533	A/Zhejiang/KLED-568/2013(H1N1)	H1N1	Positive
EPI_ISL_7048	A/Albany/4836/1950	H1N1	Positive
EPI_ISL_7608	A/Kentucky/UR06-0188/2007	H1N1	Positive
EPI_ISL_66101	A/Malaysia/54	H1N1	Positive
EPI_ISL_10111	A/TW/130/96	H1N1	Positive
EPI_ISL_69277	A/Iowa/1943	H1N1	Positive
EPI_ISL_7992	A/Kentucky/UR06-0046/2007	H1N1	Positive
EPI_ISL_22625	A/Beijing/262/1995	H1N1	Positive
EPI_ISL_173283	A/Perth/5/1995	H1N1	Positive
EPI_ISL_6833	A/Memphis/1/1979	H1N1	Positive
EPI_ISL_7054	A/South_Australia/44/2000	H1N1	Positive
EPI_ISL_30053	A/Ohio/01/2007	H1N1	Positive
EPI_ISL_120438	A/Malaysia/1718958/2007	H1N1	Positive
EPI_ISL_123175	A/Shanghai/8/1996	H1N1	Positive
EPI_ISL_7585	A/Texas/UR06-0467/2007	H1N1	Positive
EPI_ISL_6774	A/Memphis/12/1986	H1N1	Positive
EPI_ISL_123174	A/Shengzhen/227/1995	H1N1	Positive
EPI_ISL_33557	A/Naypyitaw/M499/2008	H1N1	Positive
EPI_ISL_99227	A/Piura/WRAIR1699P/2008	H1N1	Positive
